# Comprehensive Evolutionary and Expression Analysis of *FCS-Like Zinc finger* Gene Family Yields Insights into Their Origin, Expansion and Divergence

**DOI:** 10.1371/journal.pone.0134328

**Published:** 2015-08-07

**Authors:** Muhammed Jamsheer K, Chanchal Thomas Mannully, Nandu Gopan, Ashverya Laxmi

**Affiliations:** 1 National Institute of Plant Genome Research, Aruna Asaf Ali Road, New Delhi-110067, India; 2 Jawaharlal Nehru Centre for Advanced Scientific Research, Bengaluru-560064, India; Zhejiang A & F university, CHINA

## Abstract

Plant evolution is characterized by frequent genome duplication events. Expansion of habitat resulted in the origin of many novel genes and genome duplication events which in turn resulted in the expansion of many regulatory gene families. The plant-specific *FCS-Like Zinc finger* (*FLZ*) gene family is characterized by the presence of a FCS-Like Zinc finger (FLZ) domain which mediates the protein-protein interaction. In this study, we identified that the expansion of *FLZ* gene family size in different species is correlated with ancestral and lineage-specific whole genome duplication events. The subsequent gene loss found to have a greater role in determining the size of this gene family in many species. However, genomic block duplications played the significant role in the expansion of *FLZ* gene family in some species. Comparison of *Arabidopsis thaliana* and *Oryza sativa FLZ* gene family revealed monocot and dicot specific evolutionary trends. The *FLZ* genes were found to be under high purifying selection. The spatiotemporal expression analyses of *Arabidopsis thaliana FLZ* gene family revealed that majority of the members are highly expressed in reproductive organs. *FLZ* genes were also found to be highly expressed during vegetative-to-reproductive phase transition which is correlated with the proposed role of this gene family in sugar signaling. The comparison of sequence, structural and expression features of duplicated genes identified lineage-specific redundancy and divergence. This extensive evolutionary analysis and expression analysis of *Arabidopsis thaliana FLZ* genes will pave the way for further functional analysis of *FLZ* genes.

## Introduction

Genome duplication and accumulation of variation are prerequisites to the evolution of biological complexity. The origin and diversification of embryophytes are accomplished due to massive changes in the genomes. Whole genome duplications (WGD) and subsequent rearrangements such as gene loss shaped most of the plant genomes [1, 2]. Paleopolyploidy is a recurrent feature in the evolution of land plants [3–6]. Comprehensive phylogenomic analyses revealed that evolution and diversification of seed plants happened due to two ancient WGD events; one predates the divergence of gymnosperms and angiosperms and the other predates the diversification of angiosperms [7]. Similarly, many lineage and species-specific WGD events are revealed in the analysis of many monocot and dicot genomes [1–4, 8–13].

A high percentage of plant genes are grouped into gene families based on sequence similarity features such as the presence of a common protein domain. The expansion and contraction of specific gene families are correlated with the characteristics of the specific species [6, 11, 12, 14–17]. Analysis of gene family evolution in plants and metazoa identified that gene families undergo species-specific contraction and expansion [18, 19]. The expansion of gene numbers due to duplication events and subsequent divergence are major contributors to the evolution of biological complexity [20].

The *F* *CS-* *L* *ike* *Z* *inc-finger* (*FLZ*) gene family is a plant-specific gene family characterized by the presence of a FCS-Like Zinc-finger (FLZ) domain [21]. FLZ domain possesses a typical alpha-beta-alpha topology and mediates the protein-protein interaction. [21,22]. Functional analysis revealed that many members of this gene family is related to plant development, senescence, stress mitigation and sugar signaling [22–27]. Although, the role of many *FLZ* genes in the regulation of plant life is known, not much information is available on the evolutionary aspects of this gene family.

A comprehensive survey of FLZ domain containing proteins from sequenced plant genomes was done in our earlier study [21]. The availability of *Amborella trichopoda* genome, which is a basal angiosperm with no lineage-specific WGD event, enables detailed studies on the evolutionary expansion of gene families in higher angiosperms [3]. In this study, using all these available resources, we tried to construct the evolutionary history of *FLZ* gene family. We analysed the evolutionary expansion of *FLZ* gene family and correlated it with already established genome duplication events. Detailed phylogenetic studies of dicot and monocot models were done and the evolutionary features were compared. The selection pressure on *FLZ* genes was studied within and across the genome. The spatiotemporal expression dynamics of *Arabidopsis thaliana FLZ* gene family was studied and expression divergence of duplicated gene members was analysed.

## Materials and Methods

### Identification of *FLZ* gene family members from sequenced plant genomes

In a previous study, we used a combination of bioinformatics tools for the identification and domain sequence and structure conservation verification of *FLZ* gene family members from 41 sequenced plant genomes [21]. These sequences were used for the evolutionary analysis of *FLZ* gene family in this study. Besides, *FLZ* gene family members from *A*. *trichopoda*, *Musa acuminata*, *Ricinus communis*, *Carica papaya*, *Vitis vinifera*, *Zea mays* and *Medicago truncatula* were identified. The members from *A*. *trichopoda* were identified from NCBI Reference Sequence Database (http://www.ncbi.nlm.nih.gov/refseq/) and Amborella Genome Database (http://www.amborella.org/) by BLASTp using *AthFLZ1* as query [28, 3]. Members from *M*. *acuminata* were identified from The Banana Genome Hub (http://banana-genome.cirad.fr/) using InterPro id IPR007650 [29]. Additional members of *R*. *communis*, *C*. *papaya* and *M*. *truncatula FLZ* gene family were identified by the search in PLAZA v3.0 Dicots database (http://bioinformatics.psb.ugent.be/plaza/versions/plaza_v3_dicots/) using InterPro id IPR007650 [30]. More members of *Z*. *mays* and *V*. *vinifera FLZ* gene family were identified from NCBI Reference Sequence Database (http://www.ncbi.nlm.nih.gov/refseq/) using BLASTp [28]. The sequences were curated by InterProScan5 (http://www.ebi.ac.uk/Tools/pfa/iprscan5/) and ClustalX 2.0 for confirmation of domain integrity [31, 32]. The sequences were further subjected to secondary structure confirmation using Ali2D (http://toolkit.tuebingen.mpg.de/ali2d) [33].

### Phylogenetic analysis

In order to analyse the evolutionary relationship among *FLZ* genes of different species, full-length proteins were used for phylogram construction. The sequences were aligned by ClustaX 2.0 [32]. Both Bayesian inference (BI) and maximum likelihood (ML) methods were used for phylogenetic analysis. MrBayes(v. 3.2.5) was used for BI analysis and MEGA (v. 5.0) was used for ML analysis [34, 35]. ProtTest 2.4 was used for identification of the best suited model of amino acid substitution [36]. Trees generated using BI method used JTT model of amino acid substitution [37]. In all BI analysis, two Metropolis-Coupled Markov Chain Monte Carlo runs each with 16 chains was performed with default settings for 5,000,000 generations. Approximately 200,000 trees were generated during the process and 25% of the generated trees were disregarded as burn-in. The BI trees were edited and visualized using FigTree (v. 1.4.2) [38].

ML trees were constructed with JTT model of amino acid substitution with 1000 replicate bootstrap analysis. The FLZ proteins from different species were named according to their relationship with *A*. *thaliana* FLZ1 protein (S1 Table). The duplication data of *FLZ* gene family was retrieved from PLAZA v. 2.5 (http://bioinformatics.psb.ugent.be/plaza/versions/plaza_v2_5/) which is based on i- ADHoRe program which detects homologous genomic regions [39, 40]. The duplication data was manually curated and verified by analysing the relative position of genes annotated as duplicates by the program in the phylogram of individual species (Data not shown). The chromosomal location of *FLZ* genes were determined by Whole Genome Mapping tool available in PLAZA v. 2.5 (http://bioinformatics.psb.ugent.be/plaza/versions/plaza_v2_5/genome_mapping/index) [39]. The gene and protein structure was constructed using GSDS v2.0 (http://gsds.cbi.pku.edu.cn/) [41]. Phylogenetic tree of species was created using Taxonomy Common Tree tool at NCBI and edited and visualized using FigTree (v. 1.4.2) [38].

### Ka/Ks estimation of homologous genes

Ka/Ks ratio of paralogous and orthologous *FLZ* genes were calculated using PAL2NAL (http://www.bork.embl.de/pal2nal/) which is based on codeml program in PAML [42]. For Ka/Ks estimation of paralogous genes, the gene pairs from *Physcomitrella patens* and *Selaginella moellendorffii* were used. Similarly, paralogous genes from *A*. *thaliana* and *Oryza sativa* were identified from PLAZA v2.5 [39]. The very closely positioned proteins in the species phylogram of *Picea abies* and *A*. *trichopoda* were annotated as putative paralogs and used for Ka/Ks estimation. For Ka/Ks estimation of orthologous gene pairs, orthologous genes of 6 *A*. *thaliana* and *O*. *sativa* genes were identified from species occupying key taxonomic positions from Phytozome (http://www.phytozome.net/) [43]. Orthologous genes of *A*. *thaliana* genes were identified from *P*. *patens*, *S*. *moellendorffii*, *A*. *trichopoda*, *Solanum lycopersicum*, *V*. *vinifera*, *Brassica rapa*, *Arabidopsis lyrata*, *Malus domestica*, *Glycine max*, *Populus trichocarpa* and *O*. *sativa*. Orthologous genes of *O*. *sativa* genes were identified from *P*. *patens*, *S*. *moellendorffii*, *A*. *trichopoda*, *Brachypodium distachyon*, *Hordeum vulgare*, *Panicum virgatum*, *Setaria italica*, *Sorghum bicolor*, *Z*. *mays* and *A*. *thaliana*. The Ka/Ks ratio was estimated from each gene pair and the averaged Ka/Ks ratios were plotted in the graphs. The gene name and the Ka/Ks ratio values of each pair are given in S2 and S3 Tables.

### Other bioinformatics tools used

For multiple sequence alignment for the verification of FLZ domain conservation across the plant kingdom, members from *P*. *patens*, *S*. *moellendorffii*, *P*. *abies*, *A*. *trichopoda*, *A*. *thaliana*, *M*. *truncatula*, *Populus trichocarpa* and *O*. *sativa* were used. The sequences were aligned by ClustalX 2.0 and visualized by Jalview (http://www.jalview.org/) [32, 44]. Novel motifs in the FLZ proteins were identified by MEME (http://meme.nbcr.net/meme/cgi-bin/meme.cgi) and their similarities with known domains were analysed by HH pred (http://toolkit.tuebingen.mpg.de/hhpred) [45, 46].

### Plant material, growth conditions and gene expression analysis

The *A*. *thaliana* Columbia (Col-0) ecotype was used for gene expression study. Seeds were surface sterilized and kept at 4°C for 48h in dark for stratification. The imbibed seeds were placed on square petri plates vertically for germination and growth. The plants were grown in 0.5X MS medium with 1% sucrose and 0.8% agar in climate-regulated growth chambers under 16:8 hours photoperiod with 22°C ± 2°C temperature and 60μmol m^−2^ s^−1^ light intensity. Samples for all seedlings stages were collected from plates and other stages were collected from plants grown in Agro peat-vermiculite (3:1) mixture at the specified climatic conditions mentioned above. The stages were determined by visible phenotype and growth stage-based phenotypic analysis of Boyes et al [47]. At least 20 plants were used for each sample and tissues were pooled together.

RNA isolation and cDNA preparation were done as described previously [48]. 1:50 diluted cDNA samples were used for qRT-PCR with SYBR-Green PCR master mix in 384-well optical reaction plates using Applied Biosystems 7500 Fast Real-Time PCR System as per manufacturer’s protocol (Applied Biosystems, USA). The primers of *FLZ* genes were prepared from the unique region identified in each transcript using PRIMER EXPRESS version v3.0 (Applied Biosystems, USA) with default parameters. *UBQ10* was used as endogenous control and mRNA level of candidate genes were calculated by 2^ΔCt^ method [49]. Primers used for qRT-PCR are listed in S4 Table.

## Results and Discussion

### *FLZ* gene family in different genomes

In a previous study, we employed multiple strategies for the genome-wide identification of *FLZ* genes. A total of 757 *FLZ* genes were reported from 41 sequenced plant genomes [21]. Further, 8 and 43 non-redundant FLZ proteins were identified from *A*. *trichopoda* and *M*. *acuminata* genomes respectively. Additional members of *FLZ* gene family of *R*. *communis*, *C*. *papaya*, *V*. *vinifera*, *M*. *truncatula* and *Z*. *mays* were identified (S5 Table) [30]. It was found that the *FLZ* genes identified in this study also shows the general protein domain organization with single FLZ domain as observed in the previous study. Similarly, FLZ domain in the newly identified proteins also shows the typical alpha-beta-alpha topology which distinguish FLZ domain from the zf-FCS domains from bacteria, virus and metazoa [21].

### Sequence conservation of FLZ proteins

**Fig 1:**
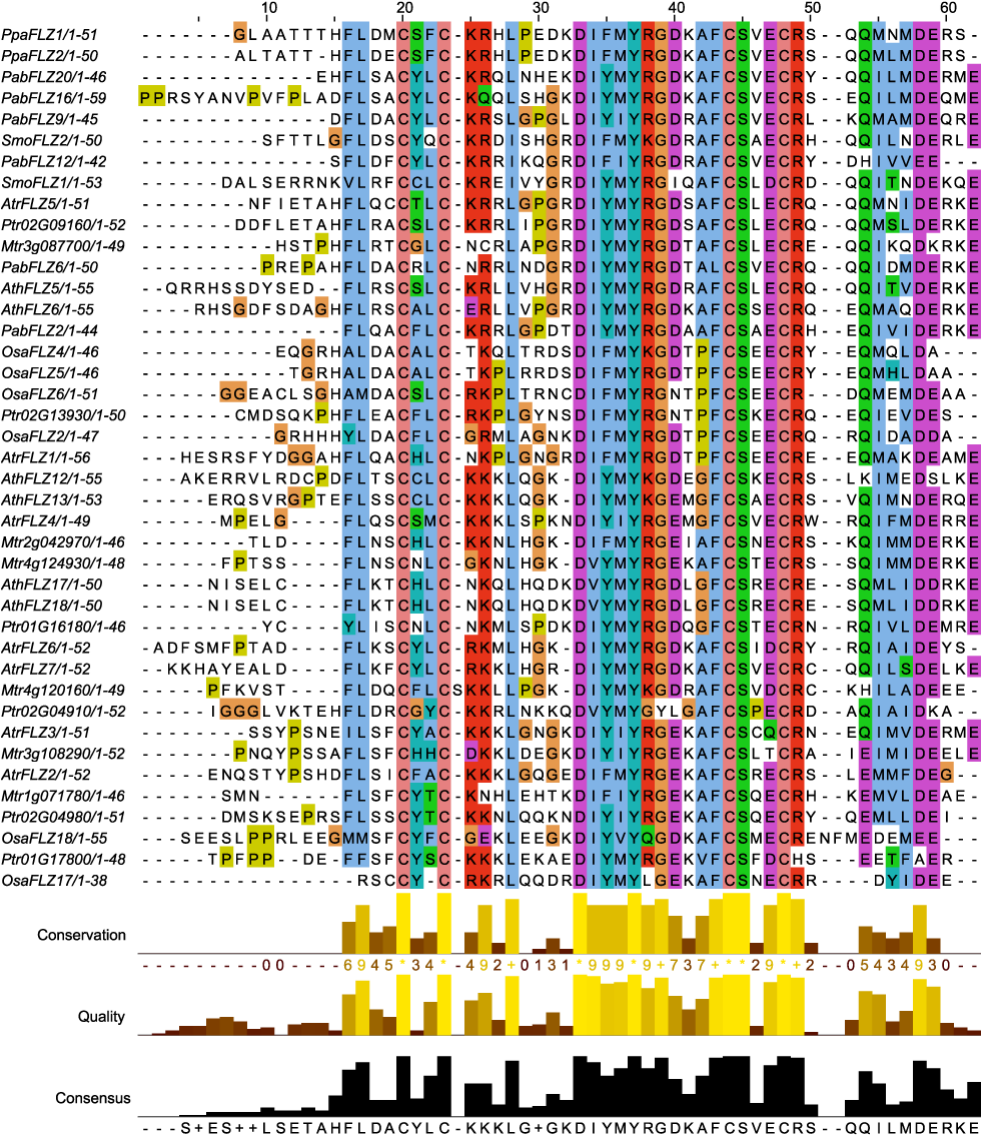
Sequence conservation of FLZ domain. Multiple sequence alignment of FLZ domain from FLZ proteins of selected species is showing conserved residues. The species used for MSA are abbreviated as follows, Ppa, *Physcomitrella patens*; Sma, *Selaginella moellendorffii*; Pab, *Picea abies*; Atr, *Amborella trichopoda*; Ath, *Arabidopsis thaliana*; Mtr, *Medicago truncatula*; Ptr, *Populus trichocarpa*; Osa, *Oryza sativa*.

It was earlier reported that the FLZ domain is highly conserved in sequence as well as secondary structure topology. To confirm the sequence conservation of FLZ domain across the plant kingdom, FLZ proteins from different species which represent key taxonomical divisions were aligned (Fig 1). It was found that apart from the conserved zinc finger signature motif CX_2_CX_17–19_FCSX_2_C, many other residues are highly conserved in the FLZ domain region across the plant kingdom which might be important in protein-protein interaction. Alignment of whole proteins identified that FLZ domain is the most conserved region in these family proteins. However, some localized conserved regions were also found in the alignment (S1 Fig). This prompted us to investigate conserved regions in the FLZ proteins other than FLZ domain. MEME search identified 15 motifs other than FLZ domain with fair degrees of conservation (S6 Table). These novel motifs were subjected to HHpred search to identify their similarity with already known domains (S7 Table). However, it was found that these motifs do not show any significant similarity with already known domains. Although these motifs have no significant relation with known domains, these domains might be evolved later and they might be contributing to the functional divergence of FLZ domain containing proteins.

### Origin and expansion of *FLZ* genes

An extensive search in the sequenced algal genomes could not identify any *FLZ* genes hinting a bryophytic origin of *FLZ* gene family. Two *FLZ* genes were identified from bryophyte *P*. *patens* suggesting a bryophytic origin of *FLZ* genes [21]. The transition from aquatic to the terrestrial life happened due to massive changes in the genome. Comparison of *P*. *patens* genome with genomes of aquatic algae identified an increase in gene family complexity, acquisition of genes required for terrestrial life and loss of genes which are required for the survival in aquatic environments. For example, late embryogenesis abundant proteins, *ABI5*, *HSP70* and genes involved in the processes like cytokinin, auxin, and ethylene signaling are present in *P*. *patens* which enable the plant to survive in the terrestrial environment [6]. Although the molecular functions of the *FLZ* gene family members in general are not well understood, many of the members are attributed to plant growth and development, stress mitigation, sugar signaling and senescence [22–27]. Based on these observations, it can be concluded that similar to the case of genes involved in stress and hormone signaling, the massive transition of habitat possibly facilitated the origin of *FLZ* gene also. The number of *FLZ* genes was found to be same in pteridophyte *S*. *moellendorffii* [21]. There is no evidence for any lineage specific WGD event in *S*. *moellendorffii* genome as the transition from a gametophyte- to a sporophyte-dominated life cycle required comparatively less acquisition of genes [50].

The number of *FLZ* genes was expanded to 8 in *A*. *trichopoda* and 23 in *Picea abies*. An ancestral seed plant specific WGD event predated the spermatophyta diversification must have contributed to the expansion of *FLZ* gene family in *A*. *trichopoda* and *P*. *abies* [7]. Besides this duplication, the higher expansion of *FLZ* gene family in *P*. *abies* compared to *A*. *trichopoda* suggests the possibility of a gymnosperm specific WGD event. However, analysis of *P*. *abies* genome does not provide evidence of any lineage specific WGD event in gymnosperms [51]. Similarly, the EST data analysis of *Pinus taeda* and *Pinus pinaster* implies the absence of Pinophyta-specific WGD events while *Welwitschia mirabilis*, which belongs to Gnetophyta shows an equivocal possibility of a WGD event [52]. As there is no evidence of specific WGD event in Pinophyta lineage, it can be assumed that the expansion of *FLZ* gene family in *P*. *abies* is possibly the result of genomic duplication events. Apart from the ancestral seed plant specific WGD event ζ, another WGD event predated angiosperm diversification known as ε, must also have contributed to the expansion of *FLZ* gene members from 2 in bryophyta and pteridophyta to 8 in *A*. *trichopoda* [3, 7].The absence of lineage-specific WGD event in *A*. *trichopoda* is attributed to its reduction of specific duplication of gene families [3].This must be the reason of reduced number of *FLZ* genes in *A*. *trichopoda* compared to *P*. *abies*.

Phylogenetic analysis of *A*. *trichopoda* and *P*. *abies* FLZ proteins was done to delineate the evolutionary relation among the members. In the phylogram, most of the *A*. *trichopoda* FLZ proteins were found to be positioned apart suggesting high sequence divergence among them (S2A Fig). In comparison, many of the members of *PabFLZ* family formed clusters with short branch lengths further supports the possibility of frequent gene duplication events (S2B Fig). Besides, the slow evolutionary rate in conifers must be the reason for the formation of clusters with short branch lengths in the phylogram [51].

**Fig 2:**
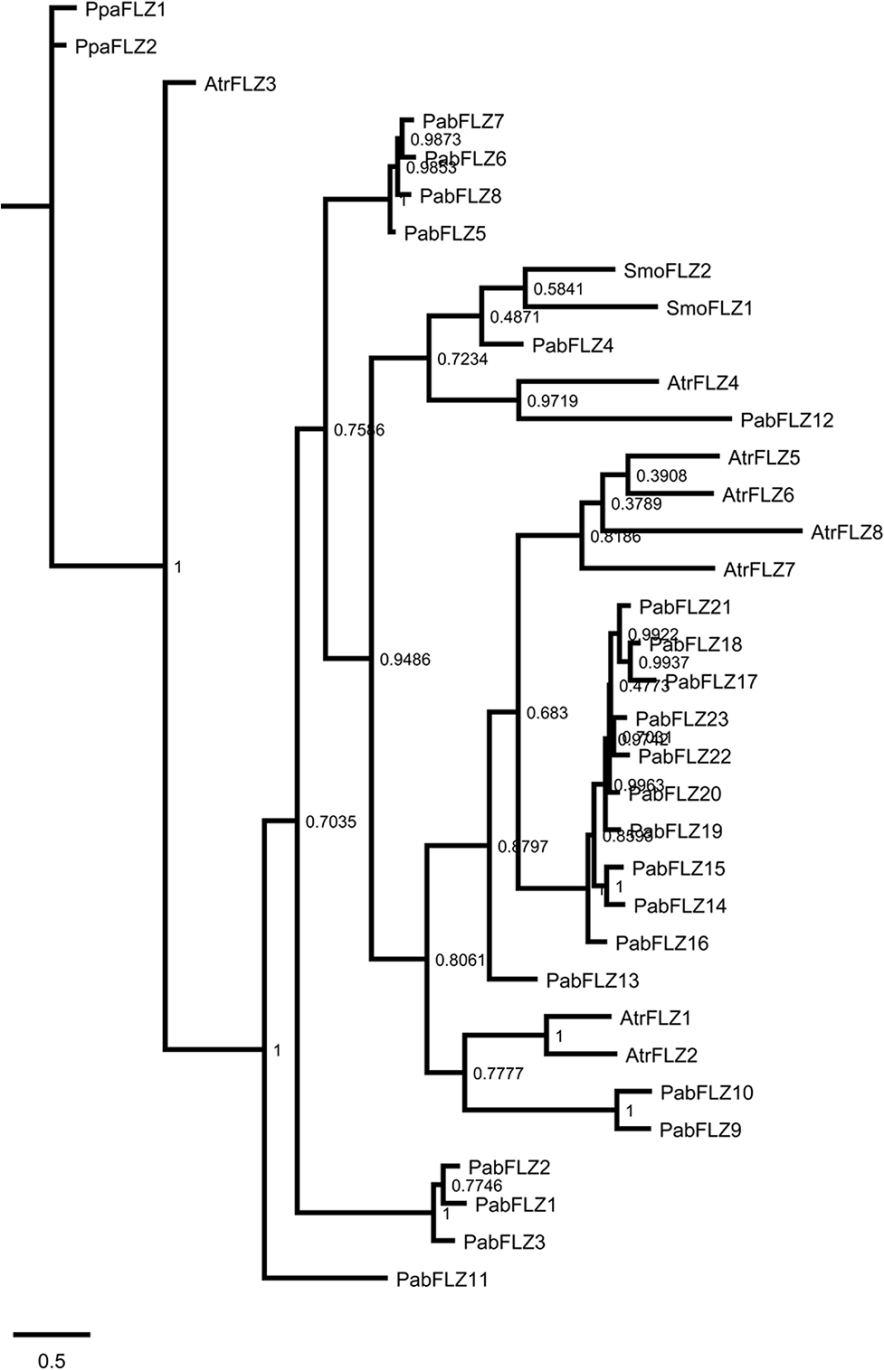
Analysis of phylogenetic origin of FLZ proteins. Phylogram of *P*. *patens*, *S*. *moellendorffii*, *P*. *abies* and *A*. *trichopoda* FLZ proteins was inferred by BI method based on JTT model. The numbers adjacent to the branches represent posteriori probability values. The tree is drawn to scale, with branch lengths measured in the number of amino acid substitutions per site.

The number of *FLZ* genes is highly expanded in angiosperms [21]. In order to elucidate the evolutionary history of *FLZ* gene family prior to its expansion in angiosperms, comparative phylogenetic analysis was performed among *P*. *patens*, *S*. *moellendorffii*, *P*. *abies*, and *A*. *trichopoda* which are belonging to Bryophyta, Pteridophyta, Gymnospermae and Angiospermae respectively (Fig 2). The phylogram separated the proteins into distinct groups. The PpaFLZ and SmoFLZ members were found be placed distant in the phylogram implying the species-specific variations accumulated in *FLZ* genes during plant evolution. Two FLZ proteins of *P*. *patens* were positioned close in a distinct branch of phylogram. *PpaFLZ1* and *PpaFLZ2* showed similar exon-intron structure, protein length and domain organization implying their high similarity in sequence as well as in gene and protein structure (S4A and S4B Fig). Interestingly, the two members of *S*. *moellendorffii* displayed high divergence in both sequence and structure. Both SmoFLZ1 and SmoFLZ2 were found in the neighboring branches; however, their branch distance was found to be more than in the case of *P*. *patens* FLZ proteins (Fig 2). *SmoFLZ1* showed similar exon-intron structure and domain organization like *PpaFLZ* genes while *SmoFLZ2* possesses 3 exons instead of 2 (S4A Fig) Apart from that, the protein size is also considerably decreased in *SmoFLZ2* (S4B Fig).These results suggest that although there is no change in the number of *FLZ* genes in *S*. *moellendorffii*, one of the members accumulated high degrees of sequence as well as the structural variation.

Most of the *P*. *abies* proteins were formed clusters with short branch lengths in the phylogram (Fig 2). These genes mainly contributed to the expansion of *FLZ* gene family in *P*. *abies*. Considering the absence of WGD in Pinophyta, this observation implies the pivotal role of genomic block duplications in the expansion of *FLZ* gene family in *P*. *abies*. Among the *A*. *trichopoda* FLZ proteins, AtrFLZ3 was found to be in a distinct branch more close to *P*. *patens* FLZ proteins suggesting that this protein is very similar to the ancestral FLZ proteins. AtrFLZ5, AtrFLZ6, AtrFLZ7, and AtrFLZ8 formed a distinct clade; however, the branch length was found to be higher among these proteins (Fig 2). The remaining three *A*. *trichopoda* proteins were found to be positioned close to *P*. *abies* proteins suggesting that these proteins are more related to ancestral FLZ proteins.

The exon-intron structure was found to be considerably conserved among most of the *PabFLZ* genes while comparatively more variation in the intron length was observed among *AtrFLZ* genes (S4A Fig). All these results suggest that individual genes accumulated more sequence as well as structural variation in *A*. *trichopoda*. In the case of *P*. *abies*, genomic block duplication played a major role in the expansion of this gene family and the duplicated genes were seen to be very close in the phylogram. The slow evolutionary rate in the conifers must be contributed to this scenario. At the functional level, this suggests the possibility of increased amount of functional redundancy among closely related members in *P*. *abies*.

In order to analyse the evolutionary expansion of *FLZ* genes in angiosperms, the duplication data of *FLZ* genes from selected species was retrieved from PLAZA v2.5 [35]. It was noticed that the increased number of *FLZ* genes in many species is the result of lineage-specific block and tandem duplications (Table 1). The similar number and duplication history of *FLZ* genes in *O*. *sativa*, *B*. *distachyon* and *S*. *bicolor* suggest that the expansion of *FLZ* genes in these species is the result of whole genome duplication predated diversification of Poaceae or before that. The ancient monocot specific τ duplication and σ and ρ duplications which were occurred after the divergence of Poales from Liliales, Zingiberales and Arecales must have contributed to the similar number and duplication history of *FLZ* gene family in these Poaceae species [9,10]. A lineage-specific WGD event in *Z*. *mays* which distinguish this species from its closest relative *S*. *bicolor*, lead to increasing the number of *FLZ* genes to 47 (S5 Table) [53]. Apart from the monocot specific τ duplication, the γ duplication event common to Zingiberales and α and β duplications specific to *Musa* lineage must be the reason for the high expansion of *FLZ* genes in *M*. *acuminata* [9, 10].

**Table 1:** Duplication history of *FLZ* gene family.

Species	TNG	BD	BDN (%)	TD	TDN (%)	TD&BD	TDN&BDN (%)	ND	ND (%)
*Physcomitrella patens*	2	0	0	0	0	0	0	2	100
*Selaginellamoellendorffii*	2	0	0	0	0	0	0	2	100
*Amborella trichopoda*	8	0	0	0	0	0	0	8	100
*Musa acuminata*	43	34	79.07	0	0	0	0	9	20.93
*Oryzasativa*	29	16	55.17	8	27.58	8	27.58	13	44.82
*Brachypodiumdistachyon*	26	17	65.38	10	38.46	10	38.46	9	34.62
*Sorghum bicolor*	29	19	65.52	13	44.83	11	37.93	8	27.59
*Theobroma cacao*	12	4	33.33	0	0	0	0	8	66.66
*Carica papaya*	10	0	0	0	0	0	0	10	100
*Arabidopsis thaliana*	18	12	66.66	2	11.11	0	0	4	22.22
*Populustrichocarpa*	23	16	69.56	0	0	0	0	7	30.43
*Ricinuscommunis*	12	4	33.33	0	0	0	0	8	66.66
*Manihotesculenta*	18	12	66.66	0	0	0	0	6	33.33
*Fragariavesca*	14	6	42.86	0	0	0	0	8	57.14
*Malusdomestica*	22	2	9.09	0	0	0	0	20	90.90
*Glycine max*	37	30	83.33	0	0	0	0	7	18.91
*Medicagotruncatula*	20	15	75	4	20	4	20	5	25
*Lotus japonicus*	9	2	22.22	0	0	0	0	7	77.78

TNG: Total number of genes, BD: Block duplicates, BDN: Block duplication, TD: Tandem duplicates, TDN: Tandem duplication, TD&BD: Tandem & block duplicates, TDN&BDN: Tandem & block duplication, ND: Non-duplicates, NDN: Non-duplication

The evolutionary pattern of *FLZ* genes in the sequenced dicot species shows lineage-specific variations. The *Carica papaya* genome contains 10 non-duplicated *FLZ* genes. A genome triplication prior to core eudicot radiation and the subsequent gene loss must have restricted the increase of *FLZ* gene number from 8 in *A*. *trichopoda* to 10 in this species [1]. The *V*. *vinifera* and *C*. *papaya* genomes have not undergone any recent lineage-specific WGD event [15, 54]. However, the number of *FLZ* genes increased to 14 in *V*. *vinifera* which can be the result of genomic block duplication events (S5 Table). The expansion of *FLZ* genes to 12 in *Theobroma cacao* can be the product of one round of genomic block duplication in two ancestral genes (Table 1). *FLZ* genes in *A*. *thaliana*, which is another member in eurosid II, underwent more duplication. Two lineage-specific WGD after their division from eurosid I and subsequent gene loss and extensive genomic block duplications shaped *A*. *thaliana* genome [4, 55–57]. This could be the reason for the comparatively higher occurrence of duplicated genes in *FLZ* gene family of *A*. *thaliana* with no major leap in the numbers despite two lineage-specific WGD events.

Analysis of the evolutionary history of *FLZ* genes in the malpighiales order of eurosid I clade also confirms the role of duplication and gene loss in the expansion of this gene family. The increase in *FLZ* gene number and higher representation of duplicated genes were observed in *P*. *trichocarpa* and *Manihot esculenta* while only two events of block duplication were observed in *R*. *communis*. The expansion of *FLZ* gene number in *P*. *trichocarpa* can be attributed to the salicoid specific WGD that happened after the divergence of eurosid clades [11]. The absence of lineage-specific WGD can be accounted for the lack of greater expansion of *FLZ* gene family in *R*. *communis* [16]. *M*. *esculenta*, which is another member of the family euphorbiaceae showed a comparatively larger expansion of *FLZ* gene family than in the case of *R*. *communis*. Although the genome of *M*. *esculenta* is not published yet, the increased number and high representation of duplicated genes in the *FLZ* gene family could predict a lineage specific WGD event in *Manihot*. *Fragaria vesca* genome contains 14 *FLZ* genes. Three rounds of genomic block duplication events in *F*. *vesca* must have contributed in the expansion of *FLZ* genes to 14 in this species (Table 1). The absence of WGD in the *Fragaria* lineage can be accounted for the less number of *FLZ* genes in *F*. *vesca* compared to other rosaceae species, *M*. *domestica* [58]. A WGD event 30–45 MYA ago after the divergence from *Fragaria* must be contributed to the expansion of *FLZ* gene family in *M*. *domestica* [12]. In legumes, the high expansion of *FLZ* genes in *G*. *max* must be due to a lineage-specific WGD event that occurred about 13 million years ago [13]. The absence of large-scale duplication events in the galegoid tribe restricted their number in *Lotus japonicus* [2]. However, the number for *FLZ* gene family is found to be high in *M*. *truncatula*. The high rate of genomic block duplications increased gene content and expansion of many gene families in this species [17]. This can be accounted for the expansion of *FLZ* gene family in *M*. *truncatula* despite the absence of lineage-specific WGD events.

Analysis of evolutionary expansion of *FLZ* gene family in spermatophyta clearly implies the role of WGD events. The general and lineage-specific WGD events reported were found to be correlated with the expansion of *FLZ* gene family (S3 Fig). Besides, the *FLZ* gene family size and duplication history of *M*. *esculenta* indicate the possibility of a WGD event in the *Manihot* lineage. Interestingly, analysis of myosin gene family also predicts a WGD event in *Manihot* lineage [59]. From the 8 members in *A*. *trichopoda*, the genome triplication common to eudicots could only marginally increase the number of *FLZ* genes in species with no lineage-specific WGD such as *C*. *papaya*, *V*. *vinifera*, *L*. *japonicus* etc suggesting a high rate of gene loss after this genome triplication. A recent gene family size comparison of Nucleobase–Ascorbate Transporter (NAT) gene family among eudicots yielded similar observation. The number of NAT genes increased from 7 in *A*. *trichopoda* to only 9 in *V*. *vinifera* and *C*. *papaya* despite the basal eudicot genome triplication [60]. Among monocots, the WGD predated to the diversification of species studied lead to the expansion of *FLZ* gene family in general while lineage-specific WGD in *M*. *acuminata* and *Z*. *mays* lead to increased number of genes in these two species. In genomes like *M*. *truncatula* and *P*. *abies*, genomic block duplication was found to be the major contributing factor in the expansion of *FLZ* gene family.

### Phylogenetic analysis of *Arabidopsis thaliana FLZ* gene family

**Fig 3:**
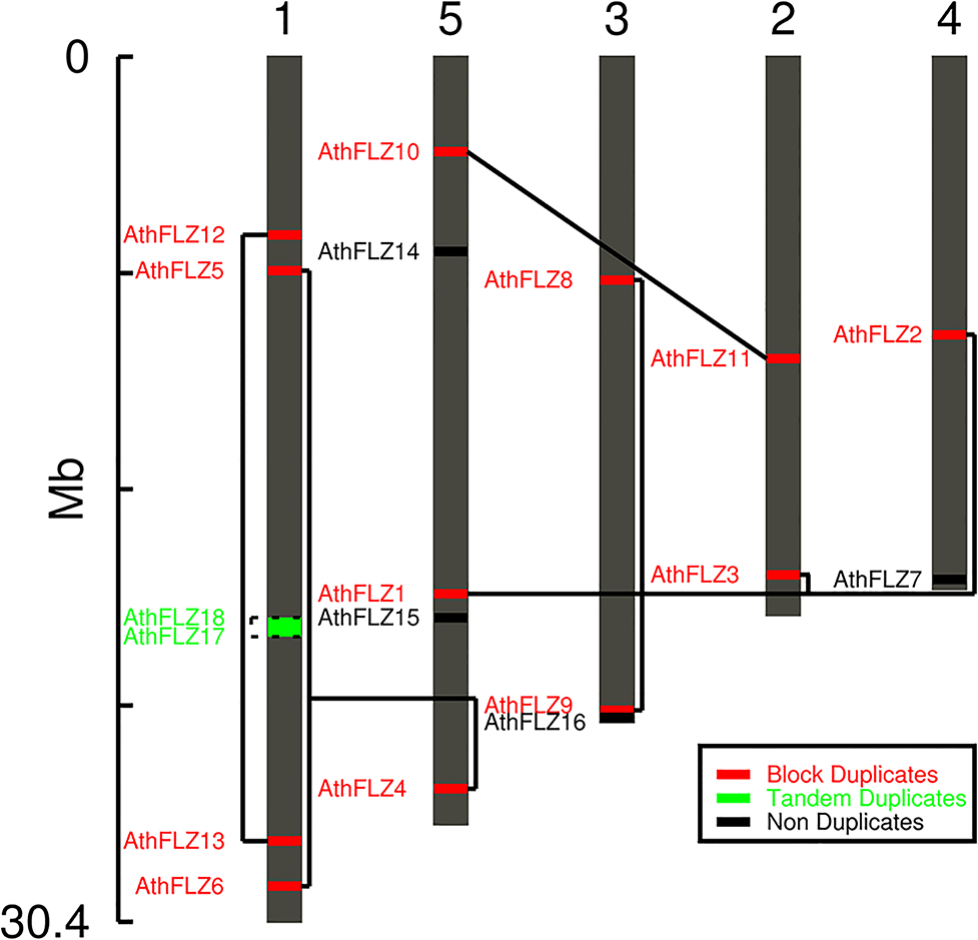
Chromosomal location and evolutionary history of *Arabidopsis thaliana FLZ* gene family. The gene duplication analysis is done using PLAZA v2.5 and verified by the position of duplicates in phylogram. Solid line connects the block duplicates and dotted line connects the tandem duplicates. The numbers indicated at the top represents the chromosome number.

*A*. *thaliana* genome possesses 18 *FLZ* genes which are distributed in all 5 chromosomes (Fig 3). *FLZ* genes were found in all chromosomes; however, segment-specific duplications resulted in unequal distribution of genes. Among 18 members, only 3 members i.e., *AthFLZ7*, *AthFLZ15*, and *AthFLZ16* did not undergo any duplication. *AthFLZ17* and *AthFLZ18* are products of tandem duplication with no divergence in sequence. All other members are products of block duplication events. As discussed earlier, the *A*. *thaliana* is an extensively duplicated genome with subsequent gene loss and differential radiation of many gene families [4].

**Fig 4:**
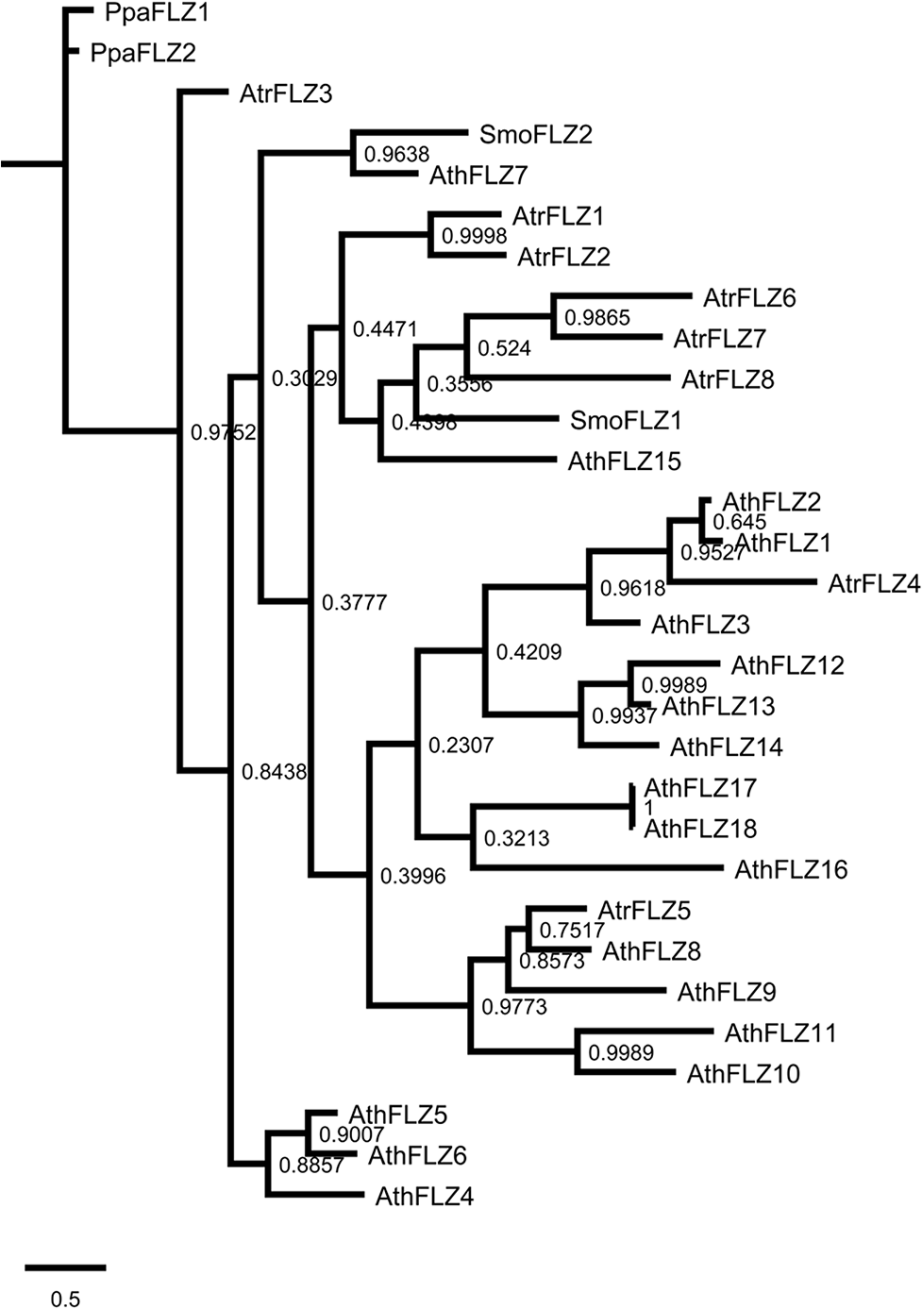
Analysis of phylogenetic origin of *Arabidopsis thaliana* FLZ proteins. Phylogram of *A*. *thaliana*, *P*. *patens*, *S*. *moellendorffii* and *A*. *trichopoda* FLZ proteins was inferred by BI method based on JTT model. The numbers adjacent to the branches represent posteriori probability values. The tree is drawn to scale, with branch lengths measured in the number of amino acid substitutions per site.

In order to delineate the evolutionary history of individual *A*. *thaliana FLZ* genes, a phylogram of FLZ proteins was constructed along with members of *P*. *patens*, *S*. *moellendorffii* and *A*. *trichopoda* (Fig 4). As observed in the phylogram of *P*. *patens*, *S*. *moellendorffii*, *P*. *abies*, and *A*. *trichopoda*, the *P*. *patens* proteins were found in a distinct branch. Similarly, AtrFLZ3 was found in a distinct branch more close to *P*. *patens* proteins. The *S*. *moellendorffii* FLZ proteins were found in separate branches confirming the sequence divergence accumulated in Pteridophyta. The non-duplicated AthFLZ7 was close to SmoFLZ2 while SmoFLZ1 was found close to another non-duplicate AthFLZ15 and *A*. *trichopoda* members AtrFLZ6, AtrFLZ7, and AtrFLZ8. As observed in the earlier phylogram, AtrFLZ1 and AtrFLZ2 were found close in a distinct branch. The remaining two *A*. *trichopoda* proteins were found close to the duplicated *A*. *thaliana* FLZ proteins suggesting that these members are related to the ancestral *FLZ* genes which had undergone duplication in *A*. *thaliana*.

The gene structure, transcript forms and protein domain organization of *A*. *thaliana FLZ* gene family were studied to analyse the structural changes happened in the genes during the course of evolution (S5 Fig). The gene organization was found to be conserved in most cases. However, change in the length of exon, intron and UTRs was observed frequently. Except *AthFLZ12* and *AthFLZ16*, all other genes possess 5’ and 3’ UTRs and two exons. *AthFLZ12* lacks 5’UTR and *AthFLZ16* lack both UTRs and possess three exons which can be transcribed into two splice forms (S5A and S5B Fig). *AthFLZ8*, *AthFLZ10*, and *AthFLZ11* have intron in 5’ UTR. All *FLZ* genes including both splice forms of *AthFLZ16* has functional *FLZ* domain which was found to be situated towards C-terminal of the protein (S5C Fig).

Comparison of the gene structure of duplicated genes revealed structural divergence in few paralogous pairs (S5A Fig). The tandem duplicated *AthFLZ17* and *AthFLZ18* genes showed no difference in the structural organization. In case of block duplicated paralogs *AthFLZ1*, *AthFLZ2* and *AthFLZ3*, variation was observed in the length of UTRs, intron and exons while no variation in the structural organization was observed. The same feature was observed in the case of block duplicated *AthFLZ5-AthFLZ6* and *AthFLZ10-AthFLZ11* gene pairs. Along with variation in the length of gene components, divergence in the gene organization was observed in *AthFLZ8*-*AthFLZ9* and *AthFLZ12*-*AthFLZ13* block duplicated gene pairs. The changes in the exon length resulted in the divergence of protein length in many duplicated genes (S5C Fig).

### Phylogenetic analysis of *Oryza sativa FLZ* gene family

**Fig 5:**
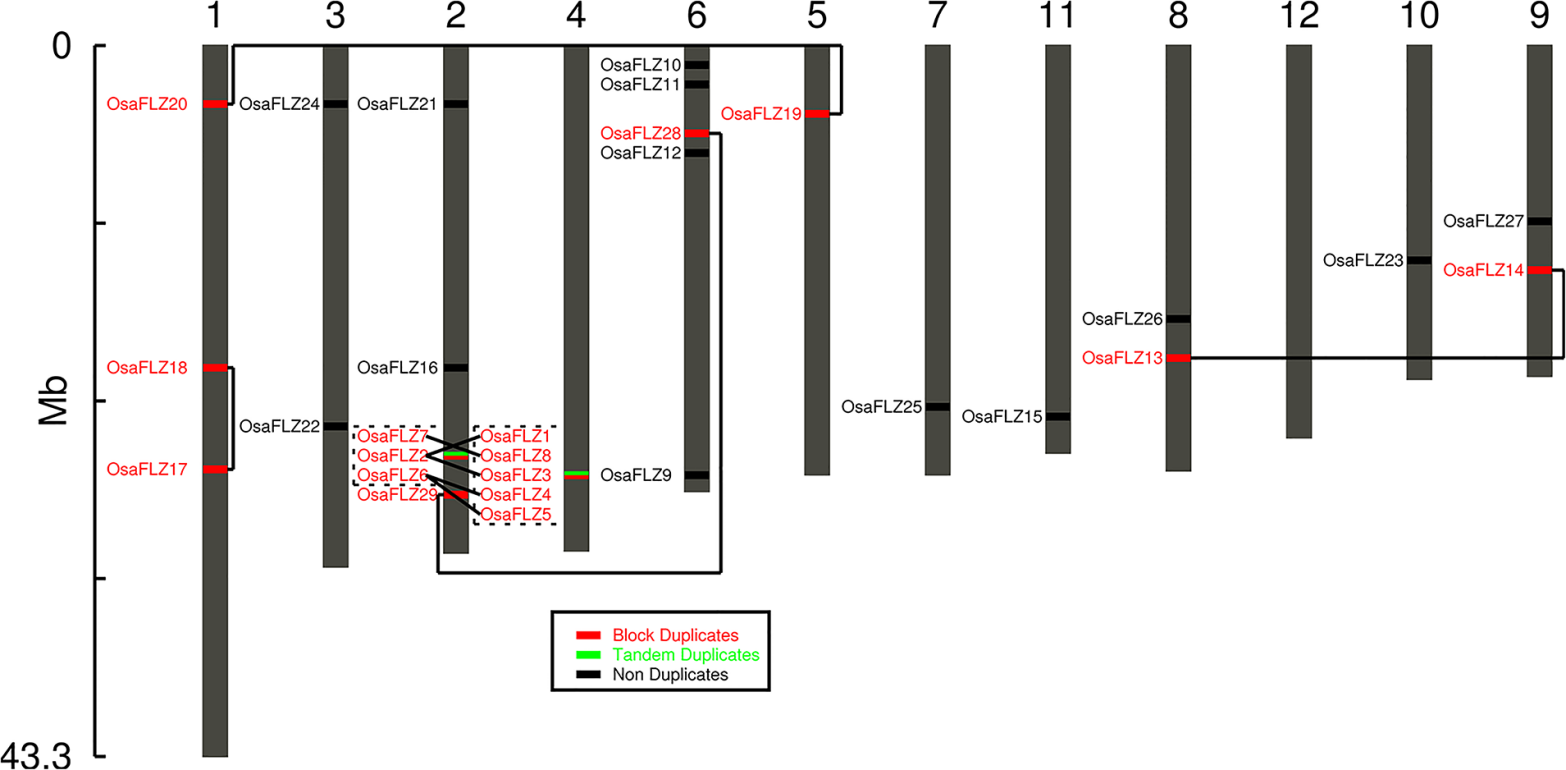
Chromosomal location and evolutionary history of *Oryza sativa FLZ* gene family. The gene duplication analysis is done using PLAZA v2.5and verified by the position of duplicates in phylogram. Solid line connects the block duplicates and dotted line connects the tandem duplicates. The numbers indicated at the top represents the chromosome number.

*O*. *sativa* genome contains 29 *FLZ* genes which were found to be distributed in all chromosomes except chromosome number 12 (Fig 5). The *O*. *sativa* homologue of *AthFLZ1* was named as *OsaFLZ1* and the remaining genes were named according to its closeness with this gene in the protein phylogram (S6 Fig). Due to segment-specific block duplication events, the chromosomal distribution of *FLZ* gene family in *O*. *sativa* was found to be more unequal than in the case of *A*. *thaliana*. Analysis of *O*. *sativa* genome identified a high representation of tandem repeats of genes [61]. Consistent with this general feature of the genome, more representation of tandem duplicated genes was found in *O*. *sativa FLZ* gene family compared to *A*. *thaliana*. However, the percentage of non-duplicated genes was found to be higher in *O*. *sativa* compared to *A*. *thaliana* (Table 1). The high incidence of block and tandem duplication happened in chromosomes 2 and 4 is one of the reasons behind the increased number of *FLZ* genes in *O*. *sativa*. As discussed earlier, the monocot specific σ and Poales specific ρWGD events also contributed to the increase of *FLZ* genes in grasses including rice [8, 9].

**Fig 6:**
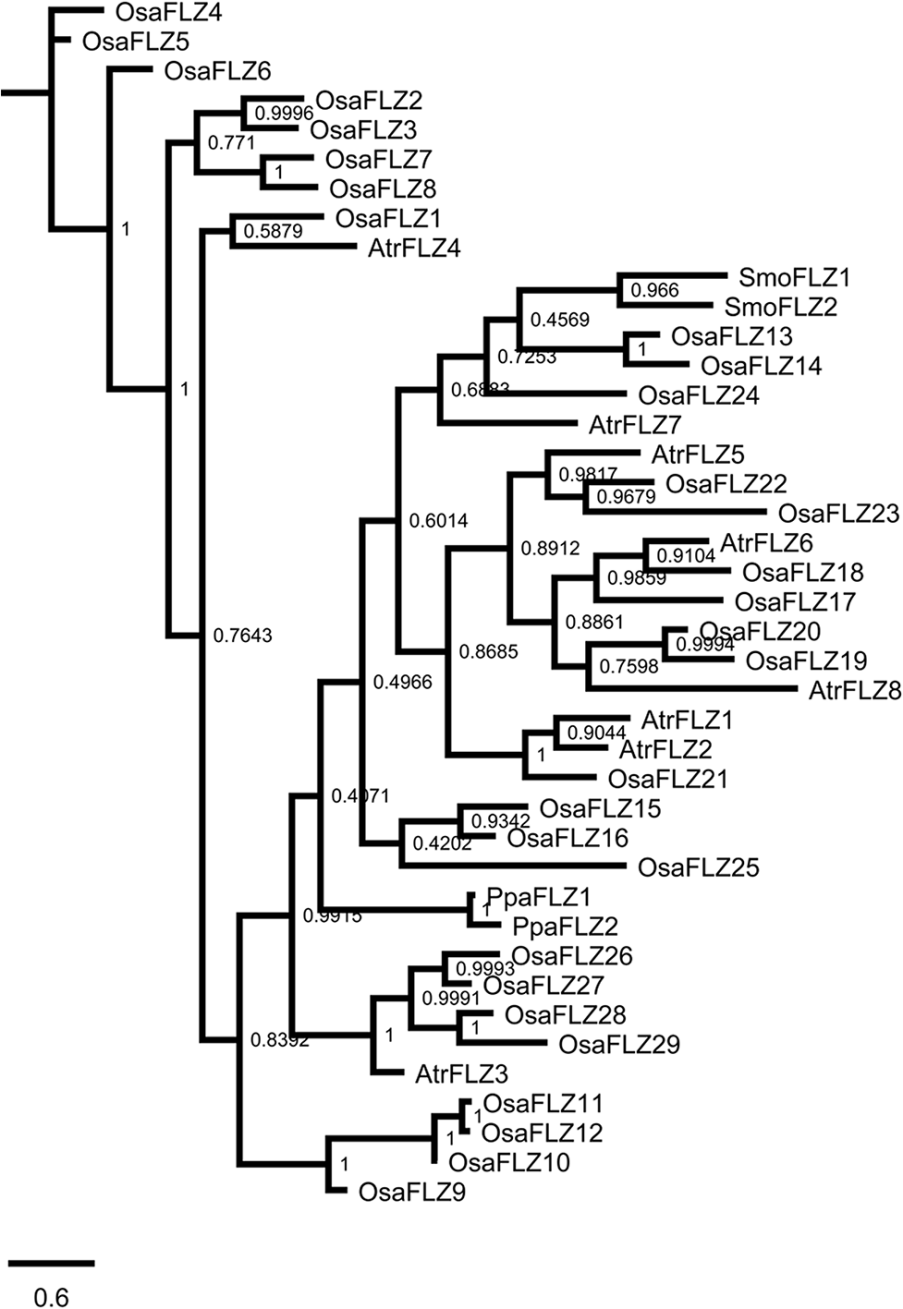
Analysis of phylogenetic origin of *Oryza sativa* FLZ proteins. Phylogram of *O*. *sativa*, *P*. *patens*, *S*. *moellendorffii* and *A*. *trichopoda* FLZ proteins is inferred by BI method based on JTT model. The numbers adjacent to the branches represent posteriori probability values. The tree is drawn to scale, with branch lengths measured in the number of amino substitutions per site.

A phylogram was constructed with *O*. *sativa*, *P*. *patens*, *S*. *moellendorffii*, and *A*. *trichopoda FLZ* gene family members to analyse the evolutionary history of *O*. *sativa* genes (Fig 6). The extensively duplicated genes in the chromosome 2 and 4 formed a distinct cluster with large branches. AtrFLZ4 was also found in this cluster suggesting that this gene is close to the ancestral gene which underwent duplication. Similarly, most of the *A*. *trichopoda* proteins were found in distinct clusters with *O*. *sativa* proteins suggesting their similarity with the ancestral genes in monocots which underwent duplication during the course of time.

Phylogenetic comparison of *O*. *sativa FLZ* gene family with *P*. *patens*, *S*. *moellendorffii*, and *A*. *trichopoda FLZ* gene families revealed the evolutionary history of *FLZ* genes in *O*. *sativa*. The phylogenetic analysis further confirms two hypotheses proposed earlier. Firstly, the Bryophyta-Pteridophyta divergence in the *FLZ* genes is also evident in the analysis of *O*. *sativa FLZ* family. Secondly, the rampant block duplications in the segment of chromosomes 2 and 4 are also evident in the phylogram as the duplicates were found in a cluster.

The gene structure, splice forms and protein domain organization of *O*. *sativa FLZ* gene family was studied to analyse the extent of structural divergence among members (S7 Fig). It was found that the gene organization of *O*. *sativa FLZ* gene family accumulated more diversity than in the case of *A*. *thaliana* (S7A Fig). Similarly, more frequency of splice variants was observed in *O*. *sativa FLZ* gene family (S7B Fig). The increase of gene structure diversity must be a contributing factor in the formation of different splice variants in *O*. *sativa FLZ* gene family. Consistent with this hypothesis, it was found that except *OsaFLZ27*, all other genes which produce splice variants accumulated more divergence from the general structural pattern observed in *FLZ* gene family. Ultimately, the increased divergence in the gene structure pattern must be contributing to the functional divergence of these genes in this species. As observed in the case of *A*. *thaliana*, most of the *O*. *sativa FLZ* genes possess two exons and 5’ and 3’ UTRs. One or both UTRs are missing in many genes. More frequency of genes with introns in UTR was also observed in *O*. *sativa FLZ* gene family which might have a role in gene expression regulation. Interestingly, few splice variants lack FLZ domain region or possess truncated FLZ domain (S7B and S7C Fig). *OsaFLZ25*.*1* and *OsaFLZ27*.*1* have functional FLZ domain while *OsaFLZ25*.*2* and *OsaFLZ27*.*2* lack FLZ domain completely. *OsaFLZ22*.*1* have functional FLZ domain while other two splice variants possess truncated FLZ domain with first two cysteine residues. It is already found that the FLZ domain with all four conserved cysteine residues intact is important for protein-protein interaction [22]. FLZ domain is the only conserved functional domain in *O*. *sativa FLZ* gene family. Taking this fact into account, the biological functions of splice variants without FLZ domain or truncated domain has to scrutinize further.

The gene structures of duplicated genes were compared to identify the structural divergence. Block duplicated pairs *OsaFLZ13*-*OsaFLZ14* and *OsaFLZ28*-*OsaFLZ29* have similar structural organization albeit little variation in the length of gene components (S7A Fig). High divergence in the gene structure was observed in the duplicated genes in the segment of chromosomes 2 and 4. *OsaFLZ1* displays the typical two exon pattern with both UTRs and a large intron. *OsaFLZ3* also have the same gene structure with a shorter intron while another paralog, *OsaFLZ2* have shorter intron but lack both UTRs (S7A Fig). Similarly, structural variation was also observed in duplicated genes *OsaFLZ4*, *OsaFLZ5*, and *OsaFLZ6*. *OsaFLZ4* possess a larger intron but lack both UTRs while *OsaFLZ5* was found to be intronless and carry both UTRs. *OsaFLZ6* shows typical *FLZ* gene pattern with a shorter intron. In block duplicated pair *OsaFLZ7* and *OsaFLZ8*, the latter has an intron in 3’UTR. *OsaFLZ18* found to have two introns in 5’UTR while its paralogue *OsaFLZ17* has only one intron in 5’UTR. Similarly, gain and loss of gene components was also observed in block duplicates *OsaFLZ19* and *OsaFLZ20*.

### Analysis of evolutionary pressure on *FLZ* gene family

**Fig 7:**
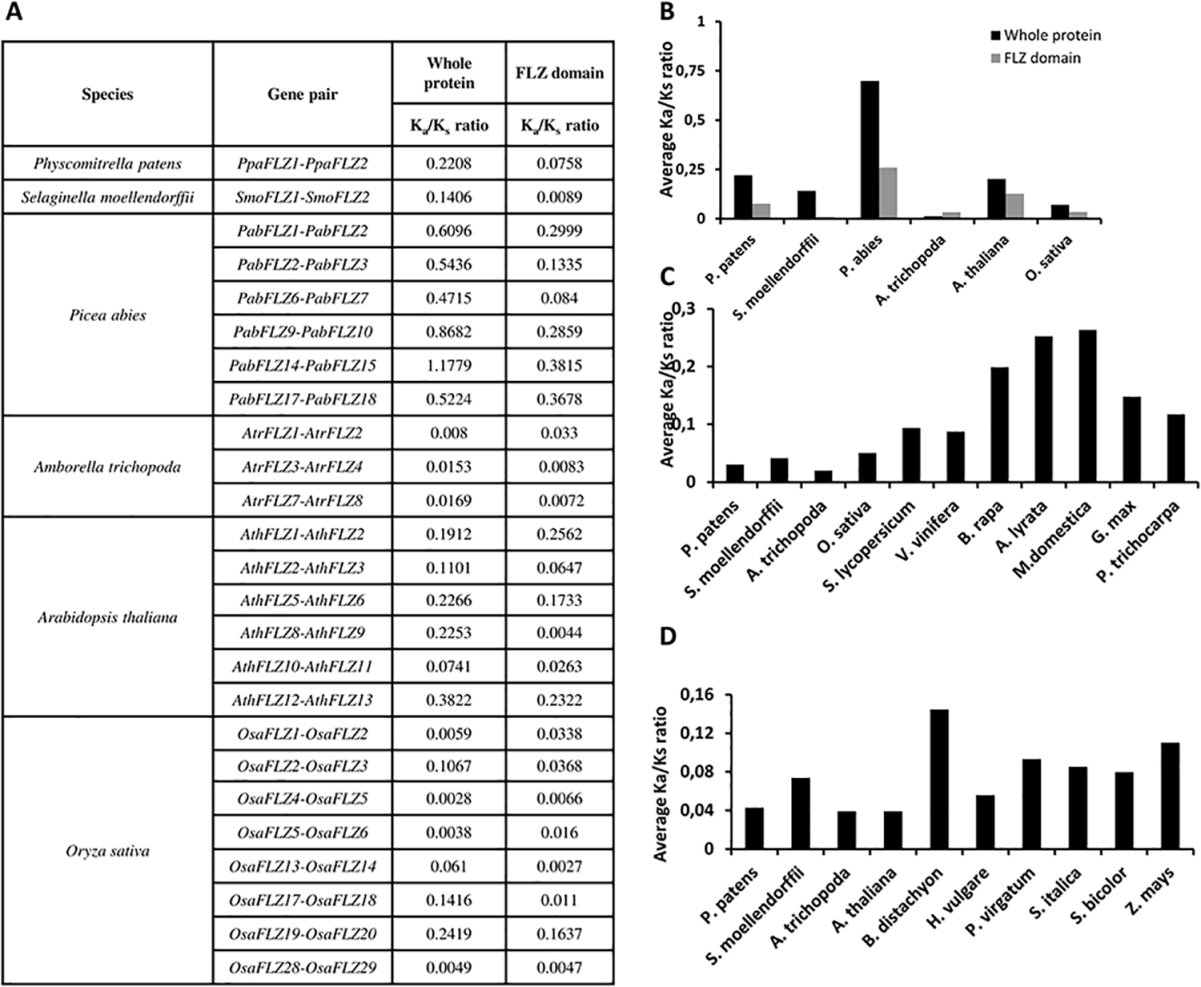
Analysis of evolutionary pressure on *FLZ* genes (A) Ka/Ks ratio of gene pair of *P*. *patens* and *S*. *moellendorffii*, *P*. *abies* and duplicated gene pairs of *A*. *trichopoda*, *A*. *thaliana* and *O*. *sativa*. The Ka/Ks ratio of whole gene and FLZ domain region is individually calculated. (B) averaged Ka/Ks ratio of whole gene and FLZ domain region of selected species studied. (C) and (D) averaged Ka/Ks ratio between *A*. *thaliana* (C) and *O*. *sativa* (D) genes and their orthologous genes from selected species. The Ka/Ks ratio of each pair is given in S2 and S3 Tables.

The duplicated genes undergo selection processes like hypofunctionalization, neofunctionalization, subfunctionalization and nonfunctionalization according to the evolutionary pressure. The survivors of the selection pressure undergo strong purifying selection [62]. The Ka/Ks ratio is a widely used method to analyse the selection pressure acting on protein-coding genes [63]. The Ka/Ks ratio of the *FLZ* gene pairs of *P*. *patens* and *S*. *moellendorffii*, putative duplicates of *P*. *abies* and *A*. *trichopoda* and duplicated gene pairs of *A*. *thaliana* and *O*. *sativa* were estimated (Fig 7A). Except in *P*. *abies*, the Ka/Ks ratios of FLZ genes were found to be very much less than1 indicating a strong purifying selection (Fig 7A and 7B). Although the Ka/Ks ratio was found to be less than 1 n all gene pairs of *P*. *abies* except in *PabFLZ14*-*PabFLZ15*, these gene pairs showed higher ratio value when compared to other species studied. The lower nucleotide substitution rate compared to angiosperms due to their comparatively long life span is a characteristic feature of conifer genome evolution [51, 64, 65]. This feature of conifers explains the increased Ka/Ks ratio of *FLZ* genes observed in *P*. *abies*. Further, the Ka/Ks ratios of orthologous *FLZ* gene pairs were also studied. Six genes from *A*. *thaliana* and *O*. *sativa* each were selected and Ka/Ks ratio with their orthologous genes from selected species were estimated (S2 and S3 Tables). The Ka/Ks ratios of orthologous gene pairs were also found to be lower than 1 confirming the strong purifying selection in *FLZ* gene family (Fig 7C and 7D).

It is an already established fact that the selection pressure acting on a gene is not uniform on the entire stretch. Different modules can be under different selection pressure creating a chimera of selection pressure on a single gene. In case of FLZ proteins, it is already found that the FLZ domain part is the most conserved module in the whole protein (S1 Fig). Taking all these information into account, we calculated the Ka/Ks ratio of domain region of paralogous *FLZ* gene pairs (Fig 7A). In general, a significant reduction in the Ka/Ks ratio was observed in FLZ domain region when compared to whole protein (Fig 7A and 7B). However, in some cases such as *AtrFLZ1-AtrFLZ2*, *AthFLZ1*-*AthFLZ2*, and *OsaFLZ1*-*OsaFLZ2*, the ratio was found higher for the domain region compared to whole protein. These results suggest that generally the FLZ domain region is under more purifying selection and in some cases whole protein was found to be under more strict purifying selection. In general, these results confirm that the evolutionary pressure on *FLZ* gene is not uniform on the entire stretch.

### Spatiotemporal expression analysis of *Arabidopsis thaliana FLZ* gene family

**Fig 8:**
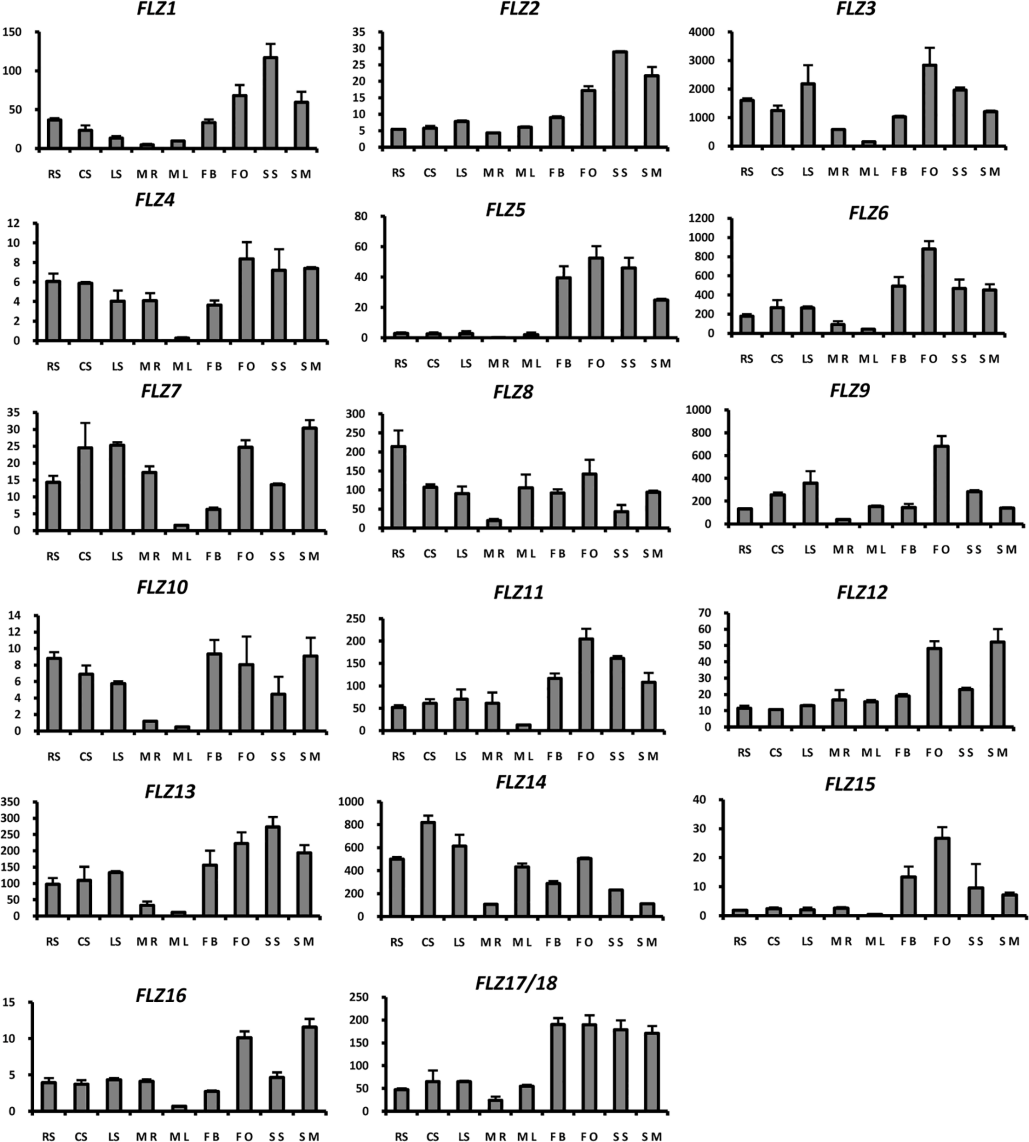
Expression analysis of *Arabidopsis thaliana FLZ* gene family in different developmental stages and tissues using real-time PCR. *UBQ10* is used as endogenous control. The absolute expression is calculated from ΔCT value. Graphs represent average and error bars represent mean ± SD of two biological replicates with three technical replicates each. X axis represent samples and Y axis represent absolute expression value. Samples are abbreviated in graph is as follows, RS, radicle emerged; CS, cotyledon stage seedling; LS, 2 leaves stage seedling; MR, mature plant root; ML, mature bolts rosette leaf; FB, flower bud; FO, flower open; SS, silique small; SM, silique mature.

Understanding the spatiotemporal expression dynamics of genes can give clues about their function. Microarray-based expression analysis of *A*. *thaliana FLZ* gene family revealed that *FLZ* gene family have a varied expression pattern. Many of the *FLZ* gene family members found to have induced expression in reproductive organs and silique [22]. However, a detailed developmental- and organ-specific expression study of *FLZ* gene family is lacking. In this study, we analysed the expression pattern of 18 *A*. *thaliana FLZ* genes in different developmental stages and organs using qRT-PCR (Fig 8).

Most of the *FLZ* genes showed increased expression in flower and/or silique stages suggesting a specific function in the development of these organs. *AthFLZ1* expressed high in developing seed, flower etc compared to seedling stages and vegetative organs. Among the paralogous genes of *AthFLZ1*, *AthFLZ2* showed similar expression pattern while *AthFLZ3* showed induced expression in flower, silique and seedling stages suggesting divergence in its expression domain. The block duplicated pair *AthFLZ5* and *AthFLZ6* showed overlapping expression domain with a small amount of divergence. *AthFLZ7* was found to be profusely expressed in seedling stages, root and flower and mature silique. Similarly, *AthFLZ9* also showed high induction of expression in open flower compared to flower bud. *AthFLZ8* was found to be highly expressed in developing seedlings. *AthFLZ10* showed a uniform expression pattern in all stages studied except root and leaf of mature rosette where it showed highly reduced expression. The homologous *AthFLZ11* gene showed almost similar expression pattern with a fair degree of divergence. Another block duplicated pair, *AthFLZ12* and *AthFLZ13* showed high divergence in expression domain. *AthFLZ12* was expressed uniformly in all tissues studied except open flower and mature silique where it showed high expression compared to other tissues. However, the homologue *AthFLZ13* showed higher expression in both flower and silique stages studied. Besides, *AthFLZ13* also showed reduced expression in root and leaf of bolted plant. Unlike the majority of other *FLZ* genes, *AthFLZ14* showed higher expression in seedling stages compared to reproductive organs. *AthFLZ15* showed specific expression in reproductive organs. The expression of *AthFLZ16* was highly up-regulated in open flower and mature silique. The tandem duplicated pair *AthFLZ17/18* showed almost uniform expression pattern in vegetative stages while expression was found to be highly up-regulated in reproductive stages.

Gene expression analysis from publically available microarray data revealed that *A*. *thaliana FLZ* gene family is highly responsive to hormonal and nutritional cues. Most of the *A*. *thaliana FLZ* genes are up-regulated in response to glucose treatment [22]. Consistent with this, the interaction of FLZ proteins with RAPTOR1B, SnRK1.1 and SnRK1.2 confirms the involvement of *FLZ* gene family in sugar and energy signaling [22, 27]. The role of sugars in promoting vegetative-to-reproductive phase transition and promoting flowering is well documented [66–69]. Sugars, through HEXOKINASE1-dependent signaling, repress microRNA156 at transcriptional and post-transcriptional levels which release the inhibition of key regulators of juvenile-to-adult and vegetative-to-reproductive phase transition like SQUAMOSA PROMOTER BINDING PROTEIN-LIKE transcriptional factors [66, 67].

**Fig 9:**
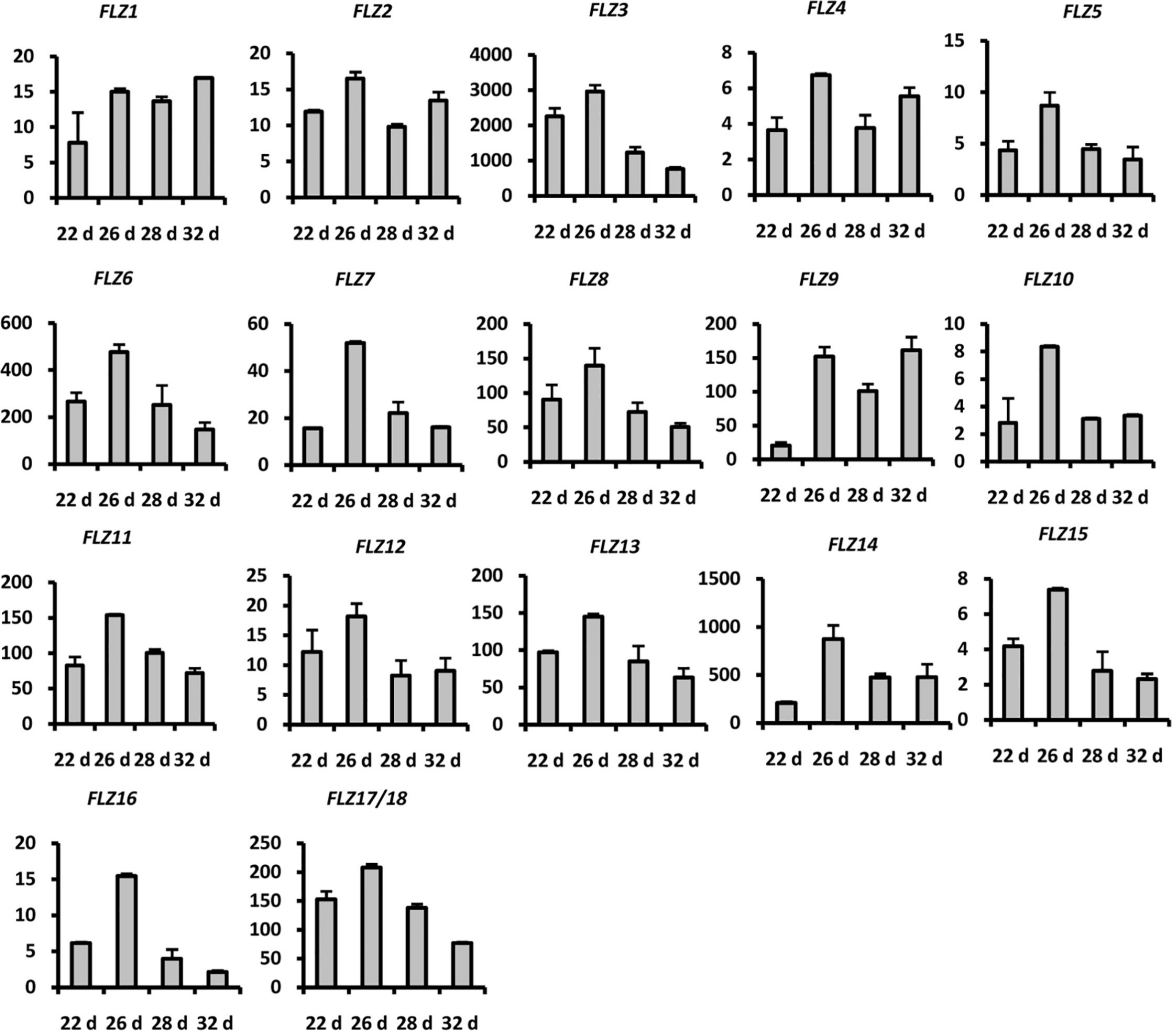
Expression analysis of *Arabidopsis thaliana FLZ* gene family during phase transition and bolting using real-time PCR. *UBQ10* is used as endogenous control. The absolute expression is calculated from ΔCT value. Graphs represent average and error bars represent mean ± SD of two biological replicates. X axis represent samples and Y axis represent absolute expression value. Samples are abbreviated in graph is as follows, 22d, rosette 22 day old; 26d, rosette 26 day old; 28d, rosette 28 day old; 32d, bolted rosette 32d day old.

As the role of *FLZ* genes in sugar and energy signaling is emerging, we tried to explore the possibility of involvement of *FLZ* gene in vegetative-to-reproductive phase transition through analysis of expression dynamics of these genes during the period. Four rosette growth stages based on growth stage-based phenotypic analysis of Boyes et al. and according to visible phenotype were selected for the analysis of expression pattern of *FLZ* genes [47]. At 22 days and 26 days, the rosette attained approximately 50% and 70% of final rosette size respectively without any visible flower bud appearance. The rosette attained full size at 28 days with appearance of flower buds, but no bolting. At 32 days, the plants displayed 1 cm length bolt with prominent flower bud appearance. The expression of all 18 *FLZ* genes in these four developmental stages was analysed by qRT-PCR (Fig 9). Interestingly, all genes showed fluctuations in expression in the stages studied. In many genes, compared to 22 days old rosette, the expression was up-regulated in 26 days old rosette which was the prerequisite stage of flower bud appearance. Different expression dynamics were observed after the induction of expression from 22 days to 26 days. The increased expression in 26 days old was found to be gradually declined in other stages in most of the genes. In the case of *AthFLZ1*, the increased expression was more or less retained in other stages. *AthFLZ9* showed decline of expression in 28 days while the expression was got induced in 32 days. This preliminary analysis gives clues about the role of *FLZ* gene in vegetative-to-reproductive phase transition. Considering the role of sugar in phase transition and the recent integration of *FLZ* gene family in sugar and energy signaling, this observation invites an extensive molecular dissection.

Analysis of expression pattern of *A*. *thaliana FLZ* gene family revealed that the expression of the majority of *FLZ* genes is induced in reproductive organs compared to vegetative stages and organs. This organ-specific induction of *FLZ* gene family suggests a possible role of this gene family in controlling reproductive development. It was also found that most of the *FLZ* genes display a similar expression dynamics during the vegetative-to-reproductive phase transition. The common expression domains displayed by even diverged *FLZ* genes suggest the possibility of a high degree of functional conservation in this gene family. As it is observed in the case of many multigene families, the strong purifying selection observed in the *FLZ* gene family must be responsible for the functional redundancy. Consistent with this hypothesis, functional redundancy aspect of *FLZ* gene family is also revealed during protein-protein interaction studies. It is already found that FLZ protein share many common interacting partners such as RAPTOR1B, SnRK1.1, SnRK1.2 which are conserved regulators of plant growth [27]. In a detailed protein-protein interaction study with FLZ proteins, it was identified that all 18 *A*. *thaliana* FLZ can interact with SnRK1.1 and SnRK1.2 which are the kinase subunits of SnRK1 [22]. Both SnRK1 and TOR are multi-subunit protein kinases and the interaction of many FLZ proteins with subunits of these kinases suggest the possibility that may be FLZ proteins also form complex with these kinases in response to different cues. The common expression domain shared by many of the *FLZ* genes might be facilitating this complex formation during different stages of plant development.

### Analysis of expression pattern divergence of duplicated *Arabidopsis thaliana FLZ* genes

Large-scale expression analysis of *A*. *thaliana* and *O*. *sativa* duplicated genes identified that in many cases paralogs underwent divergence in the expression domain [70–72]. Analysis of expression pattern divergence in duplicated genes serves as an excellent tool to study the functional divergence in the gene family. From the qRT-PCR based expression data of *FLZ* genes, the expression profile of 5 block duplicated gene pairs were analysed for expression pattern divergence (S8 Fig). Comparison of expression pattern of *AthFLZ1* and *AthFLZ2* identified a high degree of expression retention in this block duplicated pair. *AthFLZ1* and *AthFLZ2* displayed comparatively shorter branch length in the protein phylogram of *A*. *thaliana* (Fig 4). Comparison of expression profile of *AthFLZ5* and *AthFLZ6* identified divergence in expression domain. The expression of *AthFLZ5* was found to be restricted to the reproductive organs while there was a significant amount of *AthFLZ6* expression in seedling stages also. No divergence in expression was observed during vegetative-to-reproductive phase transition stages. A high degree of expression divergence was observed in the expression pattern of *AthFLZ8* and *AthFLZ9* in seedling stages, reproductive organs and vegetative-to-reproductive phase transition stages. *AthFLZ8* and *AthFLZ9* also displayed a very high degree of sequence divergence in the protein phylogram (Fig 4). A fair degree of expression divergence was also observed in the *AthFLZ10*-*AthFLZ11* and *AthFLZ12*-*AthFLZ13* block duplicated gene pairs.

The analysis of expression divergence among block duplicated *A*. *thaliana FLZ* genes revealed branch specific variation in the degree of expression divergence. *AthFLZ1*-*AthFLZ2* gene pair showed a high degree of expression retention. Similarly, many of other gene pairs also showed expression retention in many developmental stages and tissues studied. This indicates the possible functional redundancy among duplicated genes. The strong purifying selection observed in this gene family must be a contributing to the functional redundancy of duplicated genes. As observed in the case of *AthFLZ8*-*AthFLZ9* gene pair, few genes displayed unique and sometimes contrasting expression pattern compared to its paralogous gene. This can be because of gain or loss of expression domains after duplication. A positive correlation between expression divergence and sequence and/or structural divergence among duplicated genes is observed in many species such as yeast, human, and *Caenorhabditis elegans* [73]. However, in the case of *A*. *thaliana*, different groups identified positive, negative and zero correlations [70, 71, 73, 74] which suggest that the correlation between sequence and expression divergence can be varied in different duplicated genes. In *FLZ* gene family, the duplicated gene pairs with high sequence divergence showed a high degree of expression divergence while the pairs with less sequence divergence showed comparatively less degree of expression divergence. This observation suggests that the correlation between sequence and expression divergence in the duplicated genes in *A*. *thaliana FLZ* gene family is most likely positive.

## Conclusions

The present study gives a comprehensive account on the evolution of *FLZ* gene family and the expression dynamics of *A*. *thaliana FLZ* gene family in different developmental stages and tissues. The FLZ domain was found to be highly conserved across the plant kingdom. Apart from FLZ domain, MEME search identified novel motifs in FLZ proteins which must be contributing to the functional divergence of this family. Comparison of WGD events happened in plant lineage and *FLZ* gene family size revealed that general- and lineage specific WGD events and subsequent gene loss etc contributed in the evolutionary expansion of this family. Genomic duplication events also contributed to the expansion of *FLZ* gene family in many cases. Detailed analysis of *FLZ* gene family of *A*. *thaliana* and *O*. *sativa* revealed monocot and dicot specific variations in the evolution of this gene family. The *FLZ* genes were found to under high purifying selection. The expression analysis of *A*. *thaliana FLZ* gene family suggests their role in vegetative development, vegetative-to-reproductive phase transition and reproductive development. The common interacting proteins identified before and common expression domains revealed in the study imply functional redundancy in *FLZ* gene family. This study will be helpful in uncovering the functions played by the *FLZ* gene family in plant growth and development which is not yet completely understood.

## Supporting Information

S1 Fig Sequence conservation in FLZ domain proteins. (PPTX)

S2 Fig Phylogram of *Amborella trichopoda* and *Picea abies* FLZ proteins. (PPTX)

S3 Fig Species phylogenetic tree showing *FLZ* gene family distribution and WGD events. (PPTX)

S4 Fig Exon-intron structure and protein domain organization of *FLZ* family genes from *Physcomitrella patens*, *Selaginella moellendorffii*, *Picea abies* and *Amborella trichopoda*. (PPTX)

S5 Fig Gene structure, splice variants and domain organization of *Arabidopsis thaliana FLZ* family. (PPTX)

S6 Fig Phylogram of *Oryza sativa* FLZ proteins. (PPTX)

S7 Fig Gene structure, splice variants and domain organization of *Oryza sativa FLZ* family. (PPTX)

S8 Fig Expression pattern of duplicated *Arabidopsis FLZ* genes. (PPTX)

S1 Table Nomenclature of *FLZ* genes used in this study. (DOCX)

S2 Table Ka/Ks ratio of *Arabidopsis thaliana FLZ* genes and orthologous genes from selected species. (DOCX)

S3 Table Ka/Ks ratio of *Oryza sativa FLZ* genes and orthologous genes from selected species. (DOCX)

S4 Table Primers used for qRT-PCR. (DOCX)

S5 Table List of FLZ proteins identified from different genomes in this study. (DOCX)

S6 Table Novel motifs identified from FLZ proteins using MEME. (DOCX)

S7 Table Homology detection of novel motifs identified in MEME analysis. (DOCX)
