# Supplementary material for: Comprehensive Evolutionary and Expression Analysis of FCS-Like Zinc finger Gene Family Yields Insights into Their Origin, Expansion and Divergence
Source: PLoS One. 2015 Aug 7;10(8):e0134328. doi: 10.1371/journal.pone.0134328 (PMC4529292; doi:10.1371/journal.pone.0134328)
Supplement: S1 Fig — (PPTX) [file pone.0134328.s001.pptx]

## Slide 1
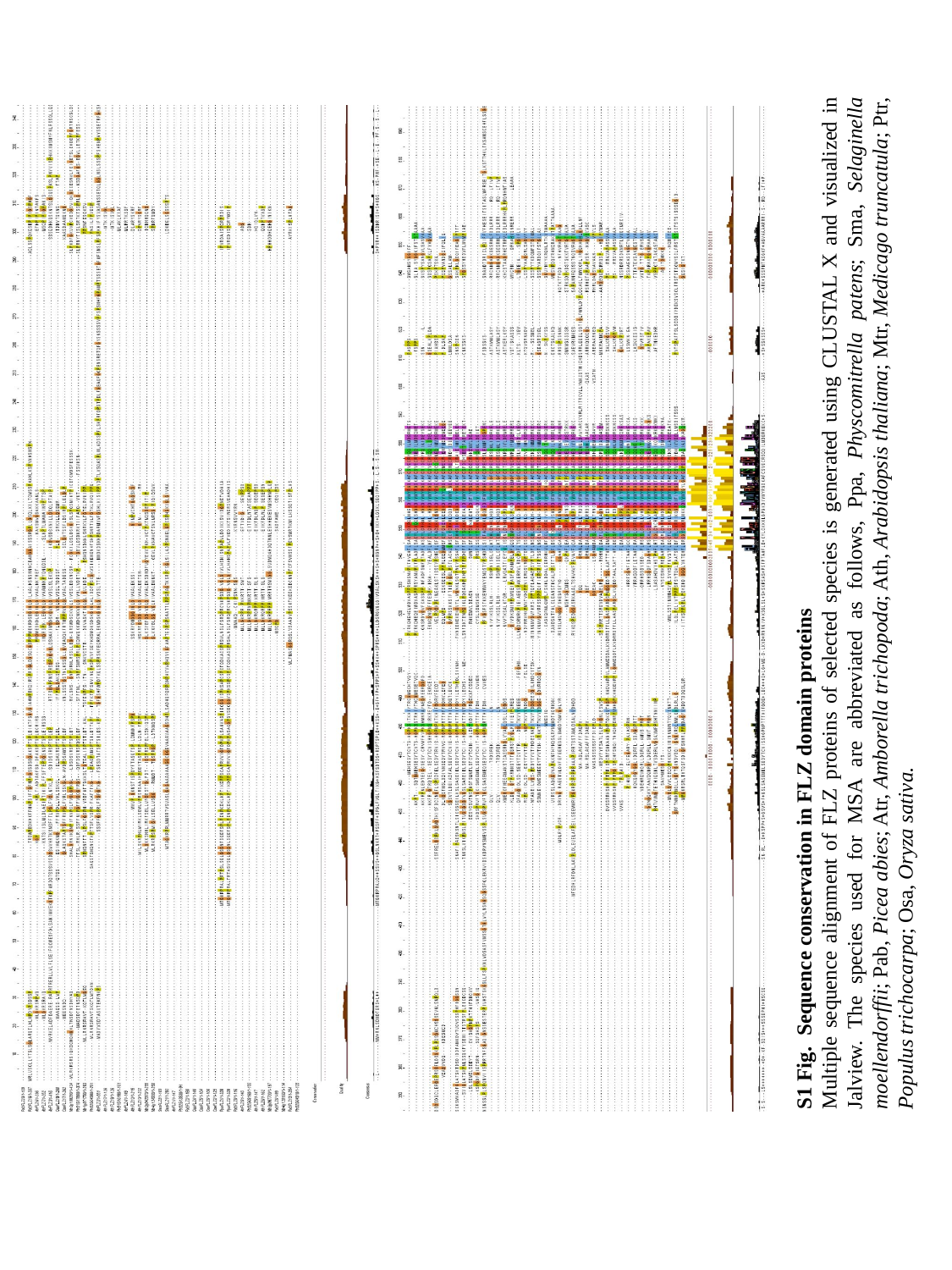

S1 Fig. Sequence conservation in FLZ domain proteins
Multiple sequence alignment of FLZ proteins of selected species is generated using CLUSTAL X and visualized in Jalview. The species used for MSA are abbreviated as follows, Ppa, Physcomitrella patens; Sma, Selaginella moellendorffii; Pab, Picea abies; Atr, Amborella trichopoda; Ath, Arabidopsis thaliana; Mtr, Medicago truncatula; Ptr, Populus trichocarpa; Osa, Oryza sativa.
