# Supplementary material for: Comprehensive Evolutionary and Expression Analysis of FCS-Like Zinc finger Gene Family Yields Insights into Their Origin, Expansion and Divergence
Source: PLoS One. 2015 Aug 7;10(8):e0134328. doi: 10.1371/journal.pone.0134328 (PMC4529292; doi:10.1371/journal.pone.0134328)
Supplement: S2 Fig — (PPTX) [file pone.0134328.s002.pptx]

## Slide 1
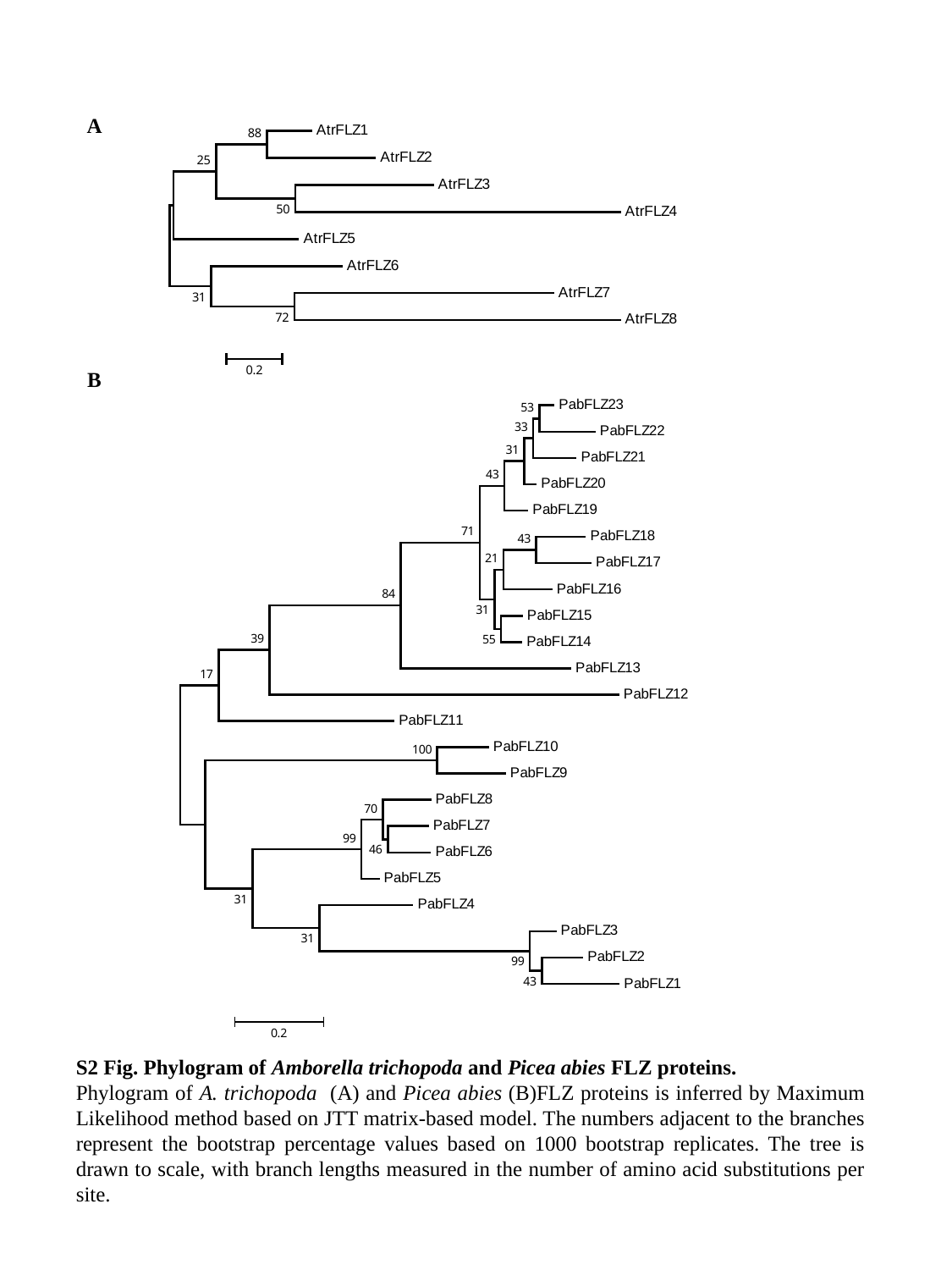

A
B
S2 Fig. Phylogram of Amborella trichopoda and Picea abies FLZ proteins.
Phylogram of A. trichopoda (A) and Picea abies (B)FLZ proteins is inferred by Maximum Likelihood method based on JTT matrix-based model. The numbers adjacent to the branches represent the bootstrap percentage values based on 1000 bootstrap replicates. The tree is drawn to scale, with branch lengths measured in the number of amino acid substitutions per site.
