# Supplementary material for: Comprehensive Evolutionary and Expression Analysis of FCS-Like Zinc finger Gene Family Yields Insights into Their Origin, Expansion and Divergence
Source: PLoS One. 2015 Aug 7;10(8):e0134328. doi: 10.1371/journal.pone.0134328 (PMC4529292; doi:10.1371/journal.pone.0134328)
Supplement: S3 Fig — (PPTX) [file pone.0134328.s003.pptx]

## Slide 1
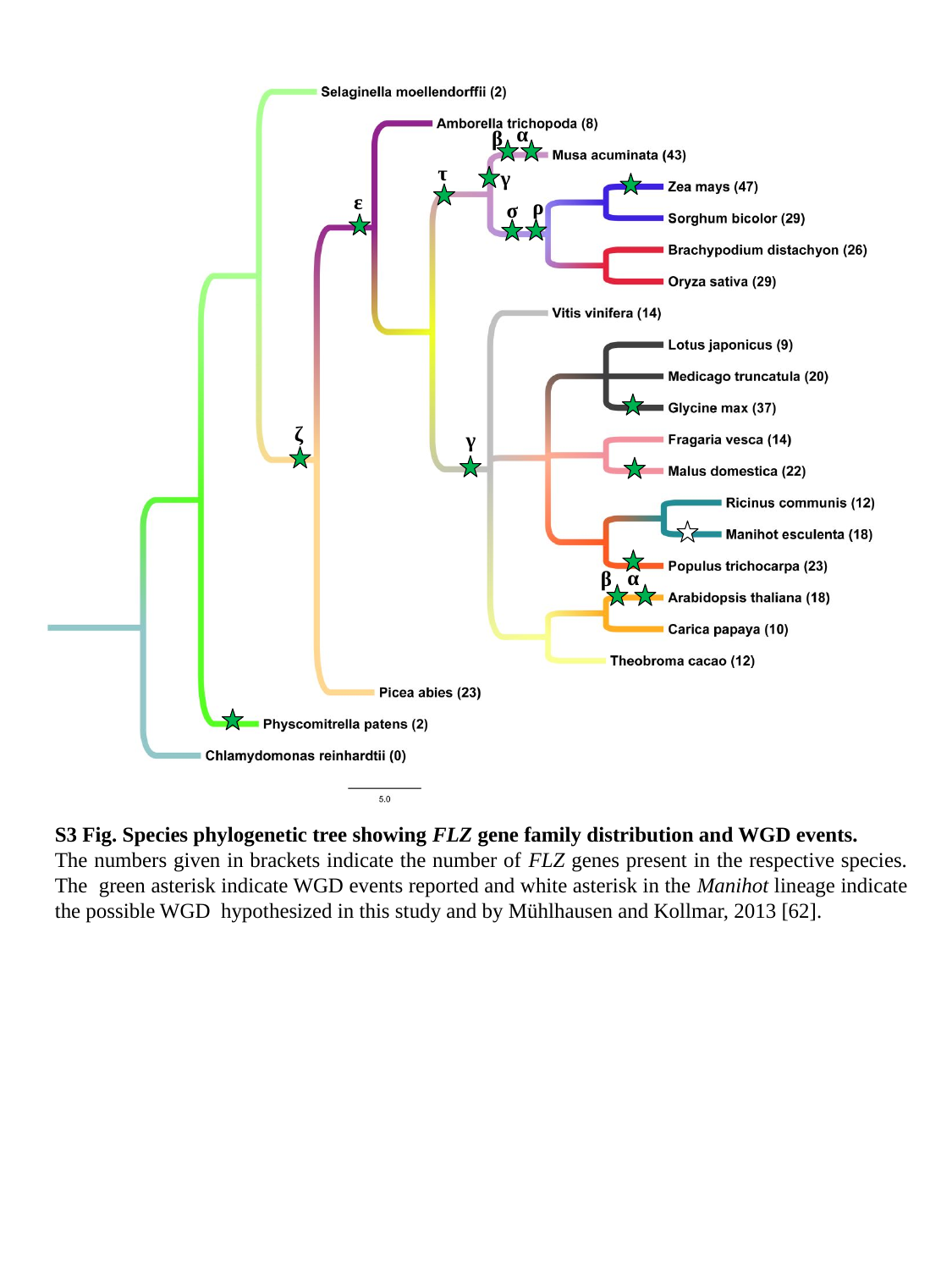

α
β
τ
γ
ε
ρ
σ
ζ
γ
α
β
S3 Fig. Species phylogenetic tree showing FLZ gene family distribution and WGD events.
The numbers given in brackets indicate the number of FLZ genes present in the respective species. The green asterisk indicate WGD events reported and white asterisk in the Manihot lineage indicate the possible WGD hypothesized in this study and by Mühlhausen and Kollmar, 2013 [62].
