# Supplementary material for: Comprehensive Evolutionary and Expression Analysis of FCS-Like Zinc finger Gene Family Yields Insights into Their Origin, Expansion and Divergence
Source: PLoS One. 2015 Aug 7;10(8):e0134328. doi: 10.1371/journal.pone.0134328 (PMC4529292; doi:10.1371/journal.pone.0134328)
Supplement: S4 Fig — (PPTX) [file pone.0134328.s004.pptx]

## Slide 1
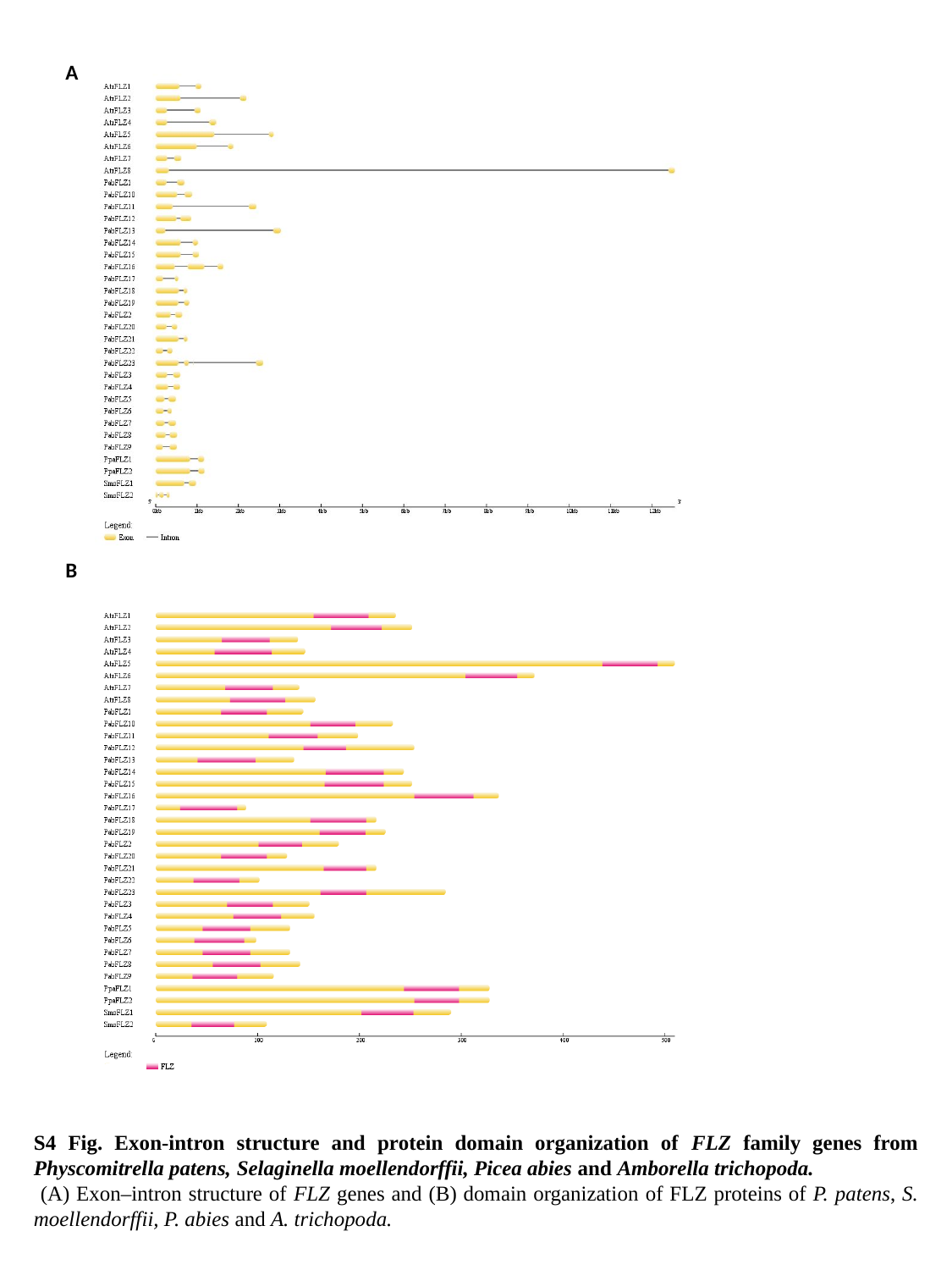

A
B
S4 Fig. Exon-intron structure and protein domain organization of FLZ family genes from Physcomitrella patens, Selaginella moellendorffii, Picea abies and Amborella trichopoda.
 (A) Exon–intron structure of FLZ genes and (B) domain organization of FLZ proteins of P. patens, S. moellendorffii, P. abies and A. trichopoda.
