# Supplementary material for: Comprehensive Evolutionary and Expression Analysis of FCS-Like Zinc finger Gene Family Yields Insights into Their Origin, Expansion and Divergence
Source: PLoS One. 2015 Aug 7;10(8):e0134328. doi: 10.1371/journal.pone.0134328 (PMC4529292; doi:10.1371/journal.pone.0134328)
Supplement: S6 Fig — (PPTX) [file pone.0134328.s006.pptx]

## Slide 1
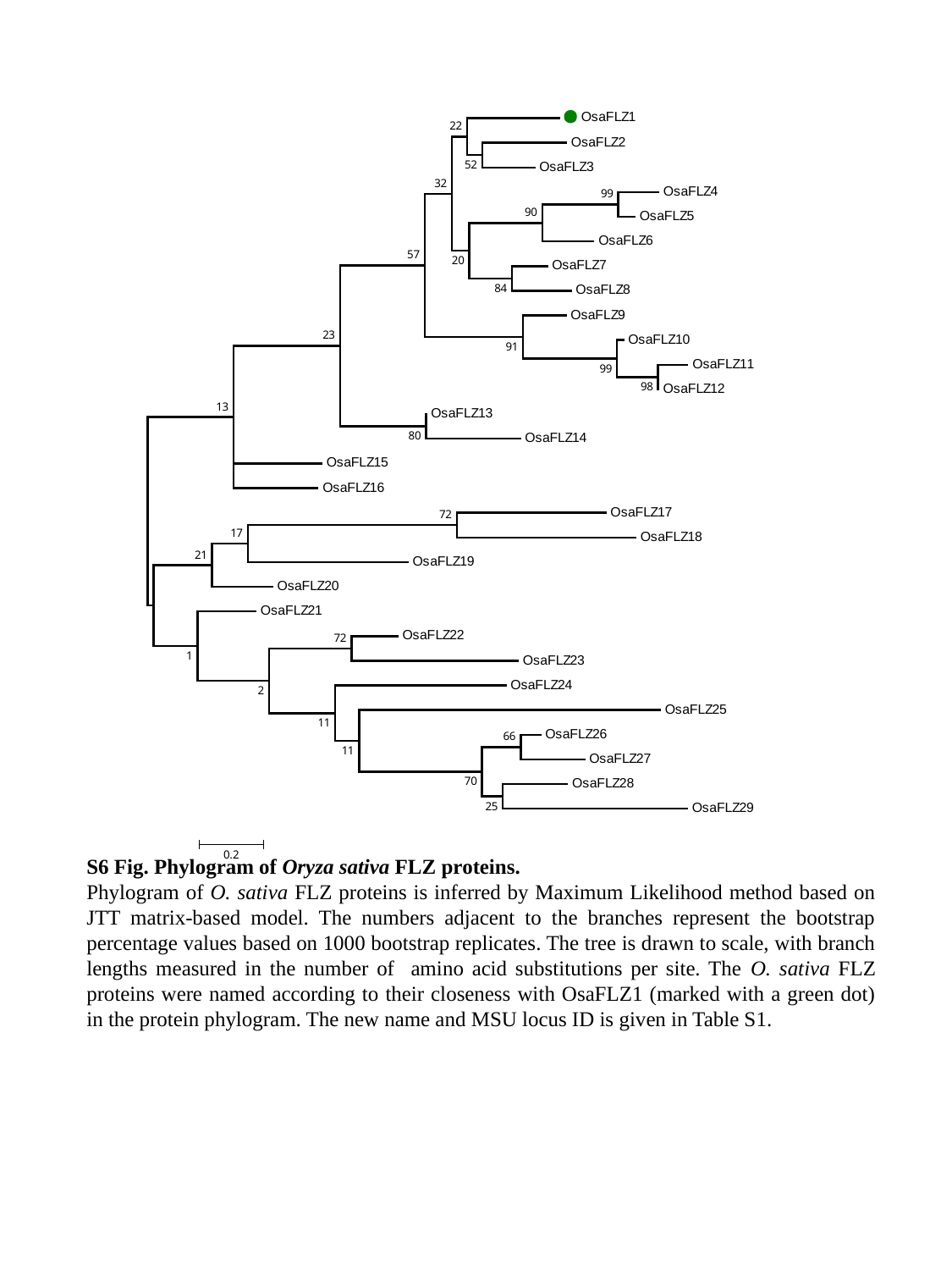

S6 Fig. Phylogram of Oryza sativa FLZ proteins.
Phylogram of O. sativa FLZ proteins is inferred by Maximum Likelihood method based on JTT matrix-based model. The numbers adjacent to the branches represent the bootstrap percentage values based on 1000 bootstrap replicates. The tree is drawn to scale, with branch lengths measured in the number of amino acid substitutions per site. The O. sativa FLZ proteins were named according to their closeness with OsaFLZ1 (marked with a green dot) in the protein phylogram. The new name and MSU locus ID is given in Table S1.
