# Supplementary material for: Comprehensive Evolutionary and Expression Analysis of FCS-Like Zinc finger Gene Family Yields Insights into Their Origin, Expansion and Divergence
Source: PLoS One. 2015 Aug 7;10(8):e0134328. doi: 10.1371/journal.pone.0134328 (PMC4529292; doi:10.1371/journal.pone.0134328)
Supplement: S7 Fig — (PPTX) [file pone.0134328.s007.pptx]

## Slide 1
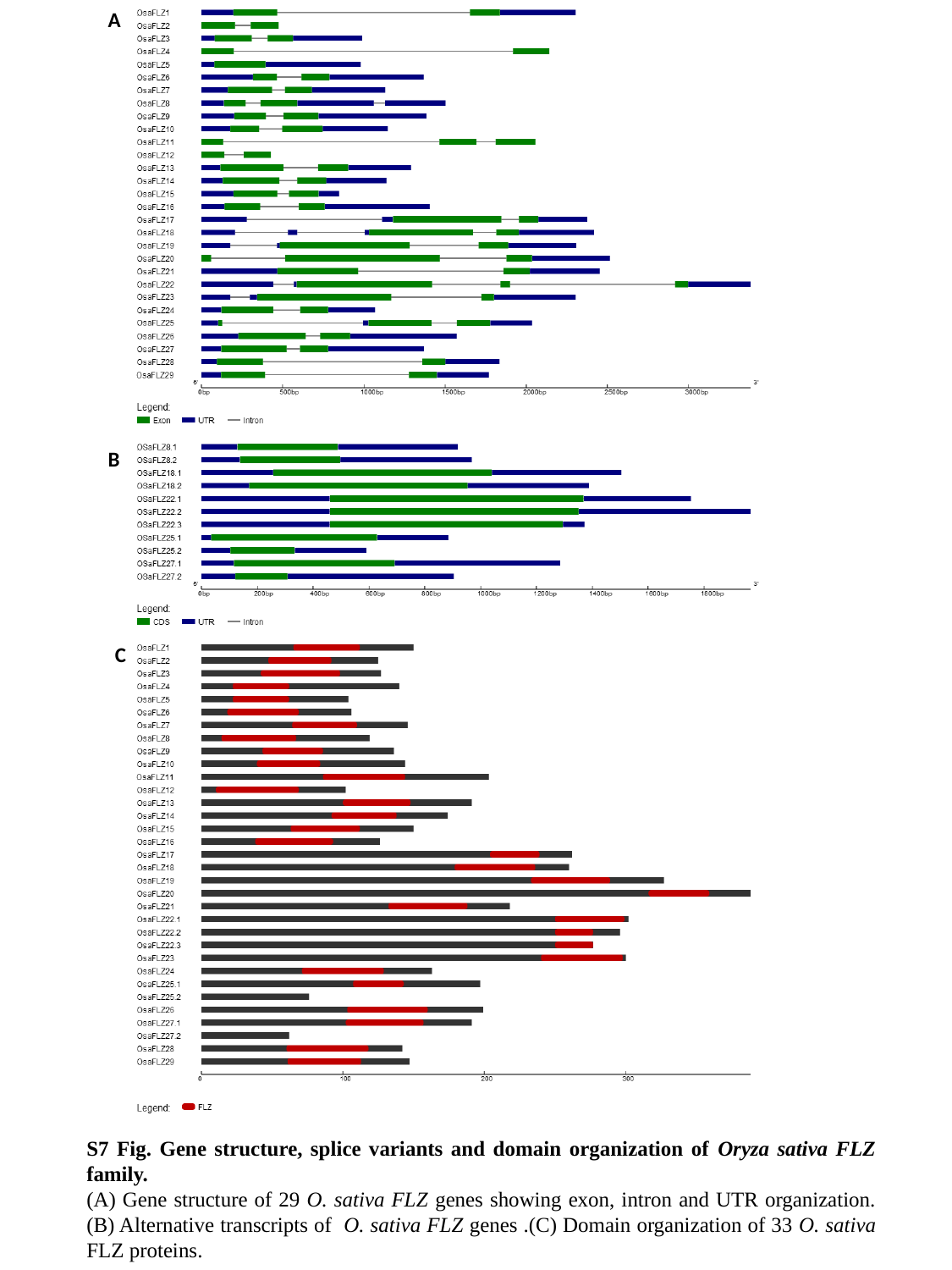

A
B
C
S7 Fig. Gene structure, splice variants and domain organization of Oryza sativa FLZ family.
(A) Gene structure of 29 O. sativa FLZ genes showing exon, intron and UTR organization. (B) Alternative transcripts of O. sativa FLZ genes .(C) Domain organization of 33 O. sativa FLZ proteins.
