# Supplementary material for: Comprehensive Evolutionary and Expression Analysis of FCS-Like Zinc finger Gene Family Yields Insights into Their Origin, Expansion and Divergence
Source: PLoS One. 2015 Aug 7;10(8):e0134328. doi: 10.1371/journal.pone.0134328 (PMC4529292; doi:10.1371/journal.pone.0134328)
Supplement: S8 Fig — (PPTX) [file pone.0134328.s008.pptx]

## Slide 1
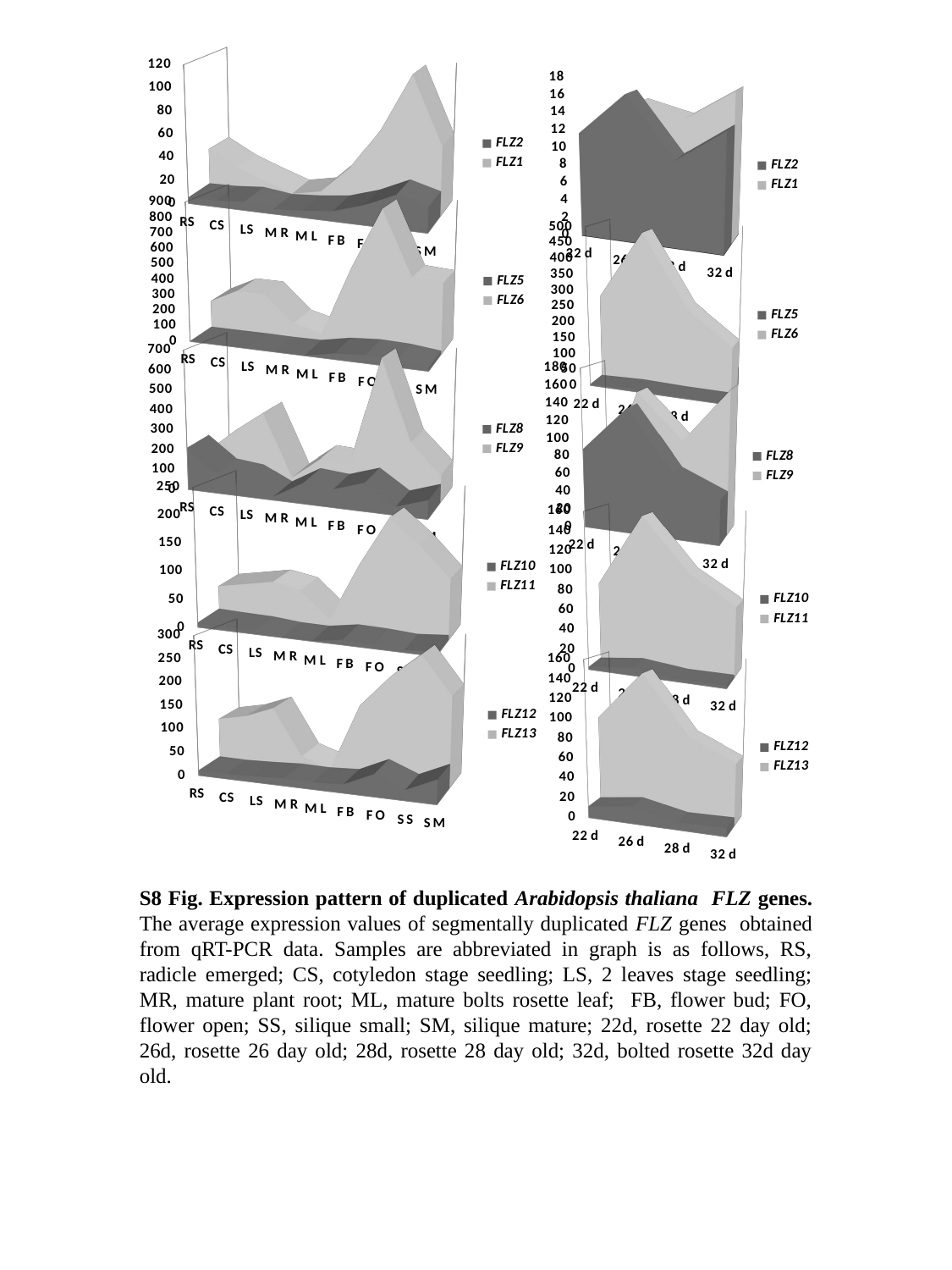

[unsupported chart]
[unsupported chart]
[unsupported chart]
[unsupported chart]
[unsupported chart]
[unsupported chart]
[unsupported chart]
[unsupported chart]
[unsupported chart]
[unsupported chart]
S8 Fig. Expression pattern of duplicated Arabidopsis thaliana FLZ genes. The average expression values of segmentally duplicated FLZ genes obtained from qRT-PCR data. Samples are abbreviated in graph is as follows, RS, radicle emerged; CS, cotyledon stage seedling; LS, 2 leaves stage seedling; MR, mature plant root; ML, mature bolts rosette leaf; FB, flower bud; FO, flower open; SS, silique small; SM, silique mature; 22d, rosette 22 day old; 26d, rosette 26 day old; 28d, rosette 28 day old; 32d, bolted rosette 32d day old.
