# Supplementary material for: Comprehensive Evolutionary and Expression Analysis of FCS-Like Zinc finger Gene Family Yields Insights into Their Origin, Expansion and Divergence
Source: PLoS One. 2015 Aug 7;10(8):e0134328. doi: 10.1371/journal.pone.0134328 (PMC4529292; doi:10.1371/journal.pone.0134328)
Supplement: S1 Table — (DOCX) [file pone.0134328.s009.docx]

| **S1 Table. Nomenclature of *FLZ* genes used in this study** | | |
| --- | --- | --- |
| **Species** | **Gene name** | **Accession number** |
| *Physcomitrella patens* | *PpaFLZ1* | Pp1s194_166V6.1 |
|  | *PpaFLZ2* | Pp1s395_10V6.1 |
| *Selaginella moellendorffii* | *SmoFLZ1* | estExt_fgenesh2_pg.C_1210063 |
|  | *SmoFLZ2* | fgenesh2_pg.C_scaffold_113000031 |
| *Amborella trichopoda* | *AtrFLZ1* | LOC18433773 |
|  | *AtrFLZ2* | LOC18440768 |
|  | *AtrFLZ3* | LOC18421995 |
|  | *AtrFLZ4* | LOC18443777 |
|  | *AtrFLZ5* | LOC18421374 |
|  | *AtrFLZ6* | LOC18434212 |
|  | *AtrFLZ7* | LOC18423993 |
|  | *AtrFLZ8* | LOC18429661 |
| *Picea abies* | *PabFLZ1* | MA_40616g0010 |
|  | *PabFLZ2* | MA_14341g0010 |
|  | *PabFLZ3* | MA_18295g0020 |
|  | *PabFLZ4* | MA_88917g0010 |
|  | *PabFLZ5* | MA_124336g0010 |
|  | *PabFLZ6* | MA_7041008g0010 |
|  | *PabFLZ7* | MA_2360g0010 |
|  | *PabFLZ8* | MA_6300g0010 |
|  | *PabFLZ9* | MA_237265g0010 |
|  | *PabFLZ10* | MA_90279g0010 |
|  | *PabFLZ11* | MA_10431633g0010 |
|  | *PabFLZ12* | MA_17351g0010 |
|  | *PabFLZ13* | MA_103900g0010 |
|  | *PabFLZ14* | MA_66837g0010 |
|  | *PabFLZ15* | MA_481693g0010 |
|  | *PabFLZ16* | MA_10432609g0010 |
|  | *PabFLZ17* | MA_101286g0010 |
|  | *PabFLZ18* | MA_9563380g0010 |
|  | *PabFLZ19* | MA_844505g0010 |
|  | *PabFLZ20* | MA_75487g0010 |
|  | *PabFLZ21* | MA_3394g0020 |
|  | *PabFLZ22* | MA_255510g0010 |
|  | *PabFLZ23* | MA_95738g0010 |
| *Arabidopsis thaliana* | *AthFLZ1* | At5g47060 |
|  | *AthFLZ2* | At4g17670 |
|  | *AthFLZ3* | At2g44670 |
|  | *AthFLZ4* | At5g65040 |
|  | *AthFLZ5* | At1g22160 |
|  | *AthFLZ6* | At1g78020 |
|  | *AthFLZ7* | At4g39795 |
|  | *AthFLZ8* | At3g22550 |
|  | *AthFLZ9* | At3g63210 |
|  | *AthFLZ10* | At5g11460 |
|  | *AthFLZ11* | At2g25690 |
|  | *AthFLZ12* | At1g19200 |
|  | *AthFLZ13* | At1g74940 |
|  | *AthFLZ14* | At5g20700 |
|  | *AthFLZ15* | At5g49120 |
|  | *AthFLZ16* | At3g63230 |
|  | *AthFLZ17* | At1g53885 |
|  | *AthFLZ18* | At1g53903 |
| *Oryza sativa* | *OsaFLZ1* | LOC_Os04g49620 |
|  | *OsaFLZ2* | LOC_Os02g46190 |
|  | *OsaFLZ3* | LOC_Os04g49660 |
|  | *OsaFLZ4* | LOC_Os04g49670 |
|  | *OsaFLZ5* | LOC_Os04g49680 |
|  | *OsaFLZ6* | LOC_Os02g46210 |
|  | *OsaFLZ7* | LOC_Os02g46180 |
|  | *OsaFLZ8* | LOC_Os04g49650 |
|  | *OsaFLZ9* | LOC_Os06g50080 |
|  | *OsaFLZ10* | LOC_Os06g03520 |
|  | *OsaFLZ11* | LOC_Os06g05970 |
|  | *OsaFLZ12* | LOC_Os06g14070 |
|  | *OsaFLZ13* | LOC_Os08g34984 |
|  | *OsaFLZ14* | LOC_Os09g26370 |
|  | *OsaFLZ15* | LOC_Os11g43790 |
|  | *OsaFLZ16* | LOC_Os02g37970 |
|  | *OsaFLZ17* | LOC_Os01g52100 |
|  | *OsaFLZ18* | LOC_Os01g41010 |
|  | *OsaFLZ19* | LOC_Os05g08800 |
|  | *OsaFLZ20* | LOC_Os01g08520 |
|  | *OsaFLZ21* | LOC_Os02g07820 |
|  | *OsaFLZ22* | LOC_Os03g46260 |
|  | *OsaFLZ23* | LOC_Os10g28680 |
|  | *OsaFLZ24* | LOC_Os03g08520 |
|  | *OsaFLZ25* | LOC_Os07g42390 |
|  | *OsaFLZ26* | LOC_Os08g31510 |
|  | *OsaFLZ27* | LOC_Os09g20240 |
|  | *OsaFLZ28* | LOC_Os06g11980 |
|  | *OsaFLZ29* | LOC_Os02g51550 |
