# Supplementary material for: Comprehensive Evolutionary and Expression Analysis of FCS-Like Zinc finger Gene Family Yields Insights into Their Origin, Expansion and Divergence
Source: PLoS One. 2015 Aug 7;10(8):e0134328. doi: 10.1371/journal.pone.0134328 (PMC4529292; doi:10.1371/journal.pone.0134328)
Supplement: S2 Table — (DOCX) [file pone.0134328.s010.docx]

| ***A. thaliana*** | ***P. patens*** | ***S. moellendorffii*** | ***A. trichopoda*** | ***O. sativa*** | ***S. lycopersicum*** | ***V. vinifera*** | ***B. rapa*** | ***A. lyrata*** | ***M. domestica*** | ***G. max*** | ***P. trichocarpa*** |  |
| --- | --- | --- | --- | --- | --- | --- | --- | --- | --- | --- | --- | --- |
| **AthFLZ1** | PpaFLZ2 | SmoFLZ1 | AtrFLZ4 | OsaFLZ3 | Solyc06g061010 | LOC100257499 | Bra024966 | 494274 | MDP0000203930 | Glyma01g41170 | Potri.003G085700 | Orthologous gene |
|  | 0.0911 | 0.0886 | 0.0255 | 0.1064 | 0.0808 | 0.0046 | 0.1733 | 0.2307 | 0.0854 | 0.1097 | 0.1513 |  |
| **AthFLZ5** | PpaFLZ2 | SmoFLZ1 | AtrFLZ3 | OsaFLZ26 | Solyc04g054760 | LOC100252409 | Bra031369 | 472449 | MDP0000154807 | Glyma04g11460 | Potri.002G092900 |  |
|  | 0.0114 | 0.0109 | 0.0098 | 0.0905 | 0.2679 | 0.17 | 0.243 | 0.2501 | 0.2128 | 0.1357 | 0.1525 |  |
| **AthFLZ9** | PpaFLZ2 | SmoFLZ1 | AtrFLZ7 | OsaFLZ22 | Solyc09g082210 | LOC100855273 | Bra040437 | 324745 | MDP0000188194 | Glyma19g34060 | Potri.005G211500 |  |
|  | 0.0205 | 0.061 | 0.0294 | 0.0125 | 0.0995 | 0.1391 | 0.2286 | 0.1445 | 0.1297 | 0.1039 | 0.1627 |  |
| **AthFLZ14** | PpaFLZ2 | SmoFLZ1 | AtrFLZ6 | OsaFLZ21 | Solyc07g042190 | LOC100264520 | Bra002322 | 941626 | MDP0000871412 | Glyma17g05520 | Potri.006G139200 | Ka/  Ks  ratio |
|  | 0.0231 | 0.0231 | 0.0146 | 0.0202 | 0.0668 | 0.188 | 0.1066 | 0.1462 | 0.3099 | 0.24 | 0.0983 |  |
| **AthFLZ16** | PpaFLZ1 | SmoFLZ1 | - | OsaFLZ9 | Solyc08g077480 | LOC100853829 | Bra040438 | 349378 | MDP0000196004 | - | Potri.002G050100 |  |
|  | 0.0215 | 0.0508 | - | 0.0192 | 0.0303 | 0.0145 | 0.2519 | 0.378 | 0.699 | - | 0.0095 |  |
| **AthFLZ18** | PpaFLZ2 | SmoFLZ1 | - | OsaFLZ14 | Solyc03g123460 | LOC100253160 | Bra039700 | 923953 | MDP0000233392 | Glyma16g01550 | Potri.001G163400 |  |
|  | 0.0146 | 0.0165 | - | 0.0547 | 0.0159 | 0.0068 | 0.1897 | 0.3662 | 0.1453 | 0.1509 | 0.1294 |  |

**S2 Table. Ka/Ks ratio of *Arabidopsis thaliana* *FLZ* genes and orthologous genes from selected species**
