# Supplementary material for: Comprehensive Evolutionary and Expression Analysis of FCS-Like Zinc finger Gene Family Yields Insights into Their Origin, Expansion and Divergence
Source: PLoS One. 2015 Aug 7;10(8):e0134328. doi: 10.1371/journal.pone.0134328 (PMC4529292; doi:10.1371/journal.pone.0134328)
Supplement: S3 Table — (DOCX) [file pone.0134328.s011.docx]

**S3 Table. Ka/Ks ratio of *Oryza sativa* *FLZ* genes and orthologous genes from selected species**

| ***O. sativa*** | ***P. patens*** | ***S. moellendorffii*** | ***A. trichopoda*** | ***A. thaliana*** | ***B. distachyon*** | ***H. vulgare*** | ***P. virgatum*** | ***S. italica*** | ***S. bicolor*** | ***Z. mays*** |  |
| --- | --- | --- | --- | --- | --- | --- | --- | --- | --- | --- | --- |
| **OsaFLZ1** | PpaFLZ2 | SmoFLZ1 | AtrFLZ4 | AthFLZ1 | Bradi5g19600 | BAK06059.1 | Pavirv00058314m | Si012978m | Sb06g026630 | LOC100273313 | Orthologous gene |
|  | 0.015 | 0.0169 | 0.0148 | 0.0164 | 0.0737 | 0.0048 | 0.074 | 0.1285 | 0.0437 | 0.144 |  |
| **OsaFLZ13** | PPaFLZ1 | SmoFLZ1 | AtrFLZ4 | AthFLZ5 | Bradi3g37390 | BAJ86234.1 | Pavirv00067803m | Si014510m | Sb07g022140 | LOC100275491 |  |
|  | 0.0739 | 0.1175 | 0.016 | 0.0183 | 0.1281 | 0.0121 | 0.1774 | 0.1264 | 0.2482 | 0.2072 |  |
| **OsaFLZ15** | PPaFLZ1 | SmoFLZ1 | AtrFLZ7 | AthFLZ15 | Bradi3g47530 | BAK03036.1 | Pavirv00035027m | Si020094m | Sb07g022140 | LOC100277951 |  |
|  | 0.0163 | 0.0721 | 0.0962 | 0.0539 | 0.0893 | 0.0715 | 0.0232 | 0.0045 | 0.0101 | 0.0245 |  |
| **OsaFLZ17** | PpaFLZ2 | SmoFLZ2 | AtrFLZ2 | AthFLZ8 | Bradi2g48330 | BAK00497.1 | Pavirv00045030m | Si002736m | Sb03g033030 | LOC100274019 | Ka/  Ks  ratio |
|  | 0.0215 | 0.0099 | 0.0168 | 0.0142 | 0.3698 | 0.1165 | 0.1313 | 0.13 | 0.0355 | 0.0361 |  |
| **OsaFLZ21** | PpaFLZ2 | SmoFLZ1 | AtrFLZ2 | AthFLZ13 | Bradi3g05560 | BAJ84784.1 | Pavirv00020836m | Si018332m | Sb04g005000 | LOC103626680 |  |
|  | 0.1169 | 0.2094 | 0.082 | 0.1161 | 0.1784 | 0.1218 | 0.1494 | 0.1174 | 0.1321 | 0.1161 |  |
| **OsaFLZ29** | PpaFLZ2 | SmoFLZ2 | AtrFLZ3 | AthFLZ6 | Bradi3g59070 | BAJ86070.1 | Pavirv00033070m | Si019201m | Sb10g007830 | LOC100274617 |  |
|  | 0.0131 | 0.0159 | 0.0088 | 0.0148 | 0.0293 | 0.0079 | 0.0044 | 0.0042 | 0.0076 | 0.1329 |  |
