# Supplementary material for: Comprehensive Evolutionary and Expression Analysis of FCS-Like Zinc finger Gene Family Yields Insights into Their Origin, Expansion and Divergence
Source: PLoS One. 2015 Aug 7;10(8):e0134328. doi: 10.1371/journal.pone.0134328 (PMC4529292; doi:10.1371/journal.pone.0134328)
Supplement: S4 Table — (DOCX) [file pone.0134328.s012.docx]

| **S4 Table. Primers used for qRT-PCR** | |
| --- | --- |
| *FLZ1F* | GAAGCTGGGTTTTCTGGTAACAA |
| *FLZ1 R* | TAAGAGAAACGGCTTGAAGAAACG |
| *FLZ2F* | GAAGACGATGGTTTTGTTTCTTTATCT |
| *FLZ2 R* | TCTGAGGATAGTTGTAACAAGAAGGACTT |
| *FLZ3F* | GTATTACTCTGGTTTTTTGGGTTGTG |
| *FLZ3 R* | TCCGGCAAAGAGAACACGAT |
| *FLZ4F* | CCCACGATCTATGACGCATCT |
| *FLZ4 R* | TGTGAGGGTGTTGATGAAAGGT |
| *FLZ5F* | CGGAACCACCACAGGAGAGA |
| *FLZ5 R* | TCACAGTCAATAGTGCTCAATGCTT |
| *FLZ6F* | TGTAACAGCAGCCGTTGATCA |
| *FLZ6 R* | ACCATGGATAAGAGCCGTTGA |
| *FLZ7F* | CGCAGCGTTTTGTAGCATAGAAT |
| *FLZ7 R* | GACGCGGTTTCTTGTTTTCC |
| *FLZ8F* | CCAGAATCATCTCCGGCTATTTC |
| *FLZ8 R* | CGCATGTGTAATCCTCCGATAAC |
| *FLZ9F* | TCGAGCCAACTATCCCTAATCC |
| *FLZ9 R* | GAGATCGGCTAGGCCAAAGA |
| *FLZ10F* | TCCGTCGATGCTGCTTACTG |
| *FLZ10 R* | GACAAGCATTGTTGTTCACACTGA |
| *FLZ11F* | TGTGGCCCTGCTGCTAAAG |
| *FLZ11 R* | CCGATCATCGCCAAGAGAA |
| *FLZ12F* | CCCGTCGCCTGGAAGTT |
| *FLZ12 R* | AAGCGCAGCGACGATACCTA |
| *FLZ13F* | CGAAGCGGTTCGGTTCTG |
| *FLZ13 R* | AGTTTCCTCCAACGCAGCAA |
| *FLZ14F* | AAATGATGAAAGGAAAGAGAGATGTAGA |
| *FLZ14 R* | TTTGGCCGGCGGTGTA |
| *FLZ15F* | TGCGCCGTCGCGTTA |
| *FLZ15 R* | AGCGAAACCGCCTGCTT |
| *FLZ16F* | GGAAGAACATCCAAAGATGAGG |
| *FLZ16 R* | GATGATGGTGATGATGAGATCCTA |
| *FLZ17/18F* | GAAGATGATGTTGGCTTCGTACAG |
| *FLZ17/18 R* | ACAAATTCCGAGACTCGCTTTT |
