# Supplementary material for: Comprehensive Evolutionary and Expression Analysis of FCS-Like Zinc finger Gene Family Yields Insights into Their Origin, Expansion and Divergence
Source: PLoS One. 2015 Aug 7;10(8):e0134328. doi: 10.1371/journal.pone.0134328 (PMC4529292; doi:10.1371/journal.pone.0134328)
Supplement: S5 Table — (DOCX) [file pone.0134328.s013.docx]

| **S5 Table. List of FLZ proteins identified from different genomes in this study** |
| --- |
| ***Amborella trichopoda*** |
| >LOC18443777  MDLRYPVCRDRYNGRAEETVLDVFEFEERESSLEMEDTQMVGHLYRRSRSLHSNGGFSHESRSFYDGGAHFLQACHLCNKPLGNGRDIFMYRGDTPFCSEECREEQMAKDEAMENRRRSSPSSNNKKQTKTTRDDNFHVRAGTVAAG  >LOC18421995  MLLGKRPRPVKRTTSMTEFTVDIGSEVPPSDADGPIHVPELFEGARYPAIASPRNHRRFSGNFIETAHFLQCCTLCKRRLGPGRDIYMYRGDSAFCSLECRQQQMNIDERKEKCALSSMKNEAPSSAAEASSNGETVAAA  >LOC18421374  MLRKRSRAVSSKPNLMADYVSDTASVTEKFNKPSSSSFFGSPRLFTGFGTKGFSESDTATSPTSILDSKPFTGFGSHFWPDTKQLSSPRSNFEQRRALEKMDSKGIGLGIVDSLTTENNDKDSKKPDARMVVFGSHLKISIPPYDPTLVSQASPLNLADSSPFLSKPRFDPSTPDLFGEAQFGCPENSRETQFGYSNSSSSVPFSPSNHFGPANPTSSSETGFMFSNSLPFSPKHIFYSVASANSSETQLGGLNSLSSSPFSHENPAYSSETRFGKSNSNSSLPLSPQNVIISPRRTNFSGAQIGNSSESTERPIVHSTSKGSLLFSPKNLVDSASFVNSSGTGLVYLNSNGSQGSSFQLEKRVIDSKDRVNSMEVSSPLSVGSPRVFTGCLSASEMEQSEDYTCIISHGPNSRTTHIFDNCVVESPFVGSFSTKKENWAVYENQSTYPSHDFLSICFACKKKLGQGEDIFMYRGEKAFCSRECRSLEMMFDEGPENCFSDSSVSSNAGV  >LOC18434212  MVEKGTKPFMRDQTSSSVSSDGSVVHRTKSNSFFTLPGLFVGLNTKLPSDIDSIRSPTSPLEPRTFSGNPFWSPRSGPDGHGLQSHAKIWGDSKGVGLGIVDSLSLEKDPIKGVSDSKNILLGSQLRTAIPISSSQDHKQSKNTSLGSQSPKSLPNYVISPHKKIMSNYFNLRSSQLLQDSGSDQCLERGEPRSGFLDSGKPLPQSGMCHSGSEVNLSNPQLDSSFRELGSPKLGMGTKISFDCCNGFLSPSDFELSEDYTRVISHGPNPKTKHIFGDRVFECDTVELPDGKPEIWEAKLSTISSSYPSNEILSFCYACKKKLGNGKDIYIYRGEKAFCSCQCRNEQIMVDERMEKEPIAHSSPPSKPTSKA  >LOC18433773  MLGKRSRPVQKGPNPLTIADTAKFSDDFAKSPTSPLDCRIRSPRGWESKDLEGIGLGIVAALNKTRETKAKSMVGSAKKAANLSETHPIPINTAKFSAKIRPNLEDTEIGCSESYCRVMFHVPKSHMNRAYNEGREERGGFDQNSRDYGVFCASSPPNFADFSMFPTADFLKSCYLCRKMLHGKDIYMYRGDKAFCSIDCRYRQIAIDEYSEKCSSNLSVSPYSANGVLSASVAAA  >LOC18440768  MLGKRSRAIQKNTSIISLMLDLAEIPTIKIPLFFSFTEPDGSNSPTSPLEIRSSSINGRSPKLEGVGLGIVAAMNKPYQNQDSLSLKPTLMANAINRPTTSPMSQPIPIGPAKISAKFRGTRQTELSESYTCVISHHGPNSIRKREYFDEKRCEIAEKNCGNCGFFFESPKKHAYEALDFLKFCYLCRKKLHGRDIYMYRGDKAFCSVECRCQQILSDELKEKCGSEALKPLDASDSPCSAPKLFYAGVAAA  >LOC18423993  MLRRTKSSFKLDGGTPKRNNSTTTGSRSTPVGLRIVIEIPQRESGIVDKSMIRPKMSEIPASFPANMPELGFLQSCSMCKKKLSPKNDIYIYRGEMGFCSVECRWRQIFMDERRERSVSTISKASSSMYCRNDRKAHVSNRERRLEAAA  >LOC18429661  MRFKNKKLQPYAMDSNSSFFKFLSLPPYSPETLFCEPEQPLCNSRAGCYPALSQMNQFQGHLMVNRPVEMQEQRFYCLGPGFGFLKSCFACKKMLSCDKDIYMYRDLCAFCSTDCRDRQIMLDEQAERTEMGSTTVDSSNSMQCYADVKHMNVRVAA |
| ***Musa acuminata*** |
| >MA01G14700  MLRKRTRSVSNKQGLMSDTASLLSPTAASFFTSPRPFSGFSQKGTADPEAGASPTSILETRPFSAIRNPSFFDGNPKRAILSSSVTPTIPNVSRPLPQENGDPRAIGLGLLDVLNNEDSVKSTSKPEKRMVVFGSQLKIQIPPPPTAPTQNSKLALYSPQKSPLQTGYVGSDMLNSSPRVFTGCLPQSEMELSEDYTRVILHGPNPRTTHIYDNCIIESCGNGWNEKGASHDQPSYTADGFLSFCYGCKKKIGPGDDIYMYRGEKAFCSHECRDQEMLSNEGKEDH*  >MA02G04630  MADSGDQKKHYVLKLALSVGFSDSSSSAADAAHDRHSNSRPAAAARSPRSFAAGDGAVGLAIVAAMSAASEEPAAGLARSDPVPIGAVVTKPGLRTAAPKEGEVGEDMELSESYTCVISHLGGNRVKKRVYFGDDGLLFEPPPPPPPGLADPPFVVAEFLRCCFLCKKKLDGMDVYMYRGEKAFCSEECRCQQMLHDELGEQFASGAPKNYECSSSPYASSLVISAGVAAA*  >MA02G09020  MSDPASLLSSNAASLFTSPRLLVGFSPKSTADPEAGACHTSILETKPFSAIRNPFFFCRNSIMAIFSATVSPISANECRQLPRENGEPRAIGLGLLEVLAAGDSVKKTSKTEKRMVVFGSQLKIQIPPLPTDPPQFSSTPTTDSGDSLQSPIEFGIKTRNSLLALYSPARRSSAETGRMGSDTLNSSPRVSTGCLPQSEMELSEDYTCVILHGPNRRTTHIYDNCIIESCCNGFATSANEIRSSNDQSGYASDDFLSFCCGCKKKIGPGEDVYMYRKRAFCSHECHYQEMFDEGKEKC*  >MA02G10570  MHHVLSYQPHRINSERADQASFSNGLVAWIPRPVAVWKPPVPLSLSLHKTTHPINVVPSTTSLYTNLLLLPSETDPLSSLPICHYILAEFELMGSSSFSSSSSSSDGSLSTVEIQAGFSGCSSYVPSPPPSCYGSAMLGSLSSLRPRSLFFDGLDDEEDDEPRHFLDSCSLCRKPLARNRDIFMYRGDMPFCSEDCRKEQIEIDEAKEKSWKLSIKTSSSRKEQQRKIAAAGGAKSEKIHVRAGTVVAG*  >MA03G26140  MLRKRSRAVGSKQGIMSDIPSLPSPVAASLFTSPRLYSPKNIADPEAAVSPTSILETKPFSVIRNPFFSNGNKHLPQEDVESGATGLGLLDALTNDDSFSSTSRPEKRMSSSISPMRSSGDSPPRFFTGRLPPPEMELSEDYTRVILHGPNPRTTHIFDNCIIESCTNGFATPMSERRSSSDRPGYAVDDFLSFCYGCKKKFGSGEDIYMYRGDKAFCSHECRHHEMLLDEGKDKC*  >MA04G01850  MLLGKRQRPPMRRTTSMTEFAADVEVMQQPFDQEKVSNALHHQRDQSRERQTASDWLETKNLGNPAAPSPRGGRCPRNSAELVVVDTAPFLRACGLCKRRLGPGQDTFMYRGDAFCSFECRQQHITQDELQEKCSLTSMKDTSTATTGSEPAGDSEMVAAA*  >MA04G21870  MWRSKSISKLIIYYDQHVVAPEDFQLSGKWRSKTWTWLCLLGNGDRTWFGLKRFQCQTQVFTSAEISMYQPNIFLWKRSRAVGAKQGLMSSFHGGSSCGKPAASSLLPSARFSEAAMSPTSTPETRNLSSIGNHIPPFDAITTSSASVSDTKQHPWRNRGSRPTGLGIIDALSDEQFDTKPQRSMVLLGTKLKIQIPSLCPTSNSPVGSPIEFGIKNRDSQLAHLSPAQSSPGSEMPASSPRVFAGSNFSMSEMELSEDYTCVIFHGPNPRTTHIFDSCAVEQCGDGFTRSTKSRSFAADSRGYPLNESLSFCCACKKNVEQGKETFMYKGEKAFCSSECCIKGMLDEYGIETSAQRSHHSRCDPCF*  >MA06G02040  MLLRKRQRPPMRRTTSMTEFAAEVEGPVTADQVGAENQSRRPHILQKQRNQRRAAEAALWRSGGAGGRRRNSGNFAVVETASFLMACGLCKRRLGPGRDTFMYRGDIAFCSLECRQQHMNQDEPKEKCSLTSLKDTPPATNGSESSDGGETVAAA*  >MA07G05820  MPSPRFFSVHEARRHHFLGSCYLCKKPIAENKDVFMYRGDTPFCSKECRWEQMDMDEALENESKKQSFAMKGKPSLTIPSNCDKKSFKADAAAVVAG*  >MA07G18140  MLLEKRPRPPMRRTTSSTEFASSVLFDVEAPQPFDQVAIGVRHLESRWGADWRTTWYTGSMLSPRGGVHRRSSGDFAAPFLRACGLCNRGLGPGRDAYMYRGDIAFCSLECRQQQMNLDEQREKCSLTSTKEMPSAASGSKPSDGGGTVAAA*  >MA08G15350  MESSSFSCSSSSSASSSSASDLEAGRAAPYNSAGAAAGSRSPKARFFCDGLDHEEPHHFLDSCFLCGKPLAGNRDIFMYRGDMPFCSEECRQEQIEMDESKEQNRKVPPKASSSSKDSSKGGTATGPSKSHKVHVRAGTAVVAG*  >MA08G19100  MAEGTGEGVAAAILLGKRTRQPMRRTTSMTEFSLEDVAAAAEGREGIRRHHKCQHRERRRGPEDEGPPRIVQRWSFGDLGTMETTTKFLMACGFCNRRLGPGFDTFIYRGEIAFCSLECRQNKINQDEIRDKHLRSKKTNAASTAVSETSGDGQRAVAA*  >MA09G13270  MVLRKRPRPPMRRTTSMAEFAPNVVLPNDKASQPSSDHRKASDARYQWVLDASPCGGRSRRNSGDFAVVETAPFLRACGLCKRRLGPGRDTFMYRGDIAFCSHECRQQQMKQDERKEKCSLTSIKDAATATNGSEQAGKGETVAAA*  >MA09G20030  MLFEPPPPPELAEPPFVVAEFLRCCFLCKKKLDGMDVYMYRGEKAFCSEECRYQQMLLDELGEKFSTGALKNCECSSVPFRVTTGVAAA*  >MA09G26330  MLRNRSRAVGGKQGLMSDSQPFPSPTGNSLSKPTASSLFPSPRLFRSFSSKGFADSEVAMSPTSILETRHLSSFGNVLYSDKQPKKPPCEVVSASTESKHHSRNYGRPEPIGLGLVDALNDDKAKESSKAERRMVVSGSQLRIQIPSINSNSNSLGRSLGHHHEVSVSSPRVFTGSISASEMELSEDYTCVISHGPNPKTTHIFDNCIVESCGEEFMPARKDNSFVPSQGYPADDFITFCYACKKILGHGKDIFMYRGEKAFCSNECRYHEMLIYEELDNHSTDSSLKSSPSFSSSHSCTMQETHLLLAHCSQAITFRLPNRCSLTTC*  >MA10G24980  MLLGKRPRPPMRRTTSSTEFDAGPRHGATEVAQRTAGTGADWRAASYVGSMLSPRGGVHRRNSGDFEAAARTAPFLRACGLCNRRLGPGRDAYMYRGEVAFCSLECRQQQMNLDEQREKCSPTSMKDTPSPTDDTEQSGNGGTATAA*  >MA11G23780  MEPFSILYSSSSDLEAGWGPPYAHPTPPSPNGSAGGVARPRTPTYRFFCVDVDGPPHHFLDSCFLCRKPLAGNRDIFMYRGDTPFCSEECRLVQIEMDEGTEQSRKHSLKASSSTDSNKAGAATSPSKSHEAHARTGTVVAAG*  >MA00G03980  MLRAMNKIHKDHGTEQLVSDSPSDSCFSSKGLMQTARNTSLFGVPGVFVGFSTKGFSNSDAVRSPTSPLDCKAFSGLGNSFLGSPRSASLDGKPRCWDCNRVGLSLVDALKDEAEPCGKFLGLSESRTSAFAPATRIDISCHKSHLAGLRDDSSCAAPKSLPKDYAISSHTLDSNSSMVDSFRLVERSASFDKFSHETIGPSYGVITSLSASEIEQSEDYTCIISHGPNPKTTHIFGDCILESHSFPSLVFENKCRTDDCRSAWLLKPSEDSPPCSTDDFLRSCLSCKKKLEEGKDIYMYKGEKAFCSSDCRDREILIEEEMEKLTIDSSGTPGSSFHEDMFADGKIMTA*  >MA00G04940  MTRPQRSRGRSLEITMDSSRSSSFSSPSHGSLNDAGPEAGNSSAALGRLYYDAFDDEPRHFLDCCSLCRKPLASNRNIFMYRGDTPFCSEECRQEQIEIDEAGENSRKLSVKVSRSRKERRKSGESQKIHVWADTAVAR  >MA02G02250  MAGLSVLLEAQKNSPTHSHLLISQTSLGKNTSSSSSSSSSSFSTSSFLEHCFLCRRRLQEGSDIFMYRGDRAFCSEECRCRHMFVDEESRRAEHCSSAAPASAAGKGRATPGGFAY*  >MA03G04480  MLLGKRQRPPMRRTTSMTEFAADVVLTDPQRAADLVLSPRTGGHRKNSADLAVVETAPFLRACGLCKRRLGPGRDTFMYRGDIAFCSLECRQQHINLEERKEKCSLTSIKDAVPAANGSENAGNGETVAAA  >MA03G12880  MRASVRVPAFPESSSLWLNLFSILSMFRNKNGIGIRNRGKPLIILTQRACVCRRERIDRERVASRRSRSPRPVLLDSFLRLHRISLSLRRALFASLLTTTSDETKALQNTKLHIEVSLILLLLLLLLLLLLSLSLSLSPVPGMSSPELFSVVVDEEAPHHHFHDACFLCKKPIAGNEDIFMYRGGTPFCSRECRREQMDMDEALEKDRVASYTKCSSLTWLLHTEETHEGKS  >MA03G24740  MAGLSVLLEQRAFPKYTRIVSKTSVLKNSSPFSAAPTSSSTQGSTFLEYCYLCRRKLQEGQDIYMYRGDRAFCSEECRCRQIFMDEESGKRDHCSLAAAAAADGKYRAGRRRPRAAAGERALAGGFAY*  >MA04G20460  MPSPRFFFVDEGRRQHFLGACFLCKKPIAENEDVFMYRGDTPFCSTECRWEQMDMDEALQNEARTQSPPPVKRKPSLIVRSECDENQFVSDATAVVAG*  >MA04G23270  MLRKRSRATDGKQVLMPDAKPRPSSLFPLCLSYHGSSDSEAAMSPTSILETKNLSSTGNHFFSDRQPNKPPFDAVTTTTTTTTATSSAIGLGIIDALNGEKSVAISPKAQSRMLLLGSQLKIQIPSLCSGSMSPVGSPIEFGLKNRDSQLALLSPAPASSPRVFTGSIPMSEMELSEDYTCVISHGPNPRTTHIFDNCVVERCGDGSSAADRGGYLLDDFLSYCHACKKNLGQGKDTFMYRGEKAFCSSECRNKEMLNDGGMEKCSEESSLTIDGSAQTNVGFGTTIFWLRERERDDKCVTAICCHARFWYQSFSWLREPHVKVICQLLSSCYFTLRRPGGFMQPLLPHIFRSSYSLTYSFCFLCGSSRASRVLSC  >MA05G12950  MGGPLSPPGPRRLFSYGFDEDEVDGECEPRHFLDSCSLCRKPLSRNRDIFMYRGDTPFCSEECRQERIEIDEAKEKRWNLSAKASGARRQVAG  >MA05G23130  MLSRNKSDSHQGEDGGETRVSVVGGRCVRHHPSEGLRILIHHKEQATNVVIKAKMMMMMTRKLVSQACHLPKGLPEFDFLKSCFLCRRELSPCKDVYMYRGDQGFCSKECRSRQILLDERREGERTTGERSKISHRRRAITYGLVGQIILI  >MA05G25010  MFMGKQPGTPMRRAPSSTEFSPSVLFDVGGPRPSDHNAIVHYLQPREAAQVESIPSPRSGVHSGRSGDGTAPFLRACGLCNHRLGPGRDIYMYRGEIAFCSLECRQLQMNLDEEKEKCALSDLSKE  >MA05G27750  MAGLSVLLETQTNFPKQAQILSKTSFLKNASLTSSSSAVAASTHESTFLEQCYLCRIKLLLGEDIYMYRGDRAFCSEECRRRQIVMDEEGGMRDCCSLAAAAPAESRRERAAVRGRALAGSFAY  >MA06G21050  MAGLSVLLEAQNNLTKYTHIISKTSLMKNASLPSTCTNSSSFSASHFLERCYLCRRRLQQGNDIYMYRGDRAFCSVECRNRQIFMDEENGRRDNCSLDAELQAERGRPRVVARKGRATAGGFAY  >MA06G11950  MVHQPRPTVPKLPDPIRLCDRMWLIGGAAERNPQSPRGWRNRDPDGVGLGIAVALEKAGGDSPRWLRVTAPIAIRSPRASGPQVAGPGCSGELHRKRPASCRLPPPAAEHFGVADFLSSCFLCRKNLHGKDIFMYREKAFCSSECRYRQITSDEYQEKRGAGAPKPSEEIATSPYSGDQFFFTCIVVS  >MA06G22670  MFLRSKIQDIILRERSKAVSRKKELMPDTTSSPPPNPAFTSPNLLVEGCSVRKSSMYSEDAIGPKSMLATIPFFATGNRSSSDSRDCKTKPLSVETAAATTGTESRHHPWHNRSSRAIGLGLVDALSNEETGDVGCTMVVFGCQLKIQTLPGLSNSMSPVSIESPRSIMEFGSKTKTSQMDSYLPGRTVSGSVFSPQLFMGHMSPTEIELSEDYTCVISHGPIPKKTHIFHDCIIESCDREFLTLKKSTCMDDPSGYAADTSLSCCVSCKEDIGKGKGISLYRGEKGLCSHECGYQEVVSDE  >MA06G37000  MLSGDDSSFHLSNRGLRYMKQQPSEGIEGLRILIHHKEQGANVVVKAKMMMMMMPTTTTCNLNSQAYHLPKGLPWLALLKKCCLCRRELSPANDVYMYRGDKGFCSEECRSRQILLDERREFEVATRERSKLPRAGNEIGESDRSRRIPTLV  >MA07G10050  MLRKRSRPIQKDQSKGFLMSHPASGSCFPSDGVAKRTSCASFLNVHGHLVGFSNKKGLSESATVWGHTSPLDKVLSSITRSTTGIIDGRLRCWDSSRVGLGLVDTLNDEAKPCVKVLGSSESRNIVFGSQMRLNFSTPKSHQVGPRDDPLGAAPQIGSSKPRSVCSEMAIELKGSKLVHEEYGLLRSCSVDTGGFSLLLTKSIGNNCKSNSEVLRSESMDILDSPPLAKEDTNFDKISVSLPISFGSSHRFIGSLSASEIEQSEDYTCIISHGPNPRMTHIFGDCILESHSIESPKIKNKHRKEDEGSTWLSEPSEEMLSCFLNDSLSFCFSCRTKVDDDGKDICMHRGKKALCGCECCRNGTLLEEKEKTRIISSGSPGSSFHEETFLDRKHCLLMDVIDLSFYLAVNKTLLAI  >MA07G13620  MNKVQKDHCKGQLLSDSTSDSCFSSNGLIQKARNATLFSVPGLFVGFSAKGLSESDAVRSPTSPLDYKGCWDRTRVGLRLVDTLNDETKPCGKLLGLSESSNILFAPRMRINISSPKPHLVGPGDDCTGAAPKSLPKDYRTSPQTGRSKGAESECGEVGLLRSCSVVVSTSSSTPIDSKSESEVFASDSKSSMAEGSANFDKLSGSLPVSIGGSPCGVIASLSASEIEQSEDYTCIISHGPNPKTTHIFGDCILESHSFPSRDLRKKHRKDEERSAWMLKPSEEDAPPCPSGDFLRCCFSCKKKLEEGKDIYMYRGEKAFCSCDCRDQEILMEEEMEEKPTFGSSGSSTSSFHEEIFLEDMTMAE  >MA08G06780  MAGLSVLLETQETFPKDTHIISKISLKNYTSSLTSSCSTRRSTFLEDCYLCRRRLQHGKDIYMYRGDRAFCSEECRRRQIFMDEESGKRDNCSLATAASGKHPVGRRGGRATARRRALAGGFAL  >MA09G18520  MLPRTESIFHLGEEEGGEAPESAAAITELDCSKYEGLRILIHRSGRPSNYVVIKSLLKPFTFTPMASPPRFGFLRACCFCKRELSLHKDVYMYRAGESRGSAARNAVDAKSCKMRGVNSERRRGRGRRLLFLIMLPPELMILADAGGSQQ  >MA10G17880  MVHQPRPIIGKLPDAFGLGDRMWLTGAIPSPTTPSERNSLYPRRWKNSDSGGVGLGIAVALEKAGGESPRKLHATTLPIAIGSPRTSRPQVCPGGRHWERAQGGCCASPPPVATEESQVADFLSNCFLCGKRLHGMDIFMYREKAFCSMECRYQQMVSDEYQEKRGVEAPNPSGSPYSGGQLFFTAIVVS  >MA10G31830  MLSRNKGGIFHLGEDETTKTIVSIREPKHMRPASEGIEGLRILIQHKEQGSNVVTKSTCSLSKGLGFLKSCFLCKRELSLSKDVYMYRGDLGFCSKECRSRQILQEERREFEMAARTRLREPQHRLRASARIRDQDRNSRIPGVA  >MA11G18630  MAGLSVLLETHNSLPKYTHIISKTSLVKNSSLSSPPSSFATSPFLERCYLCRKKLQQGNDIYMYRGDRAFCSVECRCRQIFMDEESGRRDQCSLAAAAASAADAGAQSDRGRPGRATRKGRAVAGGFA  >MA11G26210  MAGLSVLLETQKTFPKHTRVISKTSFLKNSTFSFSSTSVSARRSTFLEHCCLCRGKLQQGKDIYMYRGDRAFCSEECRCRQIFMDEESGTRDRCSLAAAAAAAAVAEGMYPAERRRGRAAACRWFC  >MA06G04180  MLLGKRPRPQVRRTPSSAEFSSAVVFDVEAAQTSNQDAIACHFNPRETRHVGSTLFLHSGVHCGNSGDGTAPFLRACGLCNRRLGPGRDTYMYRGDIAFCSLECRQQQMNSDEQKEKCYLTSMNDNPLETSCSNQSDSKNQVLTRYKLHVFQSVS  >MA08G31290  MDFYCESLSPPTPGEFGGFQVADFLSHCHLCRKRLHGKDIYMYRGEKAFCSMECRYQQIVSDEYQENCGSEATRPTEISSSPYSGDRIFSPGIVVS |
| ***Ricinus communis*** |
| >RC29794G01750 MYLSKSVAFVTGKRGCGVPNRRFLGVMLRKRTRSLQKDQQMGPLTMSDSGSQFNSQSDCLGYNHKRTSFFNVPGLFVGLSPKGMSDCDSVRSPTSPLDLRLFSNLGNSSYRSPRSSQNGHQKSWDCSKVGLSIVNSLDDEDDDTKVSGKVLRSSESKNILFGQKVRIKTPTFQVNANSFEAPKSLPRNFAILPHSYTKSSLQKGCSKVIFEIGEAPTEPEHFGKIRSCSLDSCKSFSTLSRLANRNSNVICGNFPLNNVATGTSSPLQFSGGSPPQSNNSLHMDLNLPPAGSTSGFVGSLSASEIELSEDYTCVISHGPNAKKTHIYGDCVLECYSNEGKEIRMPQAITSSIIPSPFPSNDFLNFCYYCNRRLDGGKDIYIYRGEKAFCSLSCRSEEIMIDEEMEKTTNKTCDEPEPPKCDNGEELYENGIFDAP  >RC29822G01940  MESFSGTRRVPYFIEEDDGLVSLADMEAGFSGNQQQQQQKHQHPFFSRSLCYGKEGSMRTISSSISSPRSARFYDARFEDHQPHFLEACFLCKKPLGDNRDIFMYRGDTPFCSEECRQEQIEIDEAKDKNWNLSSMKKLRKKDKKRSSISPTKAQDYPSRTGAVAAA  >RC29703G00380  MLRNRSRAVTSKQALMTDHSSHSPSTQNHTKPIPSFFGSPRFKGFTFKRSPEAEPVISPTSILEPFSSFKNPFCHDTNQPKSPRVSSENKYSWDKLDSKGIAVALIDEEKPNEQNNSKKISKPSNKMVLYGTKLRVQIPPPANFMFSAADSPISPGDFGIKTRNAQLSGSGSGIQTKESPGVFTGCVPMSELELSEDYTCVISYGPNPKTTHKFGNCVLENYCSLSDKSNSAPNNFLSFCHKCKKNLEQKIDIFIYRGEKAFCSQECRYQEMMLDGIES  >RC29844G01130  MIRKRSRVSSSKQVLMADYSSILSPTEKYRKPTSFPRLFTGFSFKNFSETTESVMSPTSILDSKPFSGFRNPFLPDQNLTPKTQESDTKRTWDKLDSKGIGLAIVDALNYDDKTDSNLSKPESRMVLFGSQLKIQVPPLPVSPTDQSPKSPADFGIKTRHSQLGSSSSGLSHSPVKKSVCGSANSSIDTSSSPGVFNGSLSAIEMEQSEDYTCVISYGPNPKTTHIFDDYIVESCCDVVEFSTSRTQTNGFLGDGSSYPSDNFLSFCYACKKNLGQGKDIYMYRGEKAFCSSECRYQEMLSEEGIDKLDHEDGYGTCSQ  >RC27810G00300  MMIKLIRIYQNWKAEWFYPGRTKDPSCLILQLRNQYVVQPIRLSDNFLSFCYACKKNLGQGKDIYMYSGSNHKKCSFYIGEKSFCSSESRYEEKLLEEGIDKFDDEDGYGTCS  >RC29739G01590  MLGKRTNPMIGRLSELLVSGNRAAGFLDVATAPSPRSPLDYRIQSPRGLKNYDLGGVGLGIVAALEKSTYSSSDGTSSGHEILAKYAILCSSNNTTRSDPIPVKIGLSLHKEMLEIDSLDDYTYVTTHGPDNKSMTKVYYDHGQKGHRRIGFDSTTDNSFGVVSVIKETPAPARFVDEVAYPTSDFLSSCHLCKKKLHGKDIYMYRGEKAFCSAECRSRQIMIDERKEQCRSEVQRSADVSSSPYTTSPIFSAGILAI  >RC29709G00300  MSYYGQQPHFLEACFLCRKPLGYNSDIFMYRGNTPFCSKECRQEQIEMDESSSRKKKSWKISSSSTARSIRNSESEDNSPNTPSVRTGTVAVA  >RC29872G00230  MADSALESHCQSDALGLKHISSSFFNFPGFFVGFGSRGSSESDSVRSPTSPLDFSFLSSLSNPFSLKSPRSPSQNDHQKNWNSSKVGLGIINLLADETKPPGVVLNSPKRKNIIFGSQVKTGYSVRSNSLPRDYMLLLLPKTKTLNRQLGKSNSEAVFGVEAVQLECKPFENSSPITLSPKSPLISKKFCSENRTTTITSLSFFDDGGTPTDDSLGTKSSSLPVPIGSSKGYVGSLSARDIELSEDYTCIISYGPNPKTTHIFGDCILECHTNELSNFDMGSELPQETNSPLPSDEFLSFCYTCKKKLETRDDIYMYRGEKAFCSFNCHSEEIFGEDETEKTYDNSPKSSSMSSYHEDLFLTIDATDESWAAIA  >RC29908G01430  MLLGKRPRPPMKRTTSLSEITFDLDTNGSCESAQQAAGFGGDGTGTGGGQQQLDQRFLAAATISPRNHRRASADFLETAHFLRSCSLCHRRLVTGRDIYMYRGDSAFCSLECRQQQMNQDERKEKCSLASKKEVTSSTVAGADVSAKGETAAALWGHVPFKHHPANSPLNYI  >RC28348G00010  MLRGYSSRESSEKGLMVLVGLQTLVHVSEGKSKSNVVTKSAMRKAPHHRHRRPHHQSTESCYLKTCHLCNKNLSLDKDIYMYRGDQGFCSIECRNRQIVLDEMRELEASTVQMRKSYNRHCSSAGRHETRLILEEIRRRHKPVIDDHQKKHWAIV  >RC29726G01810  MVGLSILLEAQQKQQVGFANNNKKKTPQVINKATLNMMMINKLPSPLPSSTSLSSLSSFKPPTFLEHCFLCGQKLLPGKDIYMYKGDRAFCSVECRCRQIFTDEEETLRKENCSFAAMKPTSTSAATAPPSSSTSASRHSKSTRNRAGGFAY  >RC27905G00010  MSGINVDLPNIDALEASDYQNHHPNNNNNHSNSSNNHNSQQYGSYEYDQLFLAATMLSPRNVPTRRNSADYLETANFLRTCGLCQRRLAPGKDIYMYRGDTAFCSLECREKQMKHDERKEKCINAIMGSKKEDRHASPSTTNSASKSSSSSSRKTETVAAA |
| ***Carica papaya*** |
| >CP00557G00010  MLLGKRPRPPMRRTTSMTGITVDVSGVEASEPFDEQNVHHVAAATGGAEESSAVAISTALRDQNMLMNSAAYDRRFLAMVSPRNQRRTNSRDFMETADFLRTCGLCKRRLAAGRDIYMYRGDTAFCSLECREEQMKQDERKEKYGVVSSTRSENHEYHRHHGASSPT*  >CP00016G02030  MVGLSVVLETQKIGGVNGGSNRGGVEKKSLQVINKTMLITSNNNSSNKSFSSFSSPRNLSPSFFSSPPPFLDQCFLCKQKLLPGKDIYMYKGDKGFCSVECRCRQIFMDEEESLKKENCSLAAMKPRQTSSSSSSSSSSSSSSGNSHHHRKGARNRMGGFAY  >CP02061G00010  MATKESIGEGKLGSFLEHCNYCKVKLGPHSVVYMYGNFGAYCSLRCQTKQMKLDQQIEEESANKSAKRNMNDAKTHIQIEEESANKNPKRDMDDAKTQDVKDLDDSHIG*  >CP00066G00790  MLLGKRPRAPIMKRTTSMTEITLDLNTSTTDSRPSDPHNLFDARQKPAVTGALSGQHSHGAGHNWLDQRFSPRTNRRHSADFLETPNFLRACSLCKRRLVPGRDIYMYRGDSAFCSLECRQQQMNQDERKEKCGLASKKEGGGSSGTGAAANGETVAAV*  >CP00025G01840  MLPKFKSSSVNPSSDVGLLILIQTSHGKSNLVIQSALKSVQPTSQSTEFCYLKSCHLCNKKLSLDKEVYMYRGDRGFCSTECRNRQIVVDEVRELEISTKQMIASYRNCSGGGGGGGRETGSLLEDLHRQNKPPTHRNHWAIVS*  >CP00052G00690  MVTEVPDYFRFTAFERLRQISFPARSFSSNFFFFFFFFLFYKQRSPMAFYYAGGVDQREPQPHFLDACSLCSKPLGYNSDIFMYRGNTPFCSKECRQEQIELDEANEKKWRMASRSVRKSSDSNNSSPSKAVRTGTLVVA*  >CP00487G00060  MLRNRSRAVTSKQALMADHHSPTPSPTKNYTTPITSFFNSPRFKAFTAKTLFETESLKSPTSILDKQTLFPLSNNPFLQDSQPKSPKSVPDKFEKLDPKGICLALIDDKPIEEDNDSTRPTSKMVLFGAKLRVHIPPLPPTSTISPSESPRSPADFGIKTRNSGFGSGNTGVQTVKDFSPRPLSVSEMELSEDYTCVISHGPNPKTTHIFDDCVVEETYRCLSEKSISPASDNNFLSFCYTCKKNLDQKDDIYIYRGEQAFCSRECRYQEMLLDGMEN*  >CP00028G00090  MIGKISELLVSGNRTAVSDGVTSPRSPLDLKIQSPRGLKVYDVGGVGLGIVAALEKKCNDVGRHEILVKYAVGGSNRSDPIPCGKNFDKFKPGFEDLEMESLENYTYVTSHGPNRSTTKVYYDGVRSDERIVCNNYSAVKESPARFLDDLSGYPTLDFLSSCHLCRKKLHGEDIYMYRGEKAFCSTECRSRQIMMDERKEQCRSEASRSAETSTSPYSRGQIFSTGILAI*  >CP00205G00320  MLRKRTRSPQKDQAMSHSSPESCLQSEVCWQNNPKNNSFFSIHGLFVGLNSKGLSESDSVKSPTSPLDFRVFSNIGNPFVRSPRSPQHGHPKSWDSSKVGLSIVDSLDHDTKIPGKPLQSSESKNILFGPAMRIKIPKTHFHSFQAPKSLPPNYVTQIKSPQGKGNSDVVFEIGENLLELEPFGKTRSCSLDSCRSFSALSCVADRKTLTTASGLSSPPKLIGGSLNSNNFLRRKVNPISVSLGSANGFTRSLPTTDFELSEDYTCVISHGPNPKTTHIYGDYVLKCHNNELTNCSRNEGNEIGLTPAVTGSTSVWNSDNFLSFCYSCNKKLEGKDIYIYRGEKAFCSLDCRLPEILIDEEMEEEDMNKSPENSPNKDNSQELFETGIPVVT*  >CP43574G00010  MATKESSGEGKLGSFLEHCNFCKVKLGLHSVVYMYGNFGAYCSHRCQTKQMKLDQQMEEESANKSLKHDMNDAKTQVQIEEESANKNPKCDMDDAKTQVQIEEESANKSPKRDMDDAKTQDAKGQDDSHVG* |
| ***Medicago truncatula*** |
| >MT8G008840  MAANYSFSSSSPSSSIPSPKSSMFYYGGSEDFYDEPHFLQACYLCRKPLGQNKDIFMYRGNTPFCSNECRQEQIEIDESKEKSWKISTKRGVRNSETNQNSSNNKAVRSESVAVA  >MT7G095170  MIFLFGRVLMLLRNRSRAVTKPNLMADHSSQQSSNNQNCAKKTIPSLFGSPKFIRDFTTNNNNSILSEALKSPTSILDTRMLSPYGFGYPLFYDNKIQTVPNKTFSSKGIGLALIGNLKDDESIDENNSVEQNKGNVLFGTQLRVKVPTLPSPIYSPFESQTKTKDTKNSKLLSLSEMELCEEYTCVISHGPNPRTTHIFDNCVVEESYCSLPQNNSNSSYGNFLRVCYTCKKHLEQTKDIFIYRGDKAFCSRECRHREMVLDGVESLEEF  >MT4G120160  MVGLGVVLEEQQPHKKKCNININNNNNTYQVINKTTMMLSSTINNNASYPLSYHSPFKVSTFLDQCFLCSKKLLPGKDIYMYKGDRAFCSVDCRCKHILADEEEATKKQNIFEE  >MT1G103500  MEDSSSKKITSSFFNSPKLFTSKGFHETETMMSPTSILDTKPFSCFKNPFWSETNSPITQHVCGEHKRCWDKLESKVVGLGLVDVLVDEKNSEVNSKPENKTVLFGSQLKIQIPPFLPTISTFSSSSESSPLSRGDFSINTRNSHLGSSSGSFSLSPVGKSSFGCANEIETSNSTHGVFKGCLSASEMELSEDYTRVISHGPNPRTTHIFDNCIIESSCFDIGCSAVKENGYCFPHPTSYPSESFLSVCFHCKKNLEQGKDIYMYRGEMAFCSHECRDQRMLLEDVMPI  >MT1G103510  MESSKSYTRCFDVGCSSSVKENELRFPHPIRYPSEIFLSICFQCKKSLQGKDIYMYRSMPFCNNECRNQRIRLDEEEIPKLKTKT  >MT8G022950  MEVGLSLLLQIISSKSNSNILVKSAVKKSNQTIPMDFCFLKTCNLCNKQLSQDKDIYMYRGDQGFCSIECRNRQIVLDEMKELEISTKKMVQCYRQCSNEARRETRLILEDIRMQRLKNKV  >MT1G010140  MALGSNEKRKSIFKAPLLYVGLGMDSDSLRSPTSPLDDVTLLSNRGNTLRTSPPNQGQKRGWNCTKVGLSIIDSLEDCSKFSRNVLLSSEFNKGSFSPNPSPQMITKVTNCNHCLDSVMASKSLPKDFFKLPYTKNNSVFHKGESNVVFEIGEPLLEHELPFGKSMSCSLDLYSPIKNSKFDSKCENFCLKPVSFPNTLLPVSLSASEIENSEDYTCVISHGPNPKKTHIFCDCILEVHADDDVKKHQNKNEEEGSSSPVVGRLETPNQFPSAEFLTFCNHCNKKLDEGKDIYIYRGEKSFCSLACRAFEIMIDEELEKSNEPPENSIELESGEEHFGSGIFTAV  >MT1G071780  MLLRNRSRAVTKQTLMGEQSPNQNFTTLPSLFGSPNYRDFHFTSKKSLTGTEALISPTSTLDTKALTSFENPFSITNSNKVCSTRDKVASKTIGLGLIGVLKDEPSHQNSDHKLSNRKVLFGTELRVKIPPLPPSMFESQKSVLSLSEMELSEEYTCVISHGPNPKTTHIFDNCVVEESYCSVTNSPHSFSMNFLSFCYTCKNHLEHTKDIFIYRGEKAFCSQECRHKEMVLDEAEN  >MT3G073960  MMLGKRPRPPMKRTTSMSEITFDLNTVTTEEDPNNNNNLFNRHGPGVGPYGPYPPTSPGINGSDQSRVMSMVSPRNLRRNSADMTHNPDFLRSCFLCKRRLVPGRDIYMYKGDSAFCSLECRQQQMNQDEKKDKCSVVSKKQVVVAAGSKVVATNL  >MT2G102237  MADSSSNLSLPPDTVSARQIRSSLFHTSGSRVGAGVKNLPDSESAWSPTSPLDYRLFSNLSNVFSAKSSRPSFQTENKKPLDGSKVGLGIITSLVNETKPNNEILGKFPRKNIIFGSQVKNHILQFSKNNHESLAPFLKTNSLPKNYVISLPSETKSPTLPSKTKSPKSEVESFDDDVNRESKGLRSSVVSSPDSSRPSSLINSNQSSNLGTNDLFVDVTSTPLSLLPVTNTSSQVDDSLKIISSSLPVSIDFSNGYVGSLSAKEIELSEDYTCIISHGPNPKRTHIFGDCILECHNNDFTEFSKKEESAPHRFDSVMSFCYTCDKKFDEEGEDVHAYSDEKAFCSFKCRSEEILAEEEMEKTCTNTAKSSPNSSYHDDIFLMGLPVSK  >MT5G084640  METGPKRRRFYKSQSTICLDDLSSDANVAHVNTNDQRIIEKPTSNFVKAPLGQRLFSLGFPALDAPTKRTSPALPEGFLHACRWCRKKIEKDRYMYGVFSAFCSLKCRENQMIADGYMVEICSTSGSIAEETGGIKNK  >MT5G006830  MENGPKRRRFYKSQSTICLDDLSSDANAAHVNNDQRIIAKPTSNFVNTPLGQRLFSLGFPAFDAPTKRTSPTLPEGFLHACRWCRKKIDKDMYMYGDFSAFCSLKCRENQMIADNYIVEICSTSGSTAEETGGIKNK  >MT2G436020  MVGLSVVLESQKGGGISNSKKKETPKVINKTMMLSSINNKQSSIVSFSSYFHHESHFQEQQTTFLELCFLCRKKLLPGKDIYMYKGDRAFCSVECRCKHIVMDEEESNNIQNENCYYAAISSSSSEASYHHEKGRRNQNGGFAY  >MT3G108290  MLRKRSRSIQKDQHQMGHLTNSDTNSDHYAQSHALGRNIKGNPIFNVPCLFVGLGPKGLLDSDSVRSPTSPLDTRVLSNSGNPVRNLRSSLLEGNQRSWDSCKVGLSIVESLEDCNCSRFCGKILQSLDSKGISLSPQSMIKTPICETCMDSFESSSKSLPKDFGKVVPCVEDGSVIQKGECESNVLFEIGETSLEHDEPFGRTRSCSLDSCKSMKADFGLATSKTDSDIDDFAMKDVTVQVSSSPHFIGGSQNSNAFIPAESKSNTLSICSSSEILKSLSASEIELSEDYTCVISHGPNPKTTHIFGDYILETHPDLSIKNHFKNEENEKEKGVTLMGNKLSQTPNQYPSSAFLSFCHHCDKKLDEGKDIYIYRGEKAFCSLTCRAIEIMIDEELEKSNSPCENSAKPKLGEQIFEAGIPTTT  >MT2G436250  MVGLSVVLESQKGGGISNSKKKETPKVINKTMMLSSINNKQSSIVSFSSYFHHESHFQEQQTTFLELCFLCRKKLLPGKDIYMYKGDRAFCSVECRCKHIVMDEEESNNIQNENCYYAAISSSSSEASYHHEKGRRNQNGGFAY  >MT3G087700  MLLGKRPRPPIMRRTRSMSGGLSVDMQAHDQTNNLESHHEKESVMSHHNPLQPHPHHDDHQEHGINIKNPHTVVMGTETHIQSKLTVSDERLVGSAVMFPSHTNNIINPLSASAHDVIHSTPHFLRTCGLCNCRLAPGRDIYMYRGDTAFCSLECREQQIKQDKRKEKWKIAFTNKEDHRVSPPCTATAKASTAACT  >MT5G013770  MATSSKRHCFLNDEEDDGLASITTTDIEPGFSGHNHFSHQHGFVSRTLGYGYATVYNRGLRNNANSPRSGGRFYDARFEDHQPHFLQSCFLCKKALGNKDIFMYRGDTPFCSEECRQEQIEIDEAKEKNMNIASSMALRKKEQRKSSSPNKAQDYSFRTGTVAAA  >MT2G042970  MLGKKSHHLIRKFSELLVFGESTTGLFDTIGSPRSPFELSSKIQSPKGLKSYEFGGVGLGIVVALDKSDNDFVGYEVVPKHVCTPKTNQSGEPITIPIQNHHQQNGNFANEILVGSPEDYTYVTYHDESNKPITKVFYDGGEESGILKHDCYTSKNINNNNVGVFKRSPPTQTLAQAEPSYPTLDFLNSCHLCKKNLHGKDIFMYRGEIAFCSNECRSKQIMMDERKEKCRSSSMELSSSPYTKDQMFSTGIMAL  >MT4G124930  MLRKRPSPMIGKLSELLVSGGRMMDTTGSPRGPLDTKMQSPRGLKNYDLGGVGLKIVAALDNNKTCEVLPKHAVCTSNLNRSGPIQIQSVKSPNRFQMDYSSMNEIDMESMEEEYTYVTCHVPNKTFTKVYYDGGEGDVRRQQGYNYINKNNVGVVRRSSPPPQIFIEPEPIFPTSSFLNSCNLCGKNLHGKDVYMYRGEKAFCSTECRSSQIMMDERKERCGSEASRSVELSSSPYARDQIFSTGILAI  >MT4G099050  MDSSIKLCYLEQDHGLASLKDMDVASYRGNSFVTKSMITMGYANNTSFKNLYVSSPRSGRFYDTRFEDHQPHFLEACSLCNKPLGGNKDIFMYRGDTPFCSEECRQEQIEIDELKEKNMNLSSSMKALRNKEHRKSNSSNKIQGQDYSFRKGTAIAA |
| ***Vitis vinifera*** |
| >LOC100244902  MESFSGKGRLAMNLSLFTNSFEPKSQVWSLDSKVSSTTKSPRNFEGGVVGLGIVAALNDLDNTHESLSSKPCKASILAATSPRSNPIPIANRAKHMPKVEKCRPSSGMMEMEMEMSESYTCVISHCGNNLIKKRVYFDDKPNGVVDDITTAVPISSTDAAYWVASEAAPPFKTADFLSSCYLCQKKLHGLDIFMYRGEKAFCSAECRSNQMVSDEYKEKCGSEAMKSFDYSVSPCSGPMLFFAGVAAA  >LOC100247517  MLRKRSRSFQKDQHMGHPTMADAVSELYFQSDVMGQKHKGNSFFSVPGLFVGLNYKGLSDSDSVRSPTSPLDFRVFSNLGSPFRSPRSSQDGQHKSWDCSKVGLSIIDSLDDGGKLSGKVLGSSESKTILFGPQMRIKTPNSPSHINFFDGSKSLPKNYASFPHTQIKSRPQKRDSDVVFEIEETPLEPEAFGRIRSCSLDSSRSFSSLTNLTKRQSNLSSGNLCPGNMTTQVSSPPQILGGNPNPDNFLPMKLNSIPASVGSGQGLIGSLSASEIELSEDYTCVISHGPNPKTTHIYGDCILECHSNDLANHNKNDEHKIGSPLIVECSDNSTPYPSNDFLSICYSCKKKLEEGKDIYMYRGEKAFCSLNCRSQEILIDEEMEKTTDDSSEKSPVSKCGEDLFETGMLAC  >LOC100252409  MNGPDWLLAFWPCVYLPLSTPSPLLLSHSVLSLDFEVVKRRGVRGAAAEEKMLLGKRPRPPMKRTTSMTEFTLDPNSVQQPPSDSQNPFKDRPKSVGFGGDPYRQPSGVDGYGFDHRFLSAVSPRNPRRYSADFVDNSHFLRACCLCKRRLVSGRDIYMYRGDSAFCSLECRQQQMNQDERKEKCSLASKKEATASTAGSEGETVAAI  >LOC100253160  MSPLNVEEEGHEGKRRPNFPGHESFKRSSVDPVGLRILTQFSHGESNLVLKSACRLRVPTPPPPSGASPESCFLKSCYLCNKELTPDKDIYMRSDEGFCSVECRNRQIVMDETKEIETSTKKILASSRHCRSAGGCETCVLLEDLRRRRKPISRHKNQALVS  >LOC100261891  MVGLSIVLEAQKGINKKTPQVINKITMMTKPTPFPSPPARWNSHSPLLPAATTFLDECFLCKQKLLPGKDIYMYKGDRAFCSVECRCRQIFMDEEETVRRDNCSLAAMKPTSASSCPSSSSPSSSTSSRLRKGTRTRAGGFAY  >LOC100264520  MLGKTSRPAIGKLAGSLISGNCTAILDVATSPRSPLDMKIQSPRGQKNYDLFSGVGLGIVAALDKSSNCRGEILAKYAVSGTNMGRSGPVPVTSGKNCGRLKVGFGELEMDSLEDYTYVTCHKPNKKSFTRVYFDGHETTAFHKHESPARFSNDVQAFPTSDFLSSCHLCRKNLHGKDIYMYRGEKAFCSNECRSRQIVMDERKEQCRSEASRSADVSSSPYTRGQIFSTGILAI  >LOC100853829  MPMKRPRVGRSSSFDDASFLSQVSPLAMGSPDLRWEKHSAIAAETASANGASDSAKILKPSELVADHZSXPKILTVASPETEKGKFDESFECPIGGFLQKCYYCKKKIHENAEVFMYGYLHAFCTSDCRDRQIIFDKELEKASAKPIEAMNEHRRISVEYIKKRPKSHKVYREYTNQLSLTTKKKAKKSHQPLSGGYPLQEAYERQRTLSTIQSSPTP  >LOC100855273  MLRNRSRAVASKQAIMGDHSSLPSPTENLTKPISFLLGSPKIFRGFISKCLPEAEDIISPTSIFDTKPFSGNPFEYEKTQASPGTFSETKRSWENLDSIGIGVALIDSDPINGEGANENFSKPNSRMVLFGSQLKVQIPHLQPSALSPAESPKSPADFGIKTRNSQLASLSPFGSLNSGIQTKDSPRIFTGMELSEEYTCVISHGPNPRTTHIFDNCIVESCCGVSALSQNNYCTFPENPNSPPENFLSCCHTCKKNLSQERDIYIYRGEKAFCSHECRSQEMLFDEEEDLEF  >LOC100261790  NCRVETKKMMLGKRARPQIKRTTSMTGITVDLGHNMVAGSNGYDPRFLAATVSPRIHRRSSGEFMETAHFLRTCGLCQRRLQPGRDIYMYRGDTAFCSLECREQQMKQDERKEKYSGMASKKEDHRHHASAQTAAASEGETLAAYDRMIRDHFYDFTCLSSTGYRKEISIRSACRVYASVANPLTSTIL  >LOC100257499  MDSTPTRQPYFIEEEDDTQASLSDMEAGFSGNHLFFSRSHSAPHRRGSLRNLSFFSASSPRSARFYDARFEDHQPHFLEACFLCNKPLGDNRDIYMYRGDTPFCSEECRQEQIEMDEATEKNRSISSIKAFRKEQKTSSTPSKSQNYPFRTGTVAAA  >LOC100264994  MAASSSSSPRSGLLYYAGCEEYPHQPHFLEACFLCQKPLGNNADIFMYRGNTPFCSKECRQEQMEFDEAKEKSWNMRSLRKSDSNKSSSNKAVRSGTVAVA  >LOC100245904  MADCGSLPSPTDKYRRPPSSSFFSSPRLFTGFSSKVFSETETMMSPTSILDSKPFSGFRNPFTNTTSTIKPSEPEPRRHWDKLDSRMIGLGIVDALTHDESDPKLSKPESRMVLFGSQLKIQIPPLPSSVLSPAESPKSPADFGIKTRNSQLGPFSPCLSQSPAKKSGFGSANSGLEAPNSPRIFTGCLSATEMELSEDYTCVISHGPNPRTTHIFDNCIVGFSASRKENDVFPESFLNFCHSCRKNIGQGKDIYIYRGEKAFCSSECRQREIMLEERMEILEGGDVYGTCS  >LOC100853335  MMLGKRARPQIKRTTSMTGITVDLGHVEAPAPADPQNPIKDVHAAARVENMVAGSNGYDPRFLAATVSPRIHRRSSGEFMETAHFLRTCGLCQRRLQPGRDIYMYRGDTAFCSLECREQQMKQDERKEKYSGMASKKEDHRHHASAQTAAASEGETLAAA  >LOC104879256  MSKKRSRVVWSSSLGDTGLFNNIQPIETPAAVSRPKPKQWERPRRSILTLGSPLKDSNDDGSDIDESFGVFLQSCFYCRRKISPKDDVFMSGCVYMRYLRAFCSESCRTTHIALEEAEQDAASRPTQATDCLTSI |
| ***Zea mays*** |
| >LOC100277785/B6TZY5/ AC197047.3_FGT001.  MMLGKRSGRPSPTARQHMRRTTSLTEFAAPDKVLADVAEEEDEELQLLPAHAEAAELEGPYGWAIGGAAAAGRADWLAAYRARAAPVLAGLRRNSADFSAAETAAFLRACGLCNRRLGPGRDTFVYRGDTAFCSLECRQQHITIEEWKEKCALATPPASDPVVPLPPRPSGAGSDKPATVLASKS  >LOC100275040/A0A096PVH6/GRMZM2G009080_T01.zma  MENDEEEEGYVKVGTRFYRVAMRPSGAVARHRLHYLESCYLCKESIARDRDVFMYKGDAAFCSEDCRDEQKDMDEALHAAARRHRLLRSPSASSSSQAAAEAAASTRPPPVMRRRPTIANLAARNPPVAAS  >LOC100273313/B4FUH9/ AC233979.1_FGT006.zma  MDVGLGGAGSSGRGGGGGRSGSGGGRDSPFALGGGGASAAAWTRLVSSGVEDELVAASGGRGGGGGRAGARAGGLPQGHFLDACFLCRKPLPSNRDIFMYRGDIPFCTEECRTEQIEMDEEMERKESTQKKKLAPRAPSPKDVESAPRPPKARAGSILAG  >LOC100279111/B6UHW5/ AC233979.1_FGT007.zma  MASAFFFDAEPDCEPSLLPTLDACALCAKPLGRDCDIFMYRGDTPFCSEECRDEQMQLDAIRARQQAARSAARRQQQHSSRTESRHQESMKVSVAT  >LOC100502301/B6TB74/ AC233979.1_FGT008.zma  MATSVACAFFFFDAAEPLGEGGRRRQRQALDACALCSKPLARNSDIFMYRGDTPFCSEDCRYEQMHHDAAATAYARHQASSRRRQQRSRGGSVSANEADVYVAS  >LOC103626680/A0A096Q414/GRMZM2G022181_T01.zma  MMWSHEPAAEQKAAASAPPTRQRSSVAKLTTASSSLANLLSAFVNTNNSPDPRPPPPRRSFDERGAVGLGIVAAMGHGAEPIAIGAAARRRAREDDESYTCVIKHVAGAGGASVRKRVYFGFGDGGGGWLVEADDAAPAPAPDFLSRCCLCDKRLDGLDIYMYRGEKAFCSSECRCQQMLLDDRAAKCGSSEALIRSGDYSVSPHSAPMAFSPSVAAA  >LOC100281547/B6SWF3/GRMZM2G033867_T03.zma  MATDSSALQASSESIAQKMGFFRVPDLLVKLSAKCLSDLDVVRSPTSPLDLKFFTGLGNKSPGDASQNQKILLGDRVGLGLVDSLTDDNPAPLGGRKVLFLGSEMRIIDNLSRKNSSTAPDQAGEVEQKDDNMSDGLMGSAMSLGDIINSEDYTRVVSRGPNPRTTHFFGDRVFELQAEQLMPVESKVDQSTSPVEDGLMSFCYFCSEKLKEAEDIYIYQGDKSFCSVECRENFMVDYEMEEAYHPASPRSPPSDGGRIFQLIR  >LOC103627751/A0A096QD31/GRMZM2G036286_T01.zma  MEPAERIESAGTALGKRPRSRVLPRTASMVTVPSAAKQGRQERGAGVPSSCSLPAACAGMVAVPGGYYYCGTSFAAVETAAFLKACGLCKRHLGPGHDTFIYRGEVAFCSQECREKQIEYDDLDERMEQTCSVTSMKEAPSVSGASGSDQSGSGGETVAAA  >LOC103643562/A0A096QFQ4/GRMZM2G040821_T01.zma  MQSLLISFSPSRSRTHTLPSSLPVKKLARSDHGSYRRKRELKKMLGRMAGPGGGGVSEQRQREAAFDFGRHTGTAAAVPSSSPHTPMVPPKLFLAAGSGGVVSGEEAFSPPEELAGGVVVMVSPTSTLQTPTGSASPTSAATSTAVPFSRRGATSPSSPGGDDRRRCKSRSRIRSQRPWEAWPVGVGLAGALSGDADALPPVATVLTGQRIRPWPCRSSASSVLGLGSPESRSRCRGVLSPRKMMEASEEDYTRVIARGPNPRTTHIFDDRVVVVEDEFLRWCHGCSKDLGQGNDIFMYRGEMAFCSHECRYRVMLLLDEEGESC  >LOC103647382/A0A096QMY7/GRMZM2G051752_T01.zma  MVGKRERDCKNPVRRTTSMTEFALAAVMEEDDEEAQLPDNGSSSSRGGGQHQQDWLSALGGGAAAAQEDWIAAYRARAAPAKAGLRRNSADYSAVETAAFLRACGLCRRRLGPGRDTFVYKGEAAFCSLECRERHITQEEWKDKCAATSIKDAGAAKVVSGRRRAGEGKAGGTVAAA  >LOC103627565/A0A096QS87/ GRMZM2G057717_T01.zma  MEFTSSYFHAFGNPDLAAVVSGDGGSAQAHRPRRSTDGAKAEDGRSPTTTTARRAPSMFCVPDTEAEEPNGFLDECTLCRKALCGDIFMYRGDTPFCSDDCRREQIDMDRIRHRRKKQQALIAQQQQQQQAAAAAAMAQRDRRTQRQLQPQH  >LOC103653929/K7U358/GRMZM2G064626_T01.zma  MEFTSSSYFHASGNPDFAAVFSDGGGSAQAHRPRRSTDDGTKAEDGRSPTTARRPPSAFCVPDTEAEEPNSFLDECTLCRKALCGDIFMYRGDTPFCSDDCRREQIEMDRIRHRRKKQHALIQQQLQLQQQQQAAAAAMAQKDQRAQRRQLQPQH  >LOC103646017/A0A096RIX3/GRMZM2G098099_T01.zma  MKGGGAMRPSPMFYVHEADVVQIHHFLEECSLCAKSLSGDIFMYRGDTPFCSEECREQQIEVDRAKHRRKKRAAAHALSARSREHRHQQQLQQHHHQQQQPQPRNAGMDTRHPWVDAGFARPRAPALRV  >LOC100275491/B6SU44/ GRMZM2G129879_T01.zma  MLPRRQSIFHLGEEGGAAAVHHRVSVVGAASSSMAMGGGRRARERERLVVGLQILVHHHHHGRHGHGHAHAANVVLKQMVRPRAAVAAAAGSRHGHGSHAFSCSFLKACYLCKRELSPDKDVYMYRGDQGFCSEECRWQQILVDEAREREAAAVMSNKELQRRAQARHHSPHRTPMPIRGRPPRKTLAVA  >LOC103653928/K7UY88/GRMZM2G164098_T01.zma  MRRCRARLHKTTTTFFFLPSQQVKRNPPVGTSQPKKPMEDYYYYFTSWGTVGSVEPTGFPAHTLPRNPSSASPKTRRASRGDADAGERLHHYYLDACFRCGRHLGGNKDIFMYRGDTPFCSDECRQQQIEDDEAREKKRSRQHAAATATATATKRERERRS  >LOC100276146/K7W4Z7/GRMZM2G171752_T01.zma  MSTPTRKRPRKTNGSTSSSSSAMRRTTSLSDLAPPPDLSGRPKTRAARGHAVAGPGTAWGAEMTMTHSADFLPAMETAAFLKACGICNRRLGPGRDTFIYMGEVAFCSQECRQQQMNLDELMEKKCSTPAGGGGGGVGGGGGGGSDQPGKSSTVAAA  >LOC100382411/A0A096T0K9/GRMZM2G176748_T01.zma  MAMSEDYTCVISRGPNPRTTHIFDDCIVESCGDLLADKPAAGGAAAAACGFFSSCHACNKQLGHGNDILIYRGHKAFCSSECRYQETLLDEAVDGEFALGDGGKGGGRC  >LOC100277222/B6TR19/GRMZM2G322817_T01.zma  MATSVACAFFFDAEPLGEPERHALDACALCAKPLSRNSDIFMYKGDTPFCSEDCRYEQMHHDAAYARQAASSRRKQQQSQRSRGASSVGAKADVSVAS  >LOC100279084/B6UHK2/GRMZM2G335685_T01.zma  MMASSSSFSSFFGIEPLDGGDGDAYRRAMDACSLCGKRLAGDRDIFMYRGDTPFCSEECRHHQMARDDFGAALKTEPRPARRGERRRRRHEAPAAAAAEPARVPLAPNVPAVVI  >LOC103636115/A0A096TP16/GRMZM2G416156_T01.zma  MARKLEIPQWKHDGLPSPTSPLDRASPRGWRHRDAVGGVGLGILAALEAEGPLPGAGAAAAPRVSIPRRAARLEVSELACSGRCATSLCGGPVSGRAGGGGGAAFRVAEFLSCCDMCRRALDGKDVFMYRGERAFCSMECRYHAIVSDEFREAEEEKERKRRAAAADVPRKSAAEMVAGSPCSGGGQIFFTTGIVVA  >LOC100276881/B6TKC9/GRMZM2G425482_T01.zma  MARRLEIPQWKADGLPSPTSPLDRASASPXGWRHRDAVGGGVGLGILAALEAEGPPQPAPRVSIPRRAARLEVSELGCSGRCATSLCGGVGPAPARPGGGAAFRVAEFLSCCDMCRRALDGRDIFMYRGEKAFCSMECRYHAMVSDEFQQXKERKRRAAVSRTSAAEMAGSPCSGGGGGGQIFFTTGIVAA  >LOC103643326/A0A096TVQ1/GRMZM2G452887_T01.zma  MSSRHIPTKRSTRVVVPYNPETSVGLQAALVAPASAHLHVAEPVRRSARIITLQAVTTTDRRRNTPYCMMTSSRFCVDDGSLFCKLCDRRMHDRIIYMYMSMGFCTEECRNEYFLDYRCRLTMATVEAEGRSEARKPVEAAAATTEGQGVGYRRIFFTCAEHSS  >LOC103631809/GRMZM2G496991_T01.zma  MSAGHPPPLPGSAAGGAPHQWTLLQSRVRLLPRNTFLLRVHRRESETASYINASPAHAEYVMIPRAPGLFRLEEGVGARASTSAPAAMAAPAGDPQLVGLRLIIQPSPRKQQLPAVLRKSAVRIVNPATASAVKCHDDDDSGRRVFAGLEFLKRCSCCHRDLDATMDVFVYKGEQGFCSAECRCRHIAKEERREMEMLLRKRRDAFHRRHAAAAAPKMQASDRRITLQITAAR  >LOC100272399/B4FNF0/GRMZM5G844703_T01.zma  MMLGKRSGRPSPAARQHMRRTTSMTEFAAPHVLVDVAEEEEDEELQLLPAHAEATGEAAEQEDPYGWAIGGAAAAGRADWLAAYRARAAPALAGLRRNSADFSAAETAAFLRACGLCNRRLGPGRDTFMYRGDTAFCSLECRQQHITIEEWKEKCALATPPTAPASDPVVPLPPSGAGSDKPGTLAAAS  >LOC100278632/A0A096UFP8/GRMZM5G874697_T01.zma  MGAAGPAGVHFLDACFLCRKPLAGNRDIFMYRGDTAFCSDECRSAQMAADEAAEKRRKARAVTRGAMFAREAEGPQESGKVRAGSSILAL  >LOC100276205/A0A096PYT2/GRMZM2G014009_T01  MDLGLSVAGRTGSGGGGGWRGSGGGASAAAWTRLVSSGVEDELVAASGGGGGGGGGRAGARAGGLPLGHFLEACFLCRKPLPSNRDIFMYRGDIPFCTEDCRREQIEMDEEMERKESKPKNVAPRGAPSPKDVDSPPRPPKARAGSILAG  >LOC100277951/B6U295/GRMZM2G056988_T01  MAGLSVLLENHSKSYTGKAAAAQIISKATLVTTHGPNHQHQQRKIPVSAAAAGSFLQRCCLCHKELAEGMDIYMYKGDRAFCSEECRCRQIFMDEDATTVRGRRRVKTRQGWEREWET  >LOC100274019/A0A096RJK8/GRMZM2G099166_T02  MVESSGHRSTVPAAVGFFRVPGLFVRLSGKGGASNAVDPEDSVWSPTSPLDLKHVIRCSPPRACLGLADALTADGTGSLHSGGRSSFVDSIKPFLERALPKAACGKEAAAAASSSAGVVAATLGKQASEYADCEEYTCVISRGADPRTTHILAGETVEVRRGDVGGGCRKVVFIIEPLALSDRQPRASSSSSPAAPARVVASGRCCCCMKRLLEDRDIFIYLGEKAFCSDECRNGFIEEAAEEELMILDPARNL  >LOC100272233/B4FI24/GRMZM2G166692_T01  MMVGKRERDCKNPMRRTTSMTEFAPPDALAAVMEDEEGPQLSDDSSRDGGQQDWLSALGGGGGGVGGAAAQEDWLAAYHARAAPARAGLRRNSADYSAVETAAFLRACGLCRRRLGPGRDTFMYKGEAAFCSLECRERHMTQEEWKDKCAVTSIKDAAAGSAKVKGRRAGSGKAGGTVAAA  >LOC100278490/B4G1W1/GRMZM2G080959_T01  MGGSGRGAHFLDACFLCRKPLAGNRDIFMYRGDTAFCSDECRSAQMAADEAAAAKAKAKAKARAVSRGALLTAREAEGPQRRGKVRAGSVLAL  >LOC100278820/B6SI69/GRMZM2G024517_T01  MAAHLASAFFFAAEPVGEPGVHALDACALCAKPLARDSDIFMYRGDTPFCSEECRYEQMQLDAIRARQPYASGTEARRGHREASKVSAVA  >LOC100273216/B4FTN6  MASSSFFFFDAEPVYDYEPSLLPTMDACALCAKPLGRDCDIFMYRGDTPFCSEECRGEQMRLDAVRARQAARSAGRRQQQQQYSSRTESRHQETMEVSVAT  >LOC100275085/B6SNB0  MASSSFFFFDAEPVYDYEPSLLPTMDACALCAKPLGRDCDIFMYRGDTPFCSEECRGEQMRLDAVRARQAARSAGRRRQQQQQYSSRTESRHQETMEVSVAT  >LOC100275205/B6SQ10  MADNAGLVGLLLEQHQLRPPPPAAAASASRAPAQITSKATRPLLLATAKNDVVDVQKGASSAAASFSRFSSSSSRAPAPTVSLTTACPFLQRCFFCHGELSDGRDIYMYRGERAFCSEECRCRHILAEEDDDDTTTSVGVVAAAADCSTQLRHQALAASFTF  >LOC100276685/B6TGG8  MSCPAPDTAAAAAATAMSVPCSRFQFRRGDLKSGPEEATRSDTGAYLVSNGNAKRNSFDFGKLXGPGSLPXPASRRFVGSVSASEIEQSEDYTRVIARGPNPKTTHIFGDCILEPHTAGASDDEAAAVESEDGAAAGRYFVVKCNAEADGFLSSCFACKKKLDGNDIYIYRGEKAFCSADCRDQEIQLEEEEAENSTSGASPLSSCSSSSFHDDDIFMAGMVVAT  >LOC100277998/B6U2Z5  MADNAGLVGLLLEQHQLRPPAAAAASRTPAQITSKATRPLLATAKNDVVDVQKGASSAAASFSRSSSSSSSRAQAPTVSPTTTACPFLQRCFFCHGELADGRDIYMYRGERAFCSEECRCRHILAEEDDDDTTTSVGVVEAAAAADCSTQLRHQALAASFTF  >LOC100303941/B6T172  MEPAERIESGTALGKRPRSRVLPRTASMVTVPSAAKQGRQERGAGVPSSCSLPAACAGMGAVPGGYYYYCGGPFAAVETAAFLKACGLCKRHLGPGHDTFIYRGEVAFCSQECREKQIEYDDLDERMEQTCSVTSMKEAPSVSGASGSDQSGSGGETVAAA  >LOC100274617/B6SGH2/GRMZM2G016948_T01.zma  MEPAERIESGTALGKRPRSRALPRTPSMVTVPSAAKQGRQERRAASLPTGTGAAGAMPVGYCYYYGGSFAAVETAAFLKACGRCSRRLGPGHDTFIYRGEVAFCSQECREKRIEYDERMEKTCCSLTSIKEAPSVSGASGSDQSGSGGETVAAA  >LOC100304004/B6TFU6  MASAFFFDAEPDCEPSLLPTLDACALCAKPLGRDCDIFMYRGDTPFCSEECRDEQMQLDAIRARQAARSAARRGQQQQQHSSRTESRHQESMKVSVAT  >LOC100277698/B6TYQ3/GRMZM2G163067_T01.zma  MAHYHPVSADTYQDLEAGFSGHSSSPLKPAASPRRPGRMFCDPCDDGDELHGRHHYLDICFRCRKLLSGNRDIFMYRGDLPFCSEECRQEQIEIDEAREQRLKQTGRAEQQRQRQQKQSPQRIPIWAW  >LOC100280020/B8A1C4  MSVPCSRFQFRRGDLKSGPEEATRSDTGAYLVSNGNAKRNSFDFGKLPGPGSLPVPASRRFVGSVSASEIEQSEDYTRVIARGPNPKTTHIFGDCILEPHTAGASDDEAAAVESEDGAAAGRYFVVKCAAEADGFLSSCFACNKKLDGNDIYIYRGEKAFCSADCRDQEMQLEEEEAENSTSGASPLSSCSSSSSFHDDDIFMAGMVVAT  >LOC103655213  MVGGGRRGGAAEEVKLNTGNVFAALESLKKKKKGDKGGRGTLGKQASEYADCEEYTCVISRGADPRTTHILAGETVEVRRGDVGGGCRKVVFSIEPLALSDRQPPTSSSSSLAAPARVVASGRCCCCMKRLLEDRDIFIYLGEKAFCSDECRNGFIEEAEEEELMILDSARNL  >LOC103643065  MLRRMVPDPSSPDSSGGGGGGDARPRGGGGGALFAVPRLFVGFAAKRVAPDGESSRSXTSPLDPKALLLRSPRSPRRTWGAPGLVDALAADTAAANCLLSPRLRLVRQHSSPPKGCGGHSQPELGKTMSCCPAPDTAAAGAAAGAGMSVPCPCSRFQLRHGDLKSGPEATGSADTGAHLASKRHSFDLGKLPGPGSLPVPASASASSNAARRFVGSVSASEIEQSEDYTRIIARGPNPKTTHIFGDCILEPRTVGCGDSKAVESGEGGAGCYFVVKRAAPGAGDFLSSCFACKKKLEGNDIYIYRGEKAFCSANCRDQEIQLEEEAENNTSSGSPGSSSSSSSSSNDDIFMAGMVVAT  >LOC103654017  MYCYASGPFINCLMWKEGVMIFCCFCSEKLKEGEDIYIYQGDKSFCSTECRENFMVDEMEGKGGWKHLFCMGAAWRELIGRKQTLTRFLNVPREKGISWDLLFIQGKKERFVQGGNDF  >LOC100274866  MATSVACAFFFDAEPLGEPGRHALDACALCAKPLSRNSDIFMYKGDTPFCSEDCRYEQMHHDAAYARQAASSRRKQQQSQSQRSRGASSVGAKADVSVAS  >LOC100277836  MADNAGLVGLLLEQHQLRPPAAASRAPPQITSKATRPLLATAKNDVVDKGSSAAASFSRSSSSSSSRAPAPTVSPTTACPFLHRCFFCHGELADGRDIYMYRGERAFCSEECRCRHILAEEDDDDTTTSVGVVAAAADCSTQLRHQALAASFTF  >LOC100502468  MLPRRQSIFHLGEEGGAAAVHHRVSVVGAASSSMAMGGGGGRRARERERLVVGLQILVHHHGRHGHGHAHAANVVLKQMVRPRAAAGSRHGHGSHAFSCSFLKACYLCKRELSPDKDVYMYRGDQGFCSEECRWQQILVDEAREREAAAVMSNKELQRRAQARHHSPHRTPMPIRGRPPRKTLAVA |
