# Supplementary material for: Comprehensive Evolutionary and Expression Analysis of FCS-Like Zinc finger Gene Family Yields Insights into Their Origin, Expansion and Divergence
Source: PLoS One. 2015 Aug 7;10(8):e0134328. doi: 10.1371/journal.pone.0134328 (PMC4529292; doi:10.1371/journal.pone.0134328)
Supplement: S6 Table — (DOCX) [file pone.0134328.s014.docx]

| **S6 Table. Novel motifs identified from FLZ proteins using MEME** | | |
| --- | --- | --- |
| **Motif name** | **Sequence logo** | **E value** |
| **Motif 1** | 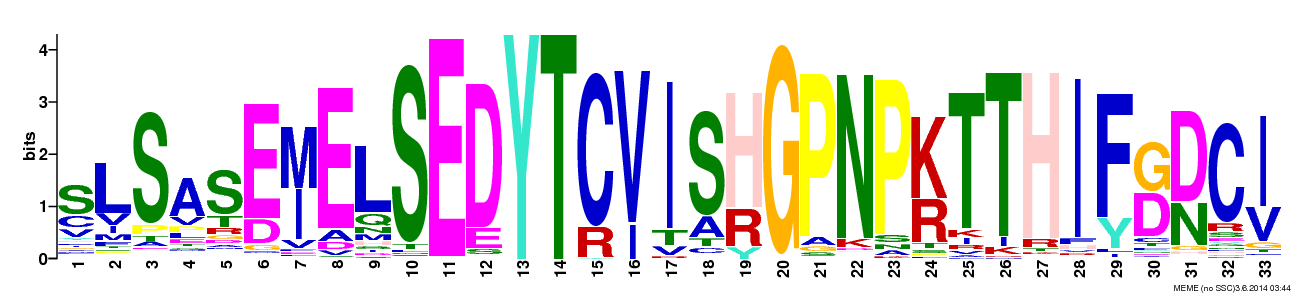 | 1.7e-4013 |
| **Motif 2** | 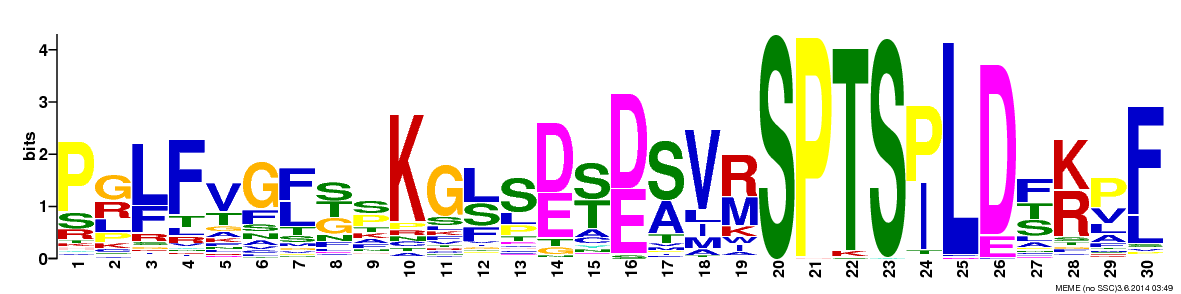 | 1.6e-1176 |
| **Motif 3** | 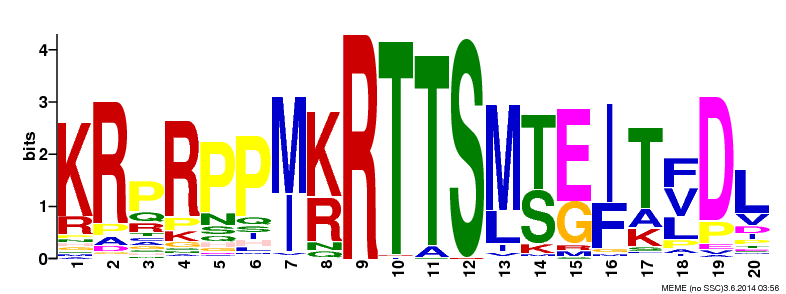 | 6.3e-1047 |
| **Motif 4** | 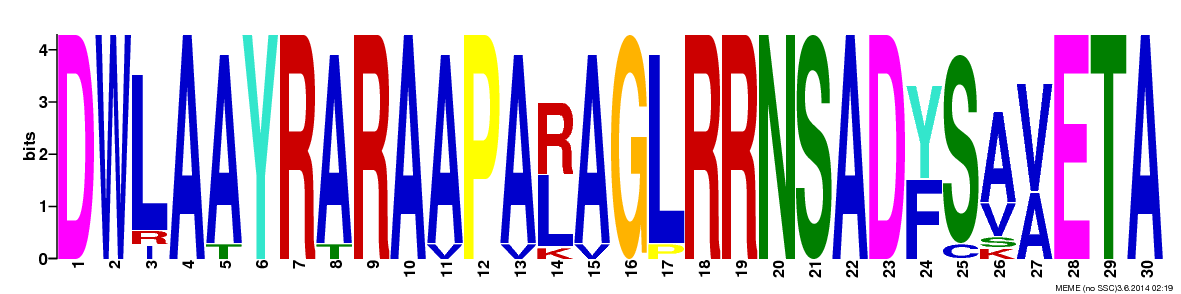 | 3.9e-225 |
| **Motif 5** | 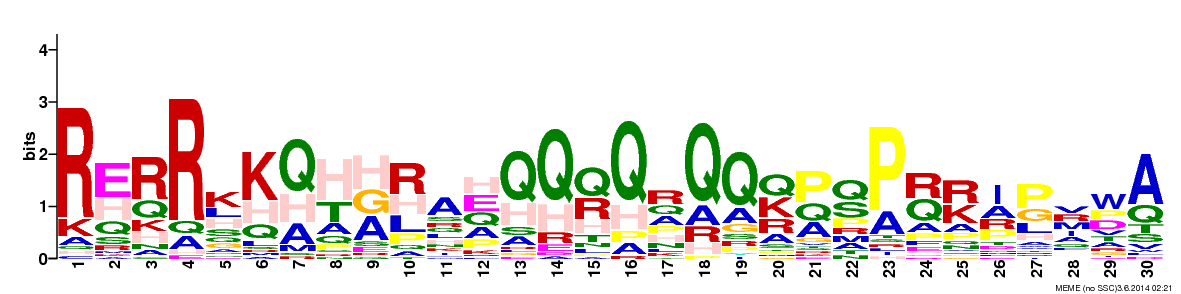 | 3.6e-159 |
| **Motif 6** | 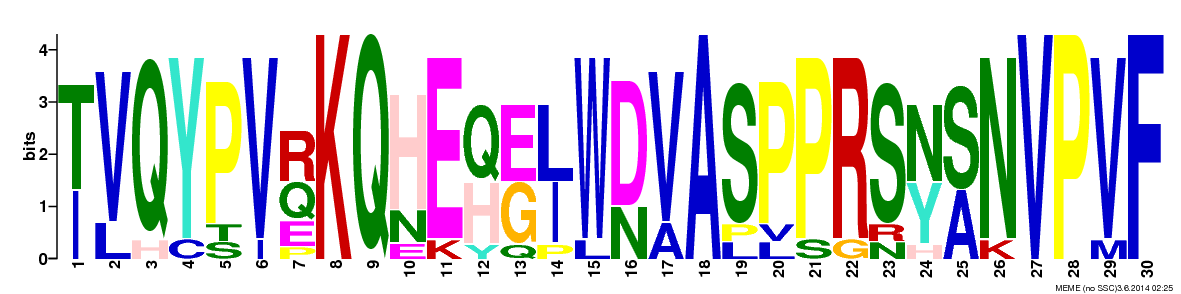 | 1.4e-157 |
| **Motif 7** | 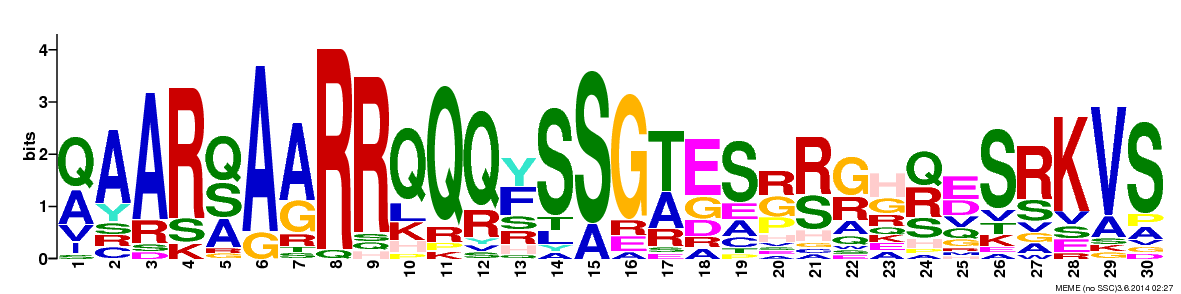 | 1.3e-121 |
| **Motif 8** | 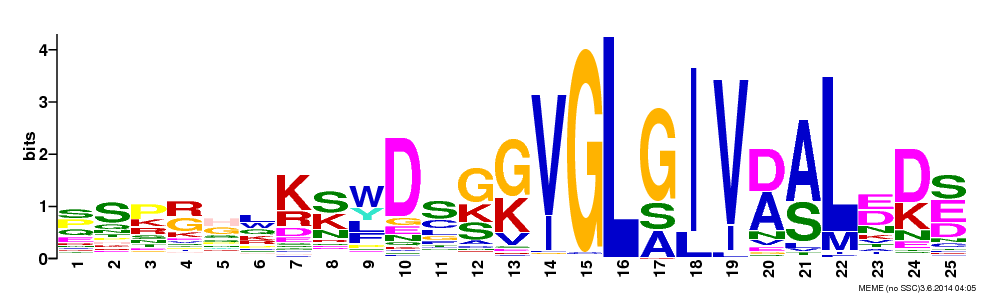 | 3.5e-1435 |
| **Motif 9** | 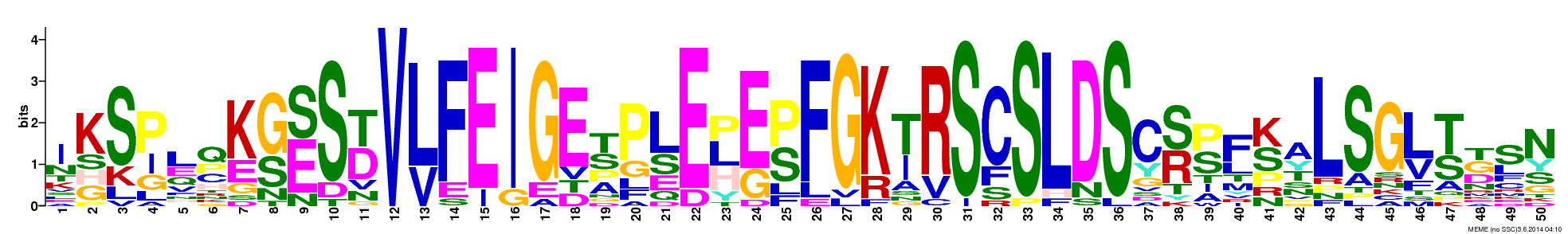 | 2.0e-281 |
| **Motif 10** | 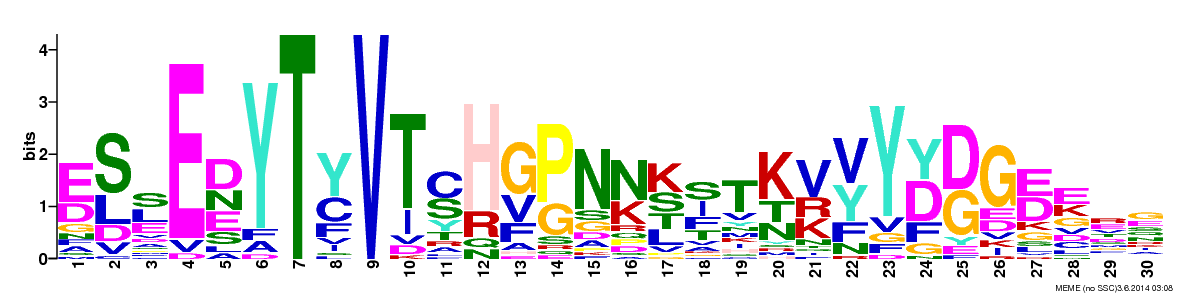 | 3.1e-232 |
| **Motif 11** | 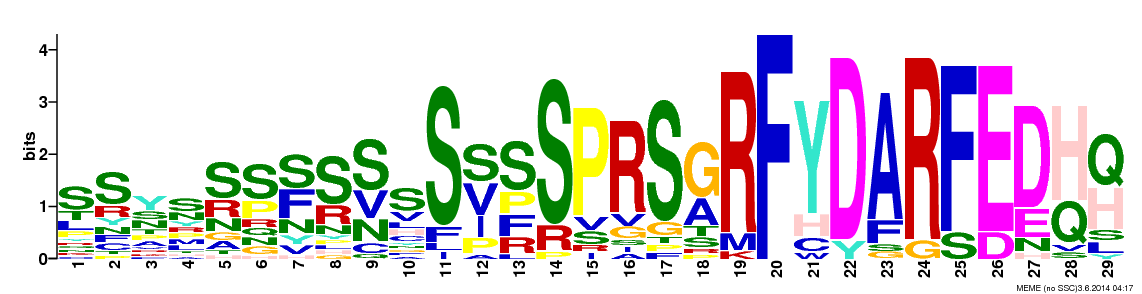 | 2.4e-168 |
| **Motif 12** | 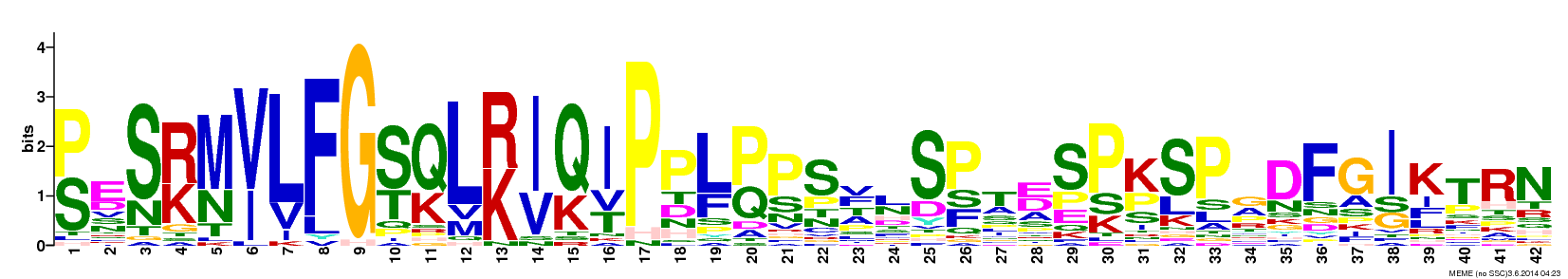 | 3.8e-313 |
| **Motif 13** | 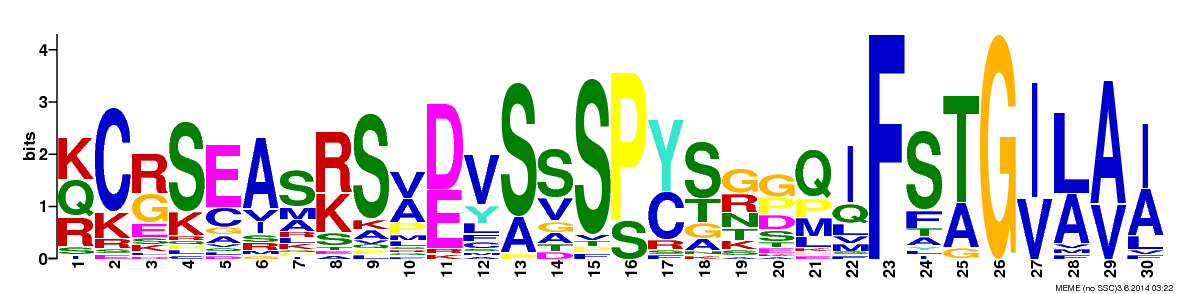 | 2.0e-277 |
| **Motif 14** | 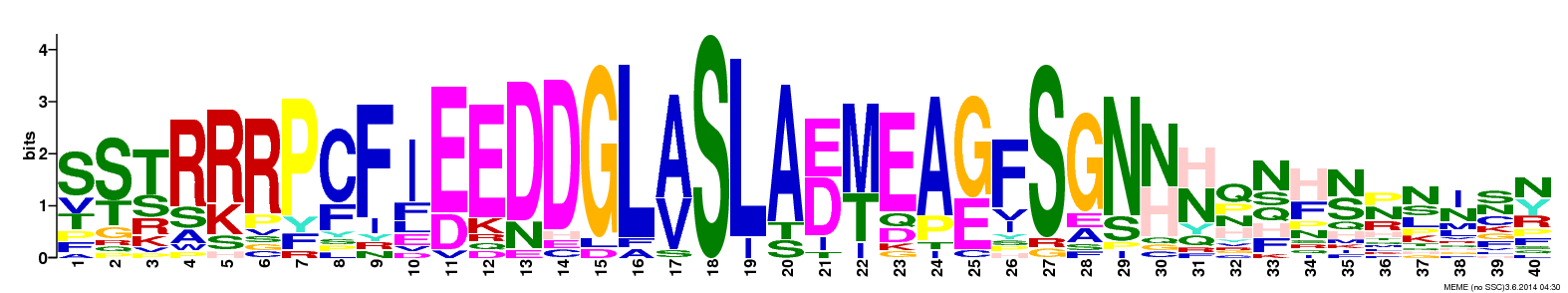 | 4.9e-270 |
| **Motif 15** | 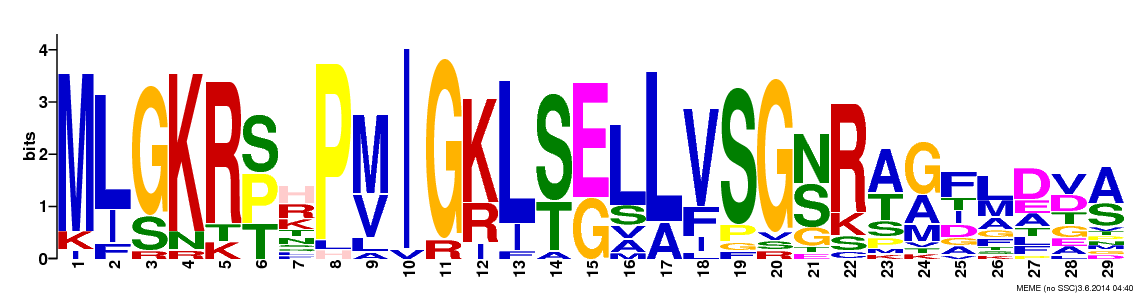 | 1.4e-195 |
