# Supplementary material for: Comprehensive Evolutionary and Expression Analysis of FCS-Like Zinc finger Gene Family Yields Insights into Their Origin, Expansion and Divergence
Source: PLoS One. 2015 Aug 7;10(8):e0134328. doi: 10.1371/journal.pone.0134328 (PMC4529292; doi:10.1371/journal.pone.0134328)
Supplement: S7 Table — (DOCX) [file pone.0134328.s015.docx]

**S7 Table. Homology detection of novel motifs identified in MEME analysis**

(Three best hits for each motif is shown)

| **Motif** | **Hit (PDB id)** | **Prob.** | **E-value** | **P-value** | **Score** |
| --- | --- | --- | --- | --- | --- |
| **Motif 1** | T-cell receptor gamma chain (1ypz_F) | 75 | 4.5 | 0.00014 | 24.1 |
|  | Immunoglobulin A1 heavy chain (3m8o_H) | 68.3 | 4.1 | 0.00013 | 25.9 |
|  | IG MU chain C region secreted form (4ba8_A) | 59.0 | 13 | 0.00039 | 20.0 |
| **Motif 2** | DNA-directed RNA polymerase II subunit RPB1 (4jxt_B) | 23.4 | 26 | 0.00081 | 16.2 |
|  | Phosphorylated peptide from C-terminal of RNA polymerase II (1p16_C) | 21.5 | 30 | 0.00093 | 16.0 |
|  | EIAV capsid protein P26 (1eia_A) | 17.1 | 49 | 0.0015 | 21.3 |
| **Motif 3** | Cyclin-dependent kinase inhibitor 1 (2zvv_Y) | 81.7 | 0.27 | 8.4E-06 | 21.1 |
|  | Pfadf1, cofilin/actin-depolymerizing factor homolog 1 (3q2b_A) | 28.0 | 17 | 0.00052 | 17.9 |
|  | V1AR, vasopressin V1A receptor (1ytv_M) | 27.4 | 12 | 0.00036 | 19.4 |
| **Motif 4** | AP endonuclease (3ngf_A) | 47.9 | 3.7 | 0.00011 | 21.0 |
|  | Tetrahydrodipicolinate acetyltransferase (3bv8_A) | 24.0 | 23 | 0.00071 | 19.3 |
|  | HTRA, putative serine protease (2l97_A) | 22.0 | 29 | 0.00089 | 17.3 |
| **Motif 5** | Maltose-binding protein (3iot_A) | 53.6 | 3.1 | 9.4E-05 | 27.4 |
|  | Maltose-binding periplasmic protein (4feb_A) | 44.1 | 5.3 | 0.00016 | 25.9 |
|  | Maltose-binding periplasmic protein (4feb_A) | 35.9 | 7.2 | 0.00022 | 25.2 |
| **Motif 6** | Wiskott-aldrich syndrome protein family member 2 (2a40_C) | 82.3 | 0.32 | 9.9E-06 | 23.6 |
|  | YFLH protein (3d0w_A) | 40.2 | 4.3 | 0.00013 | 23.2 |
|  | P7TM2, P7 polypeptide (2k8j_X) | 38.0 | 11 | 0.00035 | 17.7 |
| **Motif 7** | CG4944-PC, isoform C; protein-protein complex (2ff6_H) | 12.6 | 32 | 0.00097 | 15.7 |
|  | Golgi to ER traffic protein 2 (3sjd_D) | 8.4 | 82 | 0.0025 | 16.0 |
|  | Golgi to ER traffic protein 2 (3zs9_C) | 6.9 | 1.4E+02 | 0.0042 | 14.6 |
| **Motif 8** | Mannosyl-oligosaccharide 1,2-alpha-mannosidase (4ayo_A) | 39.2 | 3.6 | 0.00011 | 25.9 |
|  | Methionine synthase (B12-independent) (3rpd_A) | 35.1 | 17 | 0.00054 | 20.5 |
|  | Anti-sigma F factor (1th8_A) | 28.4 | 4.1 | 0.00013 | 21.3 |
| **Motif 9** | Probable insulin-like peptide 5 A chain (2wfu_A) | 27.2 | 5.2 | 0.00016 | 20.1 |
|  | NADH dehydrogenase I subunit E (2lxr_A) | 25.9 | 22 | 0.00068 | 21.6 |
|  | SWI5, zinc finger DNA binding domain (1zfd_A) | 24.7 | 21 | 0.00063 | 17.8 |
| **Motif 10** | Protein (immunoglobulin), virus-antibody complex (1qgc_4) | 77.5 | 1.4 | 4.4E-05 | 27.6 |
|  | IG gamma-2A chain C region, A allele (3zo0_A) | 72.9 | 2.6 | 7.9E-05 | 23.3 |
|  | IGY FCU3-4, immunoglobulin (2w59_A) | 72.4 | 4.1 | 0.00013 | 22.6 |
| **Motif 11** | DNA replication protein DNAC (3ec2_A) | 44.2 | 5.5 | 0.00017 | 22.7 |
|  | Hypothetical UPF0131 protein PH0828 (v30_A) | 23.7 | 6.4 | 0.0002 | 21.6 |
|  | Cytosine/guanine deaminase related protein (2i9u_A) | 23.0 | 19 | 0.0006 | 19.3 |
| **Motif 12** | Putative nucleotidyltransferase (1ylq_A) | 33.9 | 9.9 | 0.0003 | 22.8 |
|  | Hypothetical protein HI0073 (1no5_A) | 27.8 | 21 | 0.00065 | 21.3 |
|  | RE55538P, BEN domain (4ix7_A) | 21.3 | 33 | 0.001 | 21.3 |
| **Motif 13** | Photosystem 1 reaction centre subunit II (1jb0_D) | 24.8 | 17 | 0.00053 | 21.9 |
|  | Photosystem I subunit II (4kt0_D) | 23.6 | 19 | 0.00058 | 21.8 |
|  | Photosystem I reaction center subunit II (2wsc_D) | 18.2 | 32 | 0.001 | 21.9 |
| **Motif 14** | Glucan endo-1,3-beta-D-glucosidase (3ur8_A) | 59.5 | 3 | 9.2E-05 | 27.4 |
|  | CAG38821; archeal virus, viral protein (3ur8_A) | 50.8 | 2.5 | 7.6E-05 | 24.2 |
|  | Brassinosteroid insensitive 1-associated receptor (4mn8_B) | 43.8 | 5.4 | 0.00017 | 25.0 |
| **Motif 15** | Protein FDRA, predicted actyl-COA synthetase (3dmy_A) | 48.6 | 4.4 | 0.00014 | 27.7 |
|  | VP12.5, KP6 killer toxin subunit beta (4gvb_B) | 40.0 | 6.5 | 0.0002 | 21.6 |
|  | Serine/threonine-protein kinase PAK 4 (4l67_B) | 29.3 | 13 | 0.0004 | 16.8 |
